# Genealogy of an ancient protein family: the Sirtuins, a family of disordered members

**DOI:** 10.1186/1471-2148-13-60

**Published:** 2013-03-05

**Authors:** Susan Costantini, Ankush Sharma, Raffaele Raucci, Maria Costantini, Ida Autiero, Giovanni Colonna

**Affiliations:** 1 “Pascale Foundation” National Cancer Institute - Cancer Research Center (CROM), via Ammiraglio Bianco, 83013, Mercogliano, Italy; 2 Doctorate in Computational Biology, Second University of Naples, Naples, Italy; 3 Laboratory of Animal Physiology and Evolution, Stazione Zoologica Anton Dohrn, Villa Comunale, 80121, Naples, Italy; 4 Department of Biochemistry, Biophysics, and General Pathology, Second University of Naples, via Costantinopoli 16, 80138, Naples, Italy

**Keywords:** Sirtuins, Sirtuin disorder, Sirtuin phosphorylation, Intrinsically disordered proteins, Sirtuin mechanism of action, Dynamic phosphorylation, Phospho-isomers

## Abstract

**Background:**

Sirtuins genes are widely distributed by evolution and have been found in eubacteria, archaea and eukaryotes. While prokaryotic and archeal species usually have one or two sirtuin homologs, in humans as well as in eukaryotes we found multiple versions and in mammals this family is comprised of seven different homologous proteins being all NAD-dependent de-acylases. 3D structures of human SIRT2, SIRT3, and SIRT5 revealed the overall conformation of the conserved core domain but they were unable to give a structural information about the presence of very flexible and dynamically disordered regions, the role of which is still structurally and functionally unclear. Recently, we modeled the 3D-structure of human SIRT1, the most studied member of this family, that unexpectedly emerged as a member of the intrinsically disordered proteins with its long disordered terminal arms. Despite clear similarities in catalytic cores between the human sirtuins little is known of the general structural characteristics of these proteins. The presence of disorder in human SIRT1 and the propensity of these proteins in promoting molecular interactions make it important to understand the underlying mechanisms of molecular recognition that reasonably should involve terminal segments. The mechanism of recognition, in turn, is a prerequisite for the understanding of any functional activity. Aim of this work is to understand what structural properties are shared among members of this family in humans as well as in other organisms.

**Results:**

We have studied the distribution of the structural features of N- and C-terminal segments of sirtuins in all known organisms to draw their evolutionary histories by taking into account average length of terminal segments, amino acid composition, intrinsic disorder, presence of charged stretches, presence of putative phosphorylation sites, flexibility, and GC content of genes. Finally, we have carried out a comprehensive analysis of the putative phosphorylation sites in human sirtuins confirming those sites already known experimentally for human SIRT1 and 2 as well as extending their topology to all the family to get feedback of their physiological functions and cellular localization.

**Conclusions:**

Our results highlight that the terminal segments of the majority of sirtuins possess a number of structural features and chemical and physical properties that strongly support their involvement in activities of recognition and interaction with other protein molecules. We also suggest how a multisite phosphorylation provides a possible mechanism by which flexible and intrinsically disordered segments of a sirtuin supported by the presence of positively or negatively charged stretches might enhance the strength and specificity of interaction with a particular molecular partner.

## Background

The Sir genes (*Silent Information Regulator*) have been found to be factors critical for silencing in plants where they code for four proteins: Sir1p, Sir2p, Sir3p, and Sir4p [1]. In particular, Sir2 comprises the founding members of a protein family named Sirtuins. In humans as well as in all mammalia this family is composed by seven different homologous proteins being all NAD-dependent de-acylases [2,3]. Many studies have determined the cellular location of human SIRT families and their biological functions [4]. SIRT1 is defined as a nuclear protein and is involved in inflammation metabolism and neurogeneration, and deacetylases PGC-α, FOXOs, NFκB and other substrates [5]. However recent studies conducted on mouse propose also its cytoplasmatic presence suggesting a nucleo-cytoplasmic shuttling upon oxidative stress [6,7]. SIRT2 is generally localized in the cytoplasm, and is involved in cell cycle and tumor genesis. SIRT3 is known as a mitochondrial protein, but it seems also to act as a nuclear protein transferred into mitochondria during cellular stress [8,9], however, its functional importance is still controversial [10]. Also SIRT4 and SIRT5 are present in the mitochondria, and have functions of ADP-ribosyl-transferase enzyme [11], and deacetylase, respectively [12]. SIRT6 and SIRT7 are nuclear proteins, associated with heterochromatic regions and nucleoli, respectively [13,14].

The available data show that the activity levels of Sirtuins are regulated at transcriptional, posttranscriptional, and posttranslational levels [15] and that the N-and C-terminal extensions are the targets of these posttranslational modifications. Mechanistically this is often used to explain the catalytic activity by interaction of these extensions with the catalytic core domain [16], the molecular recognition or complex formation by the binding of interacting partners, the cellular localization by the presence of truncated or not truncated forms [17-20]. We have recently modeled the whole 3D-structure of SIRT1 [21] that unexpectedly emerged as a member of the intrinsically disordered proteins with long disordered N- and C-terminal regions of about 250 amino acids each and a compact catalytic core globularly shaped with an organized allosteric site. The terminal regions have also numerous phosphorylation sites and various stretches of similarly charged residues. All this peculiar structural organization seems to explain well the different numerous functional activities exerted by this protein [21]. Therefore, the first raising question is whether these structural and functional properties are also shared by the other members of this family in humans as well as in other organisms. The abundance of the cellular partners and the complexity of the metabolic network in which the Sirtuins are involved is the most important reason for which this protein family is so largely studied in humans. However from an evolutionary point of view it is still unclear when and how these proteins have acquired a key role as modulator of so many biological processes. Human sirtuins have been characterized by a catalytic point of view and poorly from the point of view of their molecular recognition where the phosphorylation sites certainly play an important role [22]. Given the gap between the number of putative phosphorylation sites found and the number that have been experimentally characterized by mass-spectrometric studies, but only for human SIRT1 and 2 [23-27], we believe that computational methods can play a valuable role in elucidating consequences of posttranslational phosphorylation in all the sirtuin family, particularly when these sites are phylogenetically conserved as we have found. A general reason is that the metabolic importance and the functions performed by sirtuins in humans have focused the bio-medical research on the functional aspects, leaving out the molecular basis of their action. The catalytic activity has been well studied but, despite it is recognized that there is an effect due to the terminal ends no serious attempt has been made to understand these relationships. However, numerous questions remain regarding the mechanisms through which disordered proteins perform their biological function(s) [28]. Therefore, we have reconsidered the evolutionary aspects taking into account the presence of disordered terminal arms in this protein family to understand how they shared their structural properties and have also analyzed the effect of evolution in drawing their evolutionary histories. Furthermore, we have performed a phylogenetic analysis of the putative phosphorylation sites in all the family as well as specifically on human sirtuins to have a feedback of their cellular locations. Therefore, the major goal of our study has been to unravel the intriguing interconnections between intrinsic disorder and functional features of the Sirtuin family to rationalize future studies on human members.

## Methods

### Sirtuin sequences

The search for Sirtuin sequences was made in all the databases of nucleotide and protein sequences in order to select all the sequences comprising the catalytic site but also the N- and C-terminal regions. The scattered presence of SIRT sequences of the various classes in databases as well as the need of select all the sequences comprising the catalytic site and the N- and C-terminal regions have reduced the number of the useful Sirtuins to 150 sequences. Multiple alignment of the Sirtuin sequences from different organisms were made by using CLUSTALW program [29]. An unrooted phylogenetic tree was constructed on full-length amino acid sequences by the “neighbour-joining” method and bootstrap values were calculated using SplitsTree 4 [30]. We used BIOEDIT program and the web-tool FASMA, developed by our group for the analysis of residue composition [31].

### Sequence stretches analysis

The sequence stretches of similarly charged amino acids were taken into account only beginning from at least three residues. Only in case of very long stretches (5 residues or more) we have considered equally charged sequences as CCXCC or CXCCCC where C means Charged and X is any not charged amino acid. This in consideration of the fact that charge effect extends over the next amino acids.

### Disorder prediction

The prediction of disordered regions in the protein sequences was made by ANCHOR server based on the assumption that disordered proteins have a specific amino acid composition that does not allow the formation of a stable well-defined structure [32] and by DISOPRED2 [33]. Predictions were given as consensus of the two predictors.

### Analysis of Nuclear export and Nuclear localization signals and phosphorylation sites

We have used the NetNES and PredictNLS Servers to predict in all Sirtuin sequences the leucine-rich nuclear export signals (NES) [34,35] and nuclear localization signals by according to the algorithm of Murat Cokal [36], respectively.

The phosphorylation sites have been predicted for all SIRTs from different organisms according to NetPhos Server [37] and DISPHOS [38]. We used also GPS software [39,40] a group-based phosphorylation site predicting and scoring platform, to predict the phosphorylation site (P-site) for human Sirtuins as well as human kinases involved in these phosphorylation.

### GC content analysis

GC content analysis of Sirtuin genes were performed in the various organisms using a script implemented by us (Table 1). This analysis was performed to analyze the effects of the evolutionary pressure on sirtuins. From an evolutionary point of view we considered each protein as organized in three structural parts, i.e. N terminal, C terminal and catalytic Core. We also evaluated the local GC component based on genetic codon’s position. A comparative analysis was also performed between the overall GC content of the protein coding region and the GC content present in the chromosomal bands comprising the protein coding region [41] .

**Table 1:** List of analyzed Sirtuins

**Organisms**	**Class**	**SIRT1**	**SIRT2**	**SIRT3**	**SIRT4**	**SIRT5**	**SIRT6**	**SIRT7**
Chlamydomonas reinhardtii	Plant						**x**	
Ostreococcus lucimarinus	Plant		x					
Micromonas pusilla	Plant		x					
Ostreococcus Tauri	Plant		x					
Arabidopsis thaliana	Plant				x		x	
Triticum aestivum	Plant						x	
Zea Mays	Plant						x	
Oriza sativa	Plant				x	x	x	
vitis vinifera	Plant						x	
Ricinus communis	Plant						x	
Physcomitrella patens	Plant				x	x	x	x
Ajellomyces dermatitidis	Fungi	x	x					
Trichoplax adhaerens	Fungi		x					
Hydra magnipapillata	Cnidaria	x						
Nematostella Vectensis	Cnidaria		x					
Branchiostoma floridae	Cephalochordata		x					
Ciona intestinalis	Ascidiacea		x	x		x		x
Strongylocentrotus	Echinodermata	x			x		x	
Perkinsus Marinus	Dinoflagellata					x		
Brugia malayi	Secernentea	x	x					
Schistosoma Mansoni	Trematoda	x	x					
Caligus Roger Cressey	Crustaceans		x					
Lepeophtheirus Salmonis	Crustaceans		x				x	
Acyrthosiphon Pisum	Insecta				x			
Tribolium Castaneum	Insecta	x						
Apis Mellifera	Insecta	x	x		x	x	x	x
Bombyx mori	Insecta		x					
Drosophila melanogaster	Insecta		x		x		x	
Nasonia vetripennis	Insecta					x		
Pediculus humanus corporis	Insecta	x	x					
Tribolium castaneum	Insecta		x		x			
Anoplopoma Fimbria	Fish					x		
Danio Rerio	Fish		x	x	x	x	x	x
Salmo Salar	Fish		x			x		
Nothobranchius Kuhntae	Fish	x						
Xenopus laevis	Amphibia	x	x	x	x		x	x
Taeniopygia Guttata	Aves	x		x	x	x		
Gallus gallus	Aves	x	x	x	x		x	
Equus Caballus	Mammalia	x	x	x	x			x
Macaca Mulatta	Mammalia	x		x	x	x	x	x
Monodelphis Domestica	Mammalia	x						
Oryctolagus Cuniculus	Mammalia			x				
Pan Troglodytes	Mammalia				x	x	x	x
Ovis Aries	Mammalia				x			
Ornithorhynchus Anatinus	Mammalia	x				x		
Canis Familiaris	Mammailia	x	x	x	x	x	x	x
Bos Taurus	Mammalia	x	x		x	x	x	x
Sus Scrofa	Mammalia	x	x	x	x	x	x	x
Mus Musculus	Mammalia	x	x	x	x	x	x	x
Rattus Norvegicus	Mammalia	x	x	x	x	x	x	x
Human	Mammalia	x	x	x	x	x	x	x

### Nomenclature and compositional mapping of cytogenetic bands

Human genome is composed by five subcomponents, called isochores (L1, L2, H1, H2 and H3). The isochores are compositionally fairly homogeneous regions and are characterized by different average GC levels that increase from L1 to H3. Isochore families L1 and L2 are considered GC-poor families and are characterized by GC% comprised between 34-41%; isochore family H1 is moderately GC-rich and its GC is comprised in the range 41-46% GC; isochore families H2 and H3 are very GC-rich with GC% from 46 to >53. At this point we could ask why the isochores are so important. These isochores are characterized by a different gene density. In fact the genes are not distributed randomly in the human genome: the gene density is low in GC-poor isochores, increases with increasing of GC in H1 and H2 and reaches a maximum in H3, even if this isochore family represent a small amount in the human genome. Because of these properties we called “genome desert” GC-poor isochores and”genome core” GC-rich isochores. Moreover the isochores are correlated with some structural properties (such as intron size, chromatin structure, GC heterogeneity, SINEs, LINEs) and functional properties (such as gene expression, replication timing and recombination). A correlation between the GC-poor and GC-rich isochores with G and R bands, respectively, was proposed by Cuny et al. (1981) [42]. For the low resolution bands (400-band level), we used the definitions of ISCN (1981). For the high-resolution bands (850-band level) we used the idiogram of Francke (1994) [43]. Concerning conventions on chromosomal bands used in this paper, we briefly summarize them here. As shown in Additional file 1: Table S3, at a low (400-band) resolution, G bands (as defined by ISCN 1981) can be subdivided into two lasses of bands, L1^+^ and L1^−^, according to whether they do or do not hybridize single-copy DNA from the GC-poorest L1 family. On the other hand, low (400-band) resolution R bands (as defined by Dutrillaux and Lejeune 1971 [44]) can be subdivided into three classes of bands according to their hybridization of single-copy DNA from the GC-richest isochore families H2–H3: we called H^3^+, H3*, and H3^−^ the R bands that showed a strong, a weak, and no hybridization, respectively [45]. At the high resolution of 850 bands, G bands can be subdivided in L1^+^ and L1^−^ bands according to the same criterion used for low-resolution bands (see above). Likewise, at this resolution, R bands could be classified into just two sets: those that hybridized to H3 DNA, the H3+ bands; and those that did not, the H3− bands [46].

## Results

### Multiple alignment of sirtuin family

**Figure 1:**
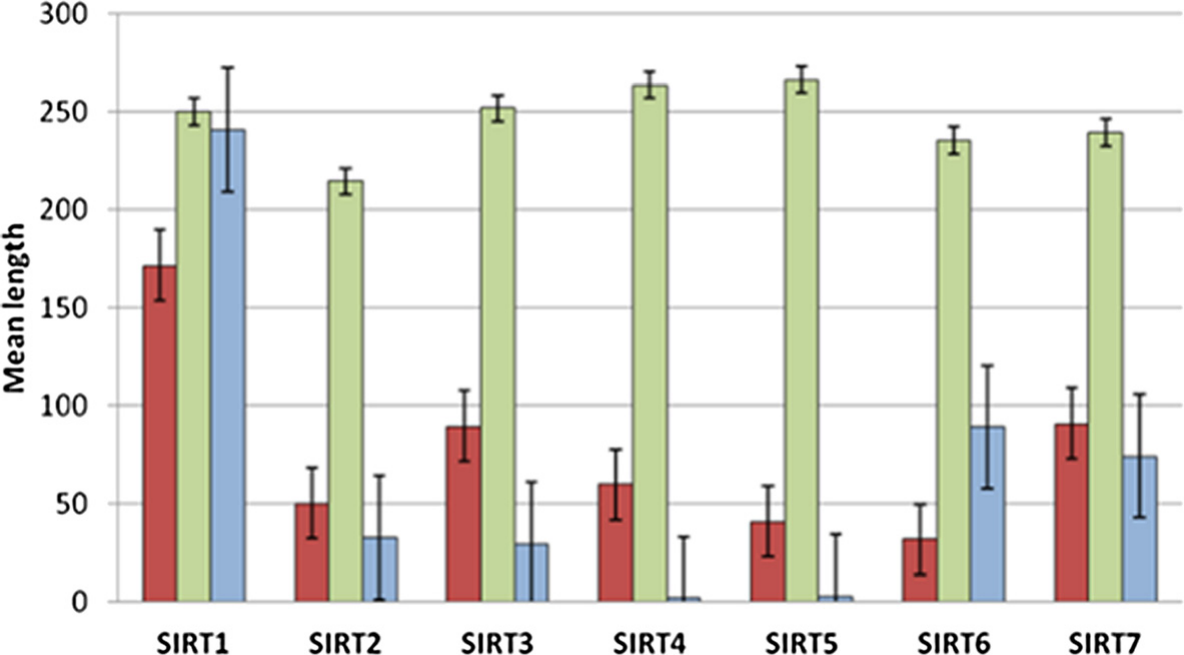
**Mean length of the three regions in the family of sirtuins.** Bars for N-terminal, catalytic core and C-terminal regions are reported in Red, Green and blue, respectively.

Table 1 shows the 53 animal and plant species on which we conducted our analyses. For each organism are indicated the known sequences of the various members of the sirtuin family from 1 to 7. A multiple alignment of the 150 sirtuin sequences was obtained using the program CLUSTALW (Additional file 2: Table S1). A first observation is that the length of the catalytic core is almost unchanged in all the proteins while the lengths of the terminal regions are different in various ways. This is clearly shown in Figure 1 where the average values for all the Sirtuins are reported. In fact, the mean length of N-terminal regions ranges from 31 to 170 residues while that of the C-terminal regions from 2 to 240. In particular, we have found that terminal regions of the SIRT1s are the longest among Sirtuins while SIRT3s and SIRT7s have the N-terminal regions of similar length while SIRT6s present the shortest N-terminal region. The C-terminal regions in SIRT4s and SIRT5s are very shorts with an average value of residues length very small. We have also analyzed in details the length of these terminal regions from an evolutionary point of view (see Additional file 2: Table S1). As one can see, in the population of the SIRT1s we find an increase of the N-terminal region lengths during the evolution until to the vertebrata, where these regions remain almost constant while the C-terminal region length is variable in the most ancient organisms and remains constant only in the vertebrata. In SIRT7s both regions show very similar length in all the examined organisms while in SIRT2s these segments were highly variable during the evolution. In SIRT3s the length of the N-terminal regions is not conserved during the evolution while the C-terminal regions are enough short in almost all of the organisms, except for *Ciona*. In SIRT4s the C-terminal regions remain almost constant during the evolution whereas the N-terminal regions in plants are longer respect to all other organisms. SIRT5s showed N-terminal regions very shorts in the most ancient organisms, except for *Oryza sativa*, but longer and of constant length in the vertebrata, except for *Taeniopygia guttata* and *Ornithorhynchus Anatinus.* The C-terminal regions are very short in all organisms, except for *Anoplopoma Fimbria*. In SIRT6s the N-terminal regions are short and remain constant during the evolution whereas the C-terminal regions in plants are longer respect to all other organisms. These results show that the N- and C-terminal regions have evolutionary histories different from the catalytic core and probably linked to specific and different functions. All these differences suggest a more detailed analysis of their physico-chemical properties as their involvement in multiple molecular recognition should be evidenced by appropriate structural features.

### Primary structure analysis and disorder

**Figure 2:**
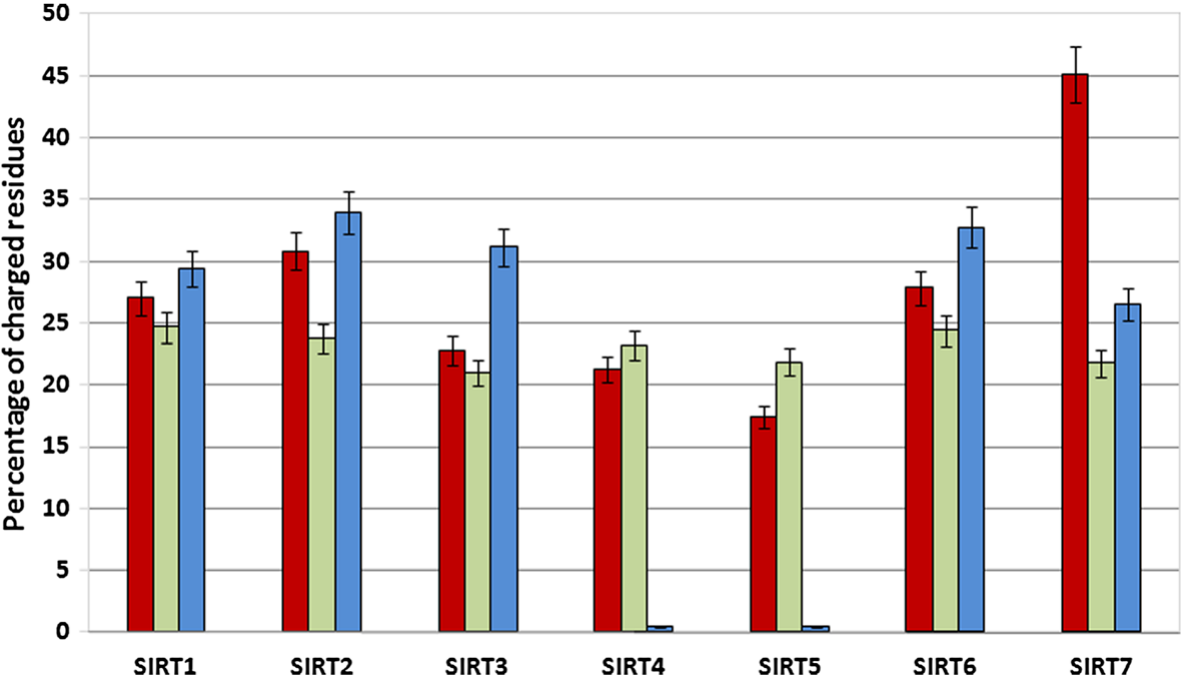
**Analysis of charged residue presence.** We report the average percentages of charged residues in sirtuin families. Bars for N-terminal, catalytic core and C-terminal regions are reported in Red, Green and blue, respectively.

**Figure 3:**
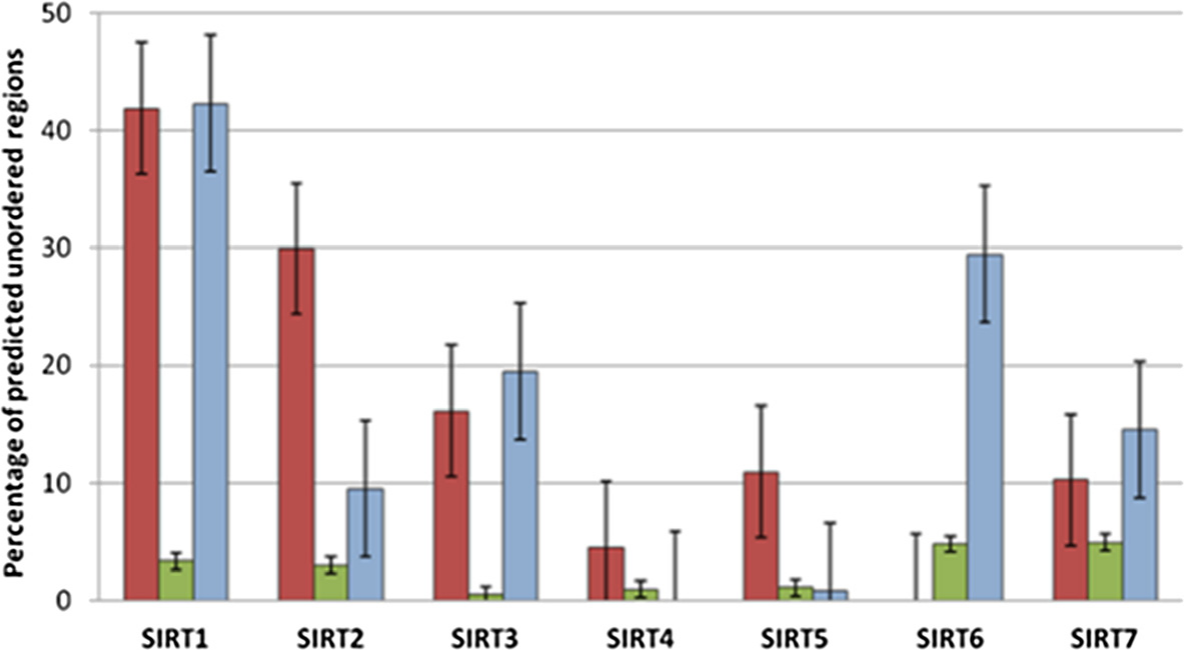
**Results of disordered predictions.** We report the percentage of predicted disordered regions in sirtuins. Bars for N-terminal, catalytic core and C-terminal regions are reported in Red, Green and blue, respectively.

The amino acid composition of disordered proteins is peculiar because poor of non-polar residues [47-49] (Additional file 3: Table S2). This structural feature has been repeatedly shown to belong to proteins that are in the same time poor in hydrophobic residues and, therefore, unable to fold. We have evaluated the average percentage of charged residues (i.e. Lys, Arg, Asp, Glu) present in the N- and C-terminal regions and in the catalytic cores (Figure 2). The N- and C-terminal regions of SIRT1s, SIRT2s, SIRT3s, SIRT6s and SIRT7s have higher percentages of charged residues than those of the globular catalytic cores. In SIRT4s and SIRT5s the percentage of charged residues of the terminal regions is lower respect to that of the catalytic core. This is also due to the short length of C-terminal regions in these two Sirtuins (Figure 2). In particular, we have also observed that the percentages of Cys residues in the terminal regions of all Sirtuin sequences are lower than those in the catalytic core except for the N-terminal regions of SIRT5s and SIRT6s (data not shown). Moreover, we have analyzed the terminal regions of the seven Sirtuins for the presence of charged stretches (Additional file 2: Table S1, C and D). The charged stretches are important because dynamically favor the binding in the molecular recognition. We have considered only stretches starting from three residues and stretches which were evolutionarily significant. As far as the N-terminal regions are concerned, there are three negatively and two positively charged stretches in SIRT1s, two negatively charged stretches in SIRT2s, one positively charged stretch in both SIRT3s and SIRT6s, and five positively charged stretches in SIRT7s while charged stretches are completely absent in SIRT4s and SIRT5s. For the C-terminal regions there are three negatively charged stretches in SIRT1, one positively charged stretch in both SIRT6 and 7 while in the other SIRTs no charged stretch are present. All these structural features strongly support the idea that these proteins can possess segments or regions of disordered structure because the charged stretches have often been found involved in that structural organization which surrounds the phosphorylation sites in the disordered protein segments [48,49]. Moreover, we predicted the disorder propensity in all the sequences belonging to the Sirtuins and evaluated the percentage of disordered regions in the terminal regions and catalytic sites in each Sirtuin group. This analysis is also supported by the fact that our model of the human SIRT1 contains disordered structure [21]. Figure 3 and Additional file 2: Table S1, panel A and B show that in all the seven groups, while the disorder propensity is always higher in terminal regions, it is quite missing in catalytic cores. As far as the N-terminal region is concerned, the SIRTs present different disorder propensity as follows: SIRT1s > SIRT2s > SIRT3s > SIRT5s = SIRT7s > SIRT4s. Even in the case of the C-terminal region the disorder propensity is different: SIRT1s > SIRT6s > SIRT3s > SIRT7s > SIRT2s > SIRT5s. The N-terminal region of SIRT6s as well as the C-terminal region of SIRT4s is very short in all the organisms and thus the disorder is missing. Therefore, these results highlight that members of SIRT1 family have the highest disorder propensity compared to other families in both the regions, N-terminal and C-terminal. Moreover, they present also the longest terminal regions (see Figure 2). This explain why we have recently found by interactomic analysis that the human SIRT1 has such a large number of molecular partners than the other members of the family. In fact, a greater physical length of the sequence increases the probability of dynamic interaction with a partner as well as a better regulation of this property and this suggests also a more fine regulation role for SIRT1s respect to that of other SIRTs particularly in the vertebrata.

### Phosphorylation sites

Recent papers have shown the importance in human SIRT1 of various phosphorylation sites that were mainly found in the terminal regions [15,23-27]. These authors suggested that these regions seem to regulate the human SIRT1 enzymatic activity but little is known of other human sirtuins and even less for sirtuins in general. Protein phosphorylation is very common in eukaryotes and is known to influence protein function by changing protein-protein binding properties, structural stability and spatial organization [50]. It is also involved in signal transduction cascade as well as in the control of the fine-tuning of protein complex assembly. As matter of fact the phosphorylation inserts a strong negative charge into the structure, in particular two negative charges at physiological pH. This charge can also modulate the effect of positive or negative charges when they are located nearby. Considering the presence of the numerous charged sequence stretches, it is conceivable that one of the effects of phosphorylation is to modulate permanently or in temporary way their intensity. Therefore, we evaluated the presence of phosphorylation sites as well as their closeness to charged stretches. For human SIRT1 some sites have already been experimentally evidenced [15,23-27], however we predicted all the putative phosphorylation sites in Sirtuins by NetPhos Server [37] (Additional file 2: Table S1, panel C and D). Concerning the multiple alignments of SIRT1s, five phosphorylation sites are near to three negative stretches in the N-terminal region and two sites near to a negative stretch in the C-terminal region. In N-terminal regions of SIRT2s and SIRT3s three and two putative phosphorylation sites are near to a negative stretch and a positive stretch, respectively. In SIRT6s no phosphorylation site was found near to charged stretches in both N- and C-terminal regions. The SIRT7s present one phosphorylation site near to a positive stretch in the N-terminal region and two phosphorylation sites near to positive stretches in the C-terminal region. Because these phosphorylation sites are fairly well maintained during the evolution, we think that the terminal regions of Sirtuins may reasonably be involved both in regulating functional activities and in the formation of protein multi-complexes. However, the significant enrichment in disorder-promoting residues surrounding phospho-sites in sirtuins strongly support the observation that protein phosphorylation predominantly occurs within intrinsically disordered protein regions [50].

### NetNES and NLS analysis

Eukaryotic cells are highly compartmentalized in particular between cytoplasm and nucleus by the nuclear envelope [51,52]. This envelope contains nuclear pore complexes (NPCs), which mediate the traffic of molecules between the two compartments. The traffic of macromolecules through this barrier is regulated by specific nuclear import and export systems. Proteins that contain Nuclear Localization Signals (NLSs) are imported into the nucleus by a family of specific proteins which bind to NLS containing proteins forming a cargo complex, followed by the translocation through the NPC [53]. Contrary to this, it exists Nuclear Export Signals (NES) generally made by a leucine/isoleucine-rich sequence [52] with a pattern like L-x(2,3)- [LIVFM]-x(2,3)-L-x- [LI], where L can either be L, I, V, F or M, but sometimes known NES regions do not conform to these limitations [53] and the spacing between the hydrophobic residues is variable and NES regions can also be rich in glutamate, aspartate and serine [52]. Several ways of regulating NES-dependent export have been reported, including masking or unmasking the NES and post-translational modifications of the NES-containing protein [54]. In order to identify whether Sirtuins contain a similar sequence, we first used the NetNES prediction method [52,53]. From this analysis (Additional file 2: Table S1, A and B), the N-terminal regions of all SIRTs were predicted to contain NES domains. No signal was found in the C-terminal regions. In the N-terminal region of human SIRT1, its putative NES region is located from residue 210 to residue 220. The output score is consistent with a good probability to have a NES signal. The NLS signal, characterized by the sequence RKKRKD [54] has been found only in the N-terminal region of SIRT1s. This is in agreement with a cytoplasmatic and nuclear presence of this protein. In fact, experimental data suggest a shuttling activity of SIRT1s between cytoplasm and nucleus as recently reported [55]. As stated before, no NLS or NES signals have been found in the C-terminal regions of Sirtuins but the C-terminal regions of SIRT5s and SIRT7s of c*iona* show the presence of the NES sequence. This is interesting because only genes for SIRT 2, 3, 5 and 7 have been found for *ciona* and only on SIRT3 we found a NES sequence. Table 2 summarizes the nuclear export and localization signals found in Sirtuins. Our results also shows that the NES sequences of Sirtuins have slightly different sequences probably reflecting their different functional profiles, and that the divergent amino acids are likely to be critical for the NES activity.

**Table 2:** Nuclear export and localization signals in Sirtuin family

**PROTEIN**	**SIRT1**	**SIRT2**	**SIRT3**	**SIRT4**	**SIRT5**	**SIRT6**	**SIRT7**
**N-terminal**	**NES**	**NES**	**NES**	**NES**	**NES**	**NES**	**NES**
	**NLS**						
**C-terminal**					**NES ( *ciona *)**		**NES ( *ciona *)**

NES or NLS indicates the presence of the signal in a specific group of Sirtuins. No NLS signal has been found in the C-terminal segments.

### GC content Analysis compared to holistic view of the corresponding bands on chromosome

Our analyses of the structural properties evidenced in the Sirtuin family support the idea that cores and terminal arms most likely have different evolutionary histories because they have different structural organizations in the various species, therefore, we have analyzed the GC content in genes of all Sirtuins. GC content is known to vary greatly between different genomic regions in many eukaryotes [56]. Mechanisms previously proposed to explain this variation include selection, mutational bias and biased recombination-associated DNA repair [56]. However, we are not interested to mechanisms but we assume that variations in the GC content are related to gene evolution and the rate of this change among genes in a protein family is indicative of protein compositional changes and thus of evolution. In our study, we have evaluated i) if there is a difference in GC content between globularly structured proteins and disordered proteins such as Sirtuins and ii) if there is a correlation between the GC content of SIRT families and their molecular evolution. In details, we have evaluated the GC content in sirtuins, myoglobins and alpha-crystallins and plotted the value of GC content of each protein on the y axis and the various organisms in phylogenetic order on the x axis in order to assess the performance of GC during molecular evolution. Also the linear regression equations were calculated and compared on the basis of their angular coefficients. We have chosen to compare sirtuin sequences with those of myoglobins and alpha-crystallins; firstly because these are well structured globular proteins. In fact, myoglobins and alpha-crystallins are composed by “alpha-helices” (all-alpha class) and “beta-strands” (all-beta class), respectively. Secondly because their phylogenetic evolution is quite slow. In fact, the low angular coefficients we found for myoglobins (85 sequences) and alpha crystallins (57 sequences, from plants to mammalia) reflects well their slow evolutionary rate. These proteins are characterized by low observed percentage difference of their sequences [57] and, in particular, the calculated slope values of 1.223 and 0.290 (Table 3) correlate well with the difference between their evolutionary distances of 5.0 and 1.5 PAMs, calculated for A and B chains, respectively [57].

**Table 3:** Linear regression equations calculated plotting the value of GC content in each protein family, i.e., SIRTs, myoglobins and alpha-crystallins (a and b chains) on the y axis and the various organisms in phylogenetic order on X axis

	**Full sequence**	**Catalytic core**	**N-terminal**	**C-terminal**
**SIRT1s**	y = 0.898x + 36.77	y = 0.310x + 37.89	y = 1.915x + 34.01	y = 0.520x + 37.62
**SIRT2s**	y = 0.832x + 38.50	y = 0.888x + 37.60	y = 0.957x + 38.64	y = 1.336x + 30.96
**SIRT3s**	y = 0.529x + 54.32	y = 0.879x + 49.96	y = 1.316x + 49.76	y = 1.278x + 43.10
**SIRT4s**	y = 0.933x + 43.09	y = 1.016x + 42.07	y = 0.402x + 48.88	y = 0.389x - 0.317
**SIRT5s**	y = 0.926x + 44.12	y = 0.947x + 44.11	y = 0.830x + 44.55	y = −0.435x + 6.341
**SIRT6s**	y = 2.221x + 36.85	y = 1.766x + 40.43	y = 1.912x + 44.92	y = 3.389x + 30.43
**SIRT7s**	y = 3.841x + 30.92	y = 2.053x + 42.16	y = 2.939x + 45.57	y = 2.177x + 42.60
**Myoglobins**	y = 0,247x + 47,89			
**Alpha Crystallins**				
**chain A**	y = 1.223x + 47.12			
**chain B**	y = 0.290x + 52.71			

**Figure 4:**
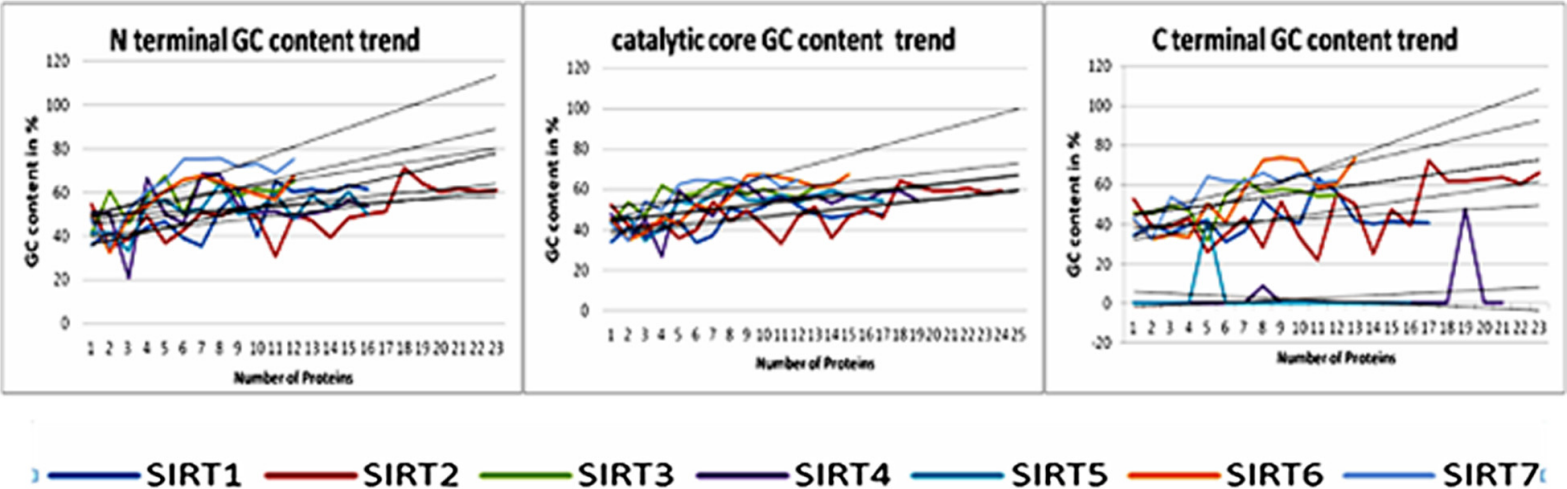
**GC content trends.** We report the trends in the sirtuin family from evolutionary lower organisms to higher and their slopes (black) according to their N-terminal, C-terminal and Core basis content. We have numbered the various organisms according to the sequence shown in Table 1. The colors used for each sirtuin family are reported in the legend.

Moreover, GC content evaluation obtained by the full sequences of the seven Sirtuins across the species shows an increasing trend from the oldest organisms to more recent ones as, for example, humans and other mammalians (Figure 4). This behavior is common to all the seven SIRTs as suggested also from the angular coefficients reported in Table 3. In general, each family shows its characteristic evolution, different from the others. This means that the different cellular compartments in which they operate have exerted an evolutionary pressure on the structure to adapt it to the functional requirements. However, the angular coefficient values indicate that Sirt3 is the only family for which the GC content increased slightly (angular coefficient is 0.529). This might depend on the fact that SIRT3s represent the most recent family. At this point, to have a deeper insight into the evolutionary history of cores and terminal segments, we have analyzed separately the terminal regions from those corresponding to catalytic cores. Our analysis shows that the GC content increases significantly in the terminal regions of all the families except for the terminal regions of Sirt4s and SIRT5s. In the case of core regions, only in SIRT1 family we observe a slight increase of GC content quite comparable to myoglobin and crystalline families. This indicates that the catalytic core of SIRT1s is conserved at structural as well as functional level during the molecular evolution of these proteins when compared to the terminal regions, which, changing more quickly over time, have probably acquired different functions. This agrees well with the predictions of disorder propensity that have evidenced that the terminal regions of SIRT1s have the highest presence of disordered regions compared to the other six families (as reported in Figure 3).

### Comparative analysis on sirtuin genes and chromosomal bands

**Figure 5:**
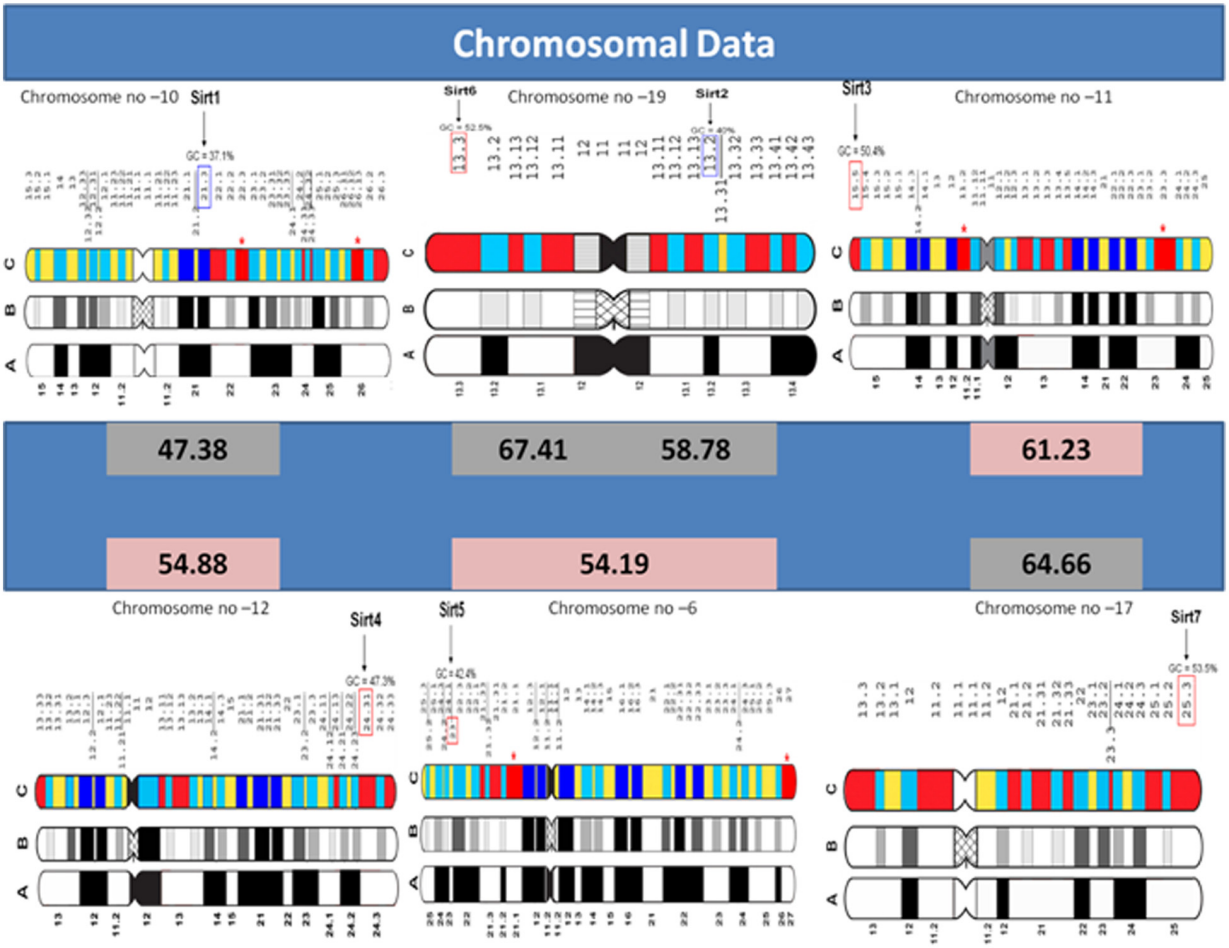
**Chromosomal Data.** We report low (400-band; ideogram A) and high (850-band; ideogram B-C) resolution bands of human chromosome 10, 19, 11, 12, 6 and 11. The correspondence between low- and high resolution bands is unequivocally established in the literature (see Materials and methods). Panel b is the ideogram of Francke (1994). In the high-resolution bands of c, dark blue and light blue bands correspond to L1^+^ and L1^−^ G bands, red and yellow bands to H3^+^ and H3^−^ R bands, respectively. With rectangle in blu or in red are identified the chromosomal bands in which the sirtuin genes are localized. In the central blue rectangle the GC% of sirtuin genes are reported.

It has been reported in literature that when a number of coding sequences were localized and their GC levels were plotted against the GC levels of the large DNA fragments that harbored them, linear correlations were found [58]. Taking advantages of this knowledge jointly to the molecular basis of the classical Giemsa and Reverse bands in human chromosomes [58], it was of interest to see whether there was a possible correlation between GC content of sirtuin genes and the chromosomal bands that harbored them. In more details, we localized sirtuin genes on chromosomal bands at 400-band (or low; see ideograms A) resolution and at 850-band (or high; see ideograms B and C) resolution. In order to clearly explain our procedure we explain, as an example, panel 1 of Figure 5 reporting the chromosomal banding at low- (ideogram A) and high-resolution (ideograms B-C) of chromosome 10. On this chromosome in chromosomal band q21.3 at high resolution having 37.1% GC, SIRT1 is localized (GC = 47.4%, see in the blue rectangle). If we see in more details all the sirtuin genes, we can see that i) SIRT2 is very GC-rich gene (GC = 58.8%) and it is in a GC-poor band (q13.2 on chromosome 19), ii) SIRT6, SIRT3, SIRT4 and SIRT7 are GC-rich genes and they are localized in H3^+^, very GC-rich, chromosomal bands and iii) SIRT5 is GC-rich genes and is localized in H3^-^ band p23 of chromosome 6. In conclusion there is not a correspondence between gene GC% and chromosomal GC% in SIRT1 and SIRT2. An explanation for this last observation seems consistent with the thermodynamic stability hypothesis of GC content in human genome [57]. Costantini M., et al., noted that human genes located in GC-rich isochores show an increased level of GC-rich codons which preferentially encode amino acids that confer stability or thermal stability to the corresponding proteins. Therefore, high content of Lysine, Asparagine, Isoleucine, Alanine, Arginine, Glycine and Proline according to Bernardi [57] are linked to lower protein stability in human genome. If we look at Additional file 3: Table S2 where there is reported the average frequency of amino acids, calculated by DisProt and PDB25 datasets composed by disordered and ordered proteins [49], we can note that amino acids which confer lower thermodynamic stability to the structural organization of proteins are exactly those expected to be higher in intrinsically disordered proteins. Thus, we calculated the total percentage of intrinsic disorder in human SIRTs finding that SIRT1 and SIRT2 show the higher values (70% and 37 %, respectively). This correlates well with the observation that GC for SIRT1 and SIRT2, being the most disordered of sirtuins and thus with poor globular organization compared to the total amino acid content, are in GC-poor bands. This view might reconcile previous contrasting findings about the problem of genome GC content, adding some theoretical background to recent evidences considering that the intrinsically disordered proteins are a conspicuous part of human proteome. Instead, it is known for a long time that proteins to acquire thermostability do not require special amino acid composition.

### Revised phylogenetic analysis of sirtuins

**Figure 6:**
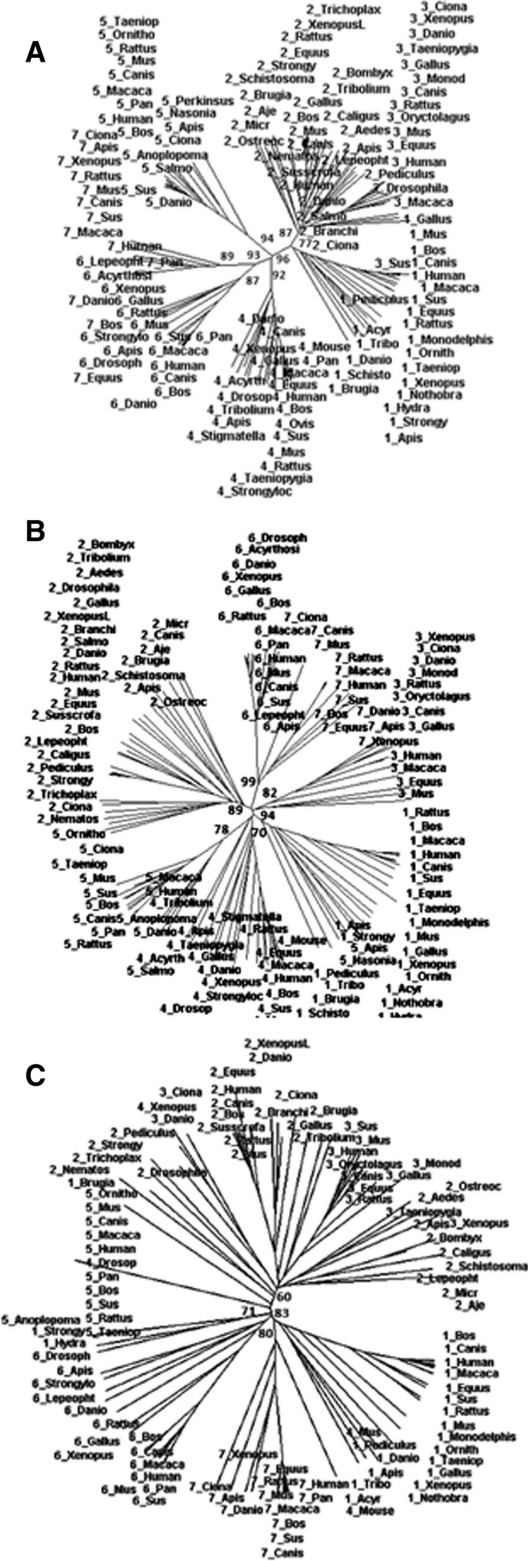
**Phylogenetic analysis.** We report the trees obtained for full-length sirtuin sequences (**A**), N-terminal (**B**) and C- terminal (**C**) regions with bootstrap values evaluated by SplitsTree 4.

Up to this point, our observations strongly suggest that cores and terminal arms have had different evolutionary history. Thus we carried out a phylogenetic analysis on the entire population of sirtuin sequences. At first we considered the whole sequences of the proteins. Table 1 shows the list of organisms and their Sirtuins. The results reported in Figure 6 (A, B, and C) show that the Sirtuins are arranged into four different classes confirming the general framework found by Frye [1]. Class I is divided in two sub-classes where SIRT1 proteins reside in Class Ia but SIRT2 and SIRT3 proteins are in Class Ib. SIRT4 and SIRT5 proteins are part of Class II and III, respectively. Class IV contains SIRT6 and SIRT7 proteins in two different sub-classes IVa and IVb. All these proteins are characterized by a catalytic structural organization that is located in the central highly conserved region where a well-organized Rossman fold is involved into NAD binding. From this evolutionary analysis it seems that the terminal regions contribute in the same way of the catalytic regions with no explicit feature. Considering that our data show that the two terminal regions are often disordered and thus structurally and functionally different from the central region and that this feature is generally enough diffuse in these proteins, we have performed a phylogenetic analysis aimed to understand the evolutionary role of these terminal regions. Therefore, the analysis was repeated on N- and C- terminal regions by themselves. As far as the N-terminal region is concerned, the class IV composed by SIRT6s and SIRT7s is conserved, while SIRT4s and SIRT5s resulted grouped in only one class. Instead SIRT1s, SIRT2s and SIRT3s formed three new different classes (Table 4, panels A, B and C). The phylogenetic analysis of the C-terminal regions shows the presence of only two classes: the first composed by SIRT1s, SIRT6s and SIRT7s, and the second one by SIRT2s and SIRT3s. SIRT4s and SIRT5s are not present because the C-terminal regions in these proteins are very short (Table 4C). On the whole, these results show that the phylogenetic classification made on the basis of the entire sequences does not fit the ones which consider separately the two terminal regions. Evidently other features of these proteins not related to the catalytic properties have had different evolutionary histories. Another important consideration is that the structural and/or functional features of these terminal regions of Sirtuins, that resulted disordered in human SIRT1, are very ancient because already present in more primitive organisms. This opens a gleam on the metabolic controls exerted by the hub proteins during the evolution.

**Table 4:** Sirtuin classes with reference to all the entire sequences (A), N-terminal (B) and C-terminal (C) regions

**A**					
	**Class I**	**Class II**	**Class III**	**Class IV**	
SIRT1	x				
SIRT2	x				
SIRT3	x				
SIRT4		x			
SIRT5			x		
SIRT6				x	
SIRT7				x	
**B**					
	**Class N-I**	**Class N-II**	**Class N-III**	**Class N-IV**	**Class N-V**
SIRT1	x				
SIRT2		x			
SIRT3			x		
SIRT4					x
SIRT5					x
SIRT6				x	
SIRT7				x	
**C**					
	**Class C-1**	**Class C-II**			
SIRT1	x				
SIRT2		x			
SIRT3		x			
SIRT6	x				
SIRT7	x				

### Putative phosphorylation sites of human Sirtuins (Group based prediction System)

The presence of numerous phosphorylation sites (P-sites) on the human sirtuins suggests a leading role of kinases in the recognition of molecular partners as well as in the metabolic info-signaling. What could be important at this point is to know which family of human kinases is involved into human sirtuins phosphorylation because from this knowledge we can try to understand their cellular localization and/or the metabolic occurrence of their biological action. This is a critical point. We have recently demonstrated by interactomic analysis [59], that the family of human sirtuins interacts with about 25% of the human proteome up to now known. They have a clear role as hub protein due to the presence of disordered structure as well as the large number of putative phosphorylation sites and human SIRT1 has been found to be able of interacting with more than 130 different human proteins in various cellular compartments, including the nucleus. To our knowledge, this is a very high number of molecular partners, perhaps one of the highest if not the greatest ever. This opens an important perspective on the role of these proteins which, as demonstrated by the literature, is already vast, and also on the molecular mechanisms that underlie the variety of biological activities they control. Since the information on the molecular mechanisms of these proteins is very poor because we still have a poor structural knowledge, we might have additional information from the type of kinases. Kinases potentially show phosphorylation sites on each human sirtuin by group based prediction system. The results were reported in Table 5 where the comparison with other two softwares is also present: NetPhos [37] and Phosphosite [60] (database of phosphorylation sites). Results, obtained with high stringency level, showed that P-sites are much more numerous and concentrated in the N- and C-terminus only for SIRT1, while for the other six Sirtuins, P-sites are similarly diffuse also in cores. In particular, for SIRT-1, GPS predicts 20 P-sites in the N-terminus, 10 in the Core and 50 in the C-terminus; for SIRT-2, 8 P-sites in the N-terminus, 28 in the Core and 09 in the C-terminus; for SIRT-3, 11 P-sites in N-terminus, 17 in Core and 3 in C-terminus; for SIRT-4, 5 P-sites in N-terminus, 23 in Core and 0 in C-terminus; for SIRT-5, 5 P-sites in N-terminus, 21 in Core and 1 in C-terminus; for SIRT-6, 4 P-sites in N-terminus, 25 in Core and 9 in C-terminus; for SIRT-7, 6 P-sites in N-terminus, 31 in Core and 11 in C-terminus. However, the majority of Phosphorylation sites predicted with a higher score by the prediction algorithm is present on N-terminal and C-terminal regions. SIRT 4 and SIRT 5, which are relatively shorter or lacks N- or C- terminal regions, were also predicted to be phosphorylated, but by a reduced number of kinases. In Addition, cores show scarcely distributed phosphorylation sites which support the correlation between phosphorylation and structurally disordered terminal regions of sirtuins. In Table 6 we report the Human kinases which are predicted to phosphorylate specific residues in human sirtuins acting on N-terminal residues, cores and C-terminal. The table also reports phospho-sites predicted but already experimentally determined. Moreover, in the table are also reported known functions and cellular localization of 23 predicted human kinases. As one can see, these kinases are widespread in almost all cellular compartments with a high number of functional involvements. This means that human sirtuins too should be involved in the same cellular sites. If we put together all the pieces of the human sirtuins puzzle, that is, the great number of metabolic functions in which they are involved, the large number of molecular partners that some of them possess, the large number of phosphorylation sites present on their structure, the large number of different kinases, the large number of cellular sites, we have a coherent framework in delineating their role as metabolic node (or hub protein) which probably is the most important property possessed by them. In details, MAPK8 phosphorylates human SIRT1 at residue 27 supporting correctly the prediction because serine 27 is experimentally known to be phosphorylated [61]. MAPK8 predicted residue 530, 540 and 545 with a score similar to that of serine 27. MAPK8 is also predicted to phosphorylate SIRT2, 4 and 6, at residue 368, 36 and 338, respectively, with a very high score. SIRT4, which is a mitochondrial protein, during its transportation across membranes from its site of synthesis in the cytoplasm, might interact with cytoplasmatic as well as nuclear MAPK8 as supported by the presence of a transit peptide in the first 28 residues. Similarly, SIRT5, which have a transit peptide too shows a phosphorylation activity by means of MAPK11 (nuclear/cytoplasmic localized kinase). Map2k2, the activity of which has been detected in various cellular sites, including the mitochondrial one, also acts on SIRT3 at residue 331 with high score.

**Table 5:** Phosphorylation sites predictions for human sirtuins

	**NetPhos**			**Group based prediction system (GPS)**			**Phosphosite**		
	**N-term**	**Core**	**C-term**	**N-term**	**Core**	**C-term**	**N-term**	**Core**	**C-term**
**SIRT1**	13(27%)	5(10%)	30(63%)	20(25%)	10(13%)	50(62%)	8(38%)	1(5%)	12(57%)
**SIRT2**	5(22%)	11(48%)	7(30%)	8(18%)	28(62%)	9(20%)	3(33%)	2(22%)	4(45%)
**SIRT3**	8(50%)	7(44%)	1(6%)	11(35%)	17(55%)	3(10%)	0(0%)	2(100%)	0(0%)
**SIRT4**	1(11%)	8(89%)	0(0%)	5(18%)	23(82%)	0(0%)	0(0%)	2(100%)	0(0%)
**SIRT5**	4(29%)	10(71%)	0(0%)	5(19%)	21(78%)	1(03%)	0(0%)	4(100%)	0(0%)
**SIRT6**	2(11%)	11(58%)	6(31%)	4(11%)	25(66%)	9(23%)	1(13%)	1(13%)	6(74%)
**SIRT7**	5(19%)	16(62%)	5(19%)	6(13%)	31(65%)	11(22%)	0(0%)	0(0%)	0(0%)

We report the prediction obtained by Netphos and GPS. Phosphosite was used as additional reference, though it contains few data about sirtuins. GPS is specific for human sirtuins. In the rows are indicated the total number of phosphosites for segment and in parenthesis the percentage on the total number of phosphosites in the given protein.

**Table 6:** Human kinases which are predicted to phosphorylate specific residues in human sirtuins

**Kinase**	**Function**	**Localization**	**Sirt-1**	**Sirt-2**	**Sirt-3**	**Sirt-4**	**Sirt-5**	**Sirt-6**	**Sirt-7**
**BTK**	**B lymphocyte development, differentiation and signaling.**	**Cytoplasm, Cell membrane, Nucleus.**	121-Y	**104-Y**					
**CDK1**	**Control of the eukaryotic cell cycle.**	**Nucleus, Cytoplasm, Mitochondrion.**	**14-S 27-S 47-S 530-T 540-S** 682-S	218-T 280-T **368-S 372-S**	150-T	36-S	87-T 178-S	**294-T 303-S 330-S 338-S**	345-T
**CDK2**	**Control of the cell cycle**	**Cytoplasm, Cytoskeleton, Centrosome, Nucleus, Cajal body, Cytoplasm, Endosome.**	**14-S 27-S 47-S 530-T 540-S** 545-S 682-S	280-T 365-T **368-S 372-S**				**294-T 303-S 330-S 338-S**	
**CDK4**	**Regulate the cell-cycle during G**_**1**_**/S transition.**	**Cytoplasm, Nucleus, Nuclear Membrane.**	**14-S 27-S 530-T 540-S** 545-S	98-S 279-S **368-S 372-S**				**294-T 303-S 330-S 338-S**	345-T 377-S
**CDK5**	**Neuronal cell cycle arrest and differentiation**	**Nucleus, Cell membrane, Cytoplasm.**	**27-S 530-T 540-S** 545-S	218-T **368-S**				**294-T 303-S 330-S 338-S**	
**CDK6**	**Control of the cell cycle and differentiation**	**Cytoplasm, Nucleus.**	**14-S** 545-S	280-T **372-S**	329-S				
**CDK7**	**Cell cycle control and in RNA polymerase II-mediated RNA transcription.**	**Nucleus, Cytoplasm, Perinuclear region.**	**27-S** 344-T 545-S 549-S 605-S **747-S**	166-T				94-T **294-T** 355-S	148-T 400-T
**DYRK1**	**Regulation of DNA damage response and regulation of protein deacetylation**	**Interchromatin regions of nucleoplasm**	**14-S** 111-S **538-S** 744-S **747-S**	53-S 249-S **372-S**	117-S			**330-S 338-S** 355-S	
**EGFR**	**Activate signaling cascades; involved in cell-cell adhesion, regulation of apoptotic process, regulation of MAPK**	**Cell membrane, Endoplasm, Golgi apparatus membrane Nucleus membrane, Endosome membrane.**	642-Y 699-Y			**74-Y**			
**JAK1**	**Involved in the IFN-alpha/beta/gamma signal pathway.**	**Endomembrane system, Peripheral membrane protein.**	699-Y 742-Y						
**LCK**	**Developing T-cells and function of mature T-cells in thymus**	**Cytoplasm, Cell membrane, Lipid rafts.**	121-Y 658-Y 699-Y 742-Y	**104-Y**	165-Y				
**MAPK3**	**MAPK signal transduction pathway**	**Cytoplasm, Nucleus.**	**27-S 47-S 530-T 540-S**	218-T 280-T **368-S 372-S**		36-S 176-T		**294-T 303-S 330-S 338-S**	345-T
**MAPK8**	**Cell proliferation, differentiation, migration, transformation and programmed death.**	**Cytoplasm, Nucleus.**	**27-S 530-T 540-S** 545-S	**368-S**		36-S		**294-T 330-S 338-S**	
**MAPK9**	**Cell proliferation, differentiation, migration, transformation and programmed death.**	**Cytoplasm, Nucleus.**	**27-S 530-T 540-S** 682-S	280-T **368-S 372-S**				**330-S 338-S**	
**MAPK10**	**Neuronal proliferation, differentiation, migration and programmed death.**	**Cytoplasm, Membrane, Lipid-anchor, Nucleus.**	**14-S 27-S 540-S** 682-S	**368-S 372-S**				**303-S 330-S 338-S**	
**MAPK11**	**MAPK pathway and role in cellular responses to proinflammatory cytokines or physical stress**	**Cytoplasm, Nucleus.**	**540-S** 545-S 682-S	98-S 280-T					
**MAPK12**	**Role in cellular responses to proinflammatory cytokines or physical stress**	**Cytoplasm, Nucleus, Mitochondrion.**	**14-S 27-S 540-S** 545-S	280-T **368-S**	150-T				
**PAK1**	**T cell receptor signaling pathway, apoptotic process, regulation of cell proliferation**	**Cell-extracellular matrix contact, Cytoplasm.**	**615-S**						
**PAK2**	**Cytoskeleton regulation, cell motility, cell cycle progression, apoptosis or proliferation.**	**Nuclues, Cytoplasm, Cell membrane.**	**615-S**						
**PKD1** **(PRKD1)**	**DAG signaling, regulation of MAPK8/JNK1 and Ras signaling, Golgi membrane integrity and trafficking, cell survival through NF-kappa-B activation, cell migration, HDAC7 nuclear export, cell proliferation via MAPK1/3 (ERK1/2)**	**Cytoplasm, Cell membrane, Golgi apparatus.**						152-T 167-T	
**PLK1**	**Cell cycle, centrosome maturation and spindle assembly, removal of cohesins from chromosome arms, anaphase-promoting complex, regulation of mitotic exit and cytokinesis.**	**Nucleus, Kinetochore, Centrosome, Cytoskeleton.**	**538-S 540-S** 550-S	**23-S 25-S** 365-T **368-S** 383-T				**294-T 330-S**	400-T
**PRKCA**	**Organizes subcellular localization of membrane proteins containing PDZ sequence, clustering of receptors, synaptic plasticity.**	**Perinuclear region, Synapse.**		383-T	337-S			229-T	
**ZAP70**	**Regulation of adaptive immune response, motility, adhesion and cytokine expression of mature T-cells, thymocyte development. development and activation of primary B-lymphocytes.**	**Cytoplasm, Cell membrane.**	121-Y 699-Y						

[In red the kinases acting on N-terminal residues, in green on cores and in light blue on C-terminal. Phosphosites predicted but already experimentally determined are indicated underlined and in bold. In the table known functions and cellular locations exerted by these kinases are also reported [Shchemelinin et al., 2006].

## Discussion

Sirtuin genes are very ancient. This is an important aspect to illustrate their history because the proteins derived from them have proved to have many new important functional properties in different cellular environments as well as organisms. We have used basic structural and computational considerations to characterize structurally and phylogenetically the N and C terminal segments of these proteins but, up to now, no one made these same considerations which are certainly important to understand the molecular mechanisms of action of this protein family. In fact, the functional importance of their catalytic and metabolic role in humans is well studied, but little is known about their structure-function relationships that are approached as if sirtuins were entirely globular protein. These proteins perform complex functions that cannot be the result of the catalytic activity alone. Sirtuins, in particular SIRT1s and SIRT3s, interact with numerous molecular partners, many of which are non-histone proteins. Hence, this remarkable propensity for such an ability to interact cannot depend only from the catalytic core. In fact, evolutionarily conserved cores should express only similar functional activity, with few molecular partners, provided that the structures of these partners are evolutionarily conserved too. Instead, these proteins show a great ability to interact with structurally and functionally very different partners and, up to now no structural explanation has been given. In particular, the role of their terminal segments is not yet clear. A direct example is given by the interactome of human sirtuins [59] constructed on the basis of the experimental data produced by Law et al. [62] by using affinity purification and MALDI-TOF/TOF-MS/MS analysis. From the interactome one can see the incredible number of interactions with many different proteins. The interaction with numerous molecular partners requires the availability of many different structural solutions to effectively modulate the recognition. The functional importance of the flexible disordered regions resides in their ability to allow proteins to bind different partners. This aspect is often neglected, even when structural models of specific Sirtuins have been attempted, their terminal regions were never taken into account, however, their regulatory role was suspected [63-65].

Our recent 3D-model of human SIRT1 [21] showed two very long flexible terminal segments with diffuse lack of structural organization and few transient helices, and hence, the presence of disordered regions, a peculiar structural feature, already known in other proteins, but unexpected in Sirtuins. Thus, we have analyzed their evolutionary history to understand how ancient and shared were those disordered segments found in human SIRT1. Our results show that in the terminal regions of Sirtuins there is a diffuse disorder propensity. The SIRT1s present the highest presence of disordered regions both in N- and C-terminal regions, but not in the catalytic site (see Figure 3). Disorder is therefore advantageous because it allows sirtuins to partner with interacting molecules more exclusively than a rigid protein would. This feature can favor the interaction between SIRT1s and their regulatory factors, and highlights that disordered regions facilitate the exposure of charged sequence stretches and interaction points, which are essential for the protein-protein recognition. This also evidences that in the other Sirtuins the functional regulation exerted by the terminal regions seems less crucial than in SIRT1s.

To our knowledge, no other work has been done with regard to the structural and functional effects exerted by the presence of intrinsically disordered regions in Sirtuins of eukaryotes. Accumulating data suggest that these deacetylases acquired new roles as genomic complexity increased, including deacetylation of non-histone proteins and functional diversification in mammals [66].

The Sirtuin family comprises of seven different homologous groups of proteins with different intracellular locations, activities and biological functions. A phylogenetic analysis of their sequences suggested that they could be grouped in four different classes according to Frye [1]. The sequence similarities are well retained among regions of the catalytic core. But the same analysis, repeated only on terminals regions, allows a different grouping that depends on the different structural features of the terminal segments and on the different length of these regions; in fact, SIRT4s and SIRT5s present very short C-terminal regions. We discussed about the possibility that these terminal regions were acquired during the evolution and compared their lengths in relation to the evolution of each organism. Our data show that the examined organisms present Sirtuin sequences that have terminals regions of different length in dependence of the class to which they belong. This highlights that there is a conservation of the length in Sirtuins and suggests that the functional properties acquired during the evolution are very old. The number of Sirtuins varies among different organisms and generally correlates with greater complexity (Table 1). However, the need to support complex functional properties adding new structural features appears to be differently distributed according the cellular location and specific functions. In fact, SIRT1s, SIRT2s and SIRT3s seem to need a consistent structural support from their terminal regions and are characterized by the fact that all have a clear H4K16 deacetylase activity [1] even if SIRT1s and SIRT3s show also a very strong non histone deacetylase activity [8]. The first two have nuclear as well as cytoplasmatic location [5-7] while that of SIRT3s is still under consideration [8-10]. The three mitochondrial Sirtuins, i.e. SIRT3s, SIRT4s and SIRT5s have different functional activities that are H4K16 deacetylase, ADP-ribosilation and non-specific deacetylation, respectively [8-12]. SIRT4s and SIRT5s lack appreciable C-terminal regions and have very few disordered residues in their not so long N-terminal regions, no charged stretch or phosphorylation site (Additional file 2: Table S1). SIRT6s and SIRT7s are reported to have nuclear location [13,14]. In particular, SIRT6s are ADP-ribosilases and deacetylate the histone H3 but they possess in lower extent the same structural characteristics of SIRT1s, SIRT2s and SIRT3s. In general, it is difficult to classify the cellular function of Sirtuins as nuclear (SIRT1s, SIRT2s, SIRT6s and SIRT7s) or mitochondrial (SIRT3s, SIRT4s and SIRT5s) or predominantly cytoplasmatic like SIRT2s. Interesting is also the SIRT1 of *Strongylocentrotus,* (Table 1 and Additional file 2: Table S1) that presents an anomalous very long, flexible, very rich in disordered segments and charged stretches of the C-terminal segment and also the presence of seven similar sequence repeats of exactly 14 identical residues. A search on ExPASy (SIB Bioinformatics Resource Portal) for structural motives revealed the repetitive presence of phospho-site (TTVD) for casein kinase II (consensus pattern: (S/T)-x-x-(D/E)) in all repeats (…GETTVDQRPDPVLDEGETTVDQRPDPVLDE…) and close to a negatively charged stretches (DEGE) [67]. No other site for this kinase has been up to now predicted or experimentally found in sirtuins. Therefore, *Strongylocentrotus* seems to possess this unique structural feature not shared with other sirtuins but close to those of higher organisms. An explanation of why this occurred, it is difficult to give but it is clear that the molecular recognition, mediated by kinases, is a property so evolutionarily important to appear by itself when the functional needs require it. In conclusion, the substantial structural diversification of Sirtuins that has occurred since the divergence of plants, animals and fungi suggests a surprising degree of evolutionary plasticity and functional diversification.

Disorderedness is known to be linked to amino acid sequence [38]. Our results show that there are charged sequence stretches in the flexible terminal regions of Sirtuins suggesting that the function of N-terminal or C-terminal regions should be principally of recognition. In fact, it has been clearly established the existence of interactions between many proteins and a wide variety of polyanionic surfaces within a cell [68]. Proteins possessing this feature are called Polyanion-Binding Proteins (PABPs) [68]. They contain multiple positively charged regions, are involved in phosphorylation processes as well as in protein-protein interaction networks in a functionally significant manner and a substantial number of them are “natively unfolded”. This suggests that the nearness between flexible charged sequence stretches and interacting residues can get better the recognition of Sirtuins with their molecular partners and represent an important support to our hypothesis that N- and C-terminal regions of Sirtuins are essential for their functions. Furthermore, a fine regulation would occur through the involvement of regulation factors, such as exogenous and/or endogenous activators, which reasonably should recognize and interact also with the terminal regions through charged stretches or interaction points. Therefore, the presence/absence of phosphate groups seems important in modulating the recognition of the different proteins and to regulate the enzymatic activity [69]. Most of the sirtuins show numerous phospho-sites on terminal segments. This special condition should be considered taking into account that these segments are intrinsically disordered and therefore very flexible; they have charged stretches close to which or in which the phospho-sites are often allocated. All this leads us to consider that they represent structural regions highly exposed and available to the recognition of molecular partners. The role played by the presence/absence of phosphorylation is well known in numerous molecular mechanisms shown from globular proteins, but acquires a particular significance when the sites are numerous and on the same protein segment provided that is highly mobile. In fact, the presence/absence of negatively charged phosphate localized on more points spatially correlated generates an high number of physically different structures. Therefore, this means that proteins with numerous phosphorylation sites on mobile segments possess a considerable number of possible structural isomers. These simple considerations lead to understand how the structural flexibility may be the driving force for recognition phenomena of a large number of different molecular partners. If we add as possible variables of the molecular recognition also different affinities for each different structural phospho-isomer, that is, the modulation of the interaction strength due to the different affinity determined by the local charge density variation, we have a huge number of isomers dynamically available by the same segment for molecular recognition.

The deacetylation of numerous cytoplasmatic non histone proteins entitle us to believe that under metabolic demanding needs and supported by a shuttle mechanism [70], an ambivalent functional behavior for each Sirtuin is supposable. Hence, we have performed a NES analysis that has shown that various Sirtuins possess this signal in the N-terminal regions and only SIRT5 and SIRT7 of *ciona* in C-terminal region. Since recent phylogenetic analyses provided compelling evidence that tunicates, and not cephalochordates, represent the closest living relatives of vertebrates and *ciona* is a tunicate [71], we suggest that the nuclear export signal is very ancient and that *ciona* SIRT5 and 7 have functions requiring a traslocation into cytoplasm. More controversial is the situation of the NLS pattern characterized by short stretches of positive charge. In fact, the Sirtuins are also rich in sequences of positive consecutive amino acids and their abundance suggests that these regions could be used both to recognize negative stretches present on other macromolecules or to allow the entry of Sirtuins in the nucleus. If one regards the nuclear localization signal and nuclear export signal as competing forces, the Sirtuins 1, 2, 3, 5, and 6, seem to have the right organization to be efficient shuttles. Moreover, the truncation of the N-terminal sequence of certain Sirtuins (e.g., human SIRT1 and SIRT3) recently described in literature [8-12] may be understood as a means to regulate this activity preventing the entrance or escape from the nucleus. Future *in vivo* studies will decipher whether the presence of the "NES" sequence in the Sirtuins has any role during the cellular cycle.

## Conclusions

Sirtuins possess globularly structured cores which are evolutionarily maintained fairly unchanged. These cores are associated with catalytic activities quite similar among species, with a few minor exceptions. In the face of this quite typical catalytic activity, they developed a dynamic ability for the recognition of molecular multi-partner, an ability that becomes increasingly complex with increasing metabolic complexity and supported by disordered flexible segments as phospho-isomers. This framework opens a window on some functional aspects that should reasonably be involved, namely, the need for a synchronous space temporal connection to have at the same time and in the same cellular district the functionally appropriate sirtuin, the right kinase(s) and the proper molecular partner. Indeed, SIRTs are emerging with key roles as HUBs in the metabolic network [59] with tissue specific functions, characterized by a complex interplay of multiple factors. Thus, in principle, sirtuins with a greater number of phosphorylation sites should have a greater number of partners, such as SIRT1, 2 and 6, but still we do not know how many and which sites are necessary for the recognition of specific macromolecules. Based on the foregoing, the most evident aspect about the world of sirtuins, is complexity. An aspect that cannot be tackled with the simple logic of globular proteins, thus, in our opinion, the methods for the assessment of SIRT function *in vitro* should be rethought to obtain functional information as close as possible to their true metabolic role exerted in a specific cellular environment and this can only be done with integrated studies at various functional levels, that is, by an holistic approach.

## Competing interests

The authors have no competing interests to declare.

## Authors’ contributions

SC, GC, AS, RR, IA, and MC performed the computational analysis. SC and GC initiated the design and coordination of the study and wrote the manuscript. All authors read and approved the final manuscript.

## Supplementary Material

Additional file 1: Table S3 Chromosomal bands as defined by isochore hybridization. Click here for file

Additional file 2: Table S1 Sequence Analysis of Amino Terminal Segments (A) and Carboxy terminal segments (B) where the disordered structure predictions are evidenced in yellow, the NES (Nuclear Export signal) in red and NLS (Nuclear Localization Signal) inviolet. Amino Terminal Segments (C) and Carboxy terminal segments (D) where negative sequence stretchs are evidenced in red, positive sequence stretchs in light blue and predicted phosphorylation site in green. Click here for file

Additional file 3: Table S2 Frequency of amino acids in DisProt and PDB25 datasets collecting disordered and ordered protein domains, respectively. Click here for file
