# Supplementary material for: Genealogy of an ancient protein family: the Sirtuins, a family of disordered members
Source: BMC Evol Biol. 2013 Mar 5;13:60. doi: 10.1186/1471-2148-13-60 (PMC3599600; doi:10.1186/1471-2148-13-60)
Supplement: Additional file 1: Table S3 — Chromosomal bands as defined by isochore hybridization. [file 1471-2148-13-60-S1.doc]

**Table S3.** Chromosomal bands as defined by isochore hybridization

|  |  |  |  |
| --- | --- | --- | --- |
| **At 400 band resolution:** |  |  |  |
|  |  |  |  |
|  |  | **L1 hybridization** |  |
| **G bands** | L1^+^ | present |  |
|  | L1^-^ | absent |  |
|  |  | **H3 hybridization** |  |
| **R bands** | H3^+^ | strong |  |
|  | H3^*^ | weak |  |
|  | H3^-^ | absent |  |
|  |  |  |  |
| **At 850 band resolution:** |  |  |  |
|  |  | **L1 hybridization** |  |
| **G bands** | L1^+^ | present |  |
|  | L1^-^ | absent |  |
|  |  |  |  |
|  |  | **H3 hybridization** |  |
| **R bands** | H3^-^ | absent |  |
|  | H3^+^ | present |  |
|  |  |  |  |
