# Supplementary material for: Genealogy of an ancient protein family: the Sirtuins, a family of disordered members
Source: BMC Evol Biol. 2013 Mar 5;13:60. doi: 10.1186/1471-2148-13-60 (PMC3599600; doi:10.1186/1471-2148-13-60)
Supplement: Additional file 2: Table S1 — Sequence Analysis of Amino Terminal Segments (A) and Carboxy terminal segments (B) where the disordered structure predictions are evidenced in yellow, the NES (Nuclear Export signal) in red and NLS (Nuclear Localization Signal) inviolet. Amino Terminal Segments (C) and Carboxy terminal segments (D) where negative sequence stretchs are evidenced in red, positive sequence stretchs in light blue and predicted phosphorylation site in green. [file 1471-2148-13-60-S2.doc]

**Table S1**

**A) SIRTUINS – AMINO TERMINAL SEGMENTS: central core (missing) on the right side**

**a)DISORDERED STRUCTURE IN YELLOW,**

**B)NES (NUCLEAR EXPORT SIGNAL) IN RED,**

**and C)NLS (NUCLEAR LOCALIZATION SIGNAL) IN VIOLET**

**1Hydra_mag --------------------------------------------------------------------------------------------------------------------------------------------------------------MADPIANNSMQLVKD----------EATFSPHDFIKN--PLQWIQNQIENEVD-PR-ILIRQLVPQIK---LPHDVEDSTLWNVIFEIIS------------------------EPSPRKRLSNINSL**

**1Stron_p ---------------------------------------------------------------------------------------------------------EEGHVEQEVSDGPAPSWGSQASSSDSSSSSSSGHQPGGDSSSEGSITDSFDMGLGDIRPDSLAGVLHC----------NHSYLVCDTLPQPGPMGWLQKQMMSGTN-PKSILMRIIPSGMT---IPEEMDEFEMWSIIAEYLRSI---------------------DEPPPRQKLEQYNTF**

**1Brugia_mal ----------------------------------------------------------------------------------------------------------------------------------------MHSGGCGNELISENIVLDGNMGTETDTYASSVTNSSSMG--NSAVQMQGLTDSRDSSADSGDISAVDEVSGESES-ARDVVRCLLPHVN----LPENLCEQDLYRIIQKILYS-----------------------ERPKRTKLSEFNSF**

**1aSchis_ma ---------------------------------------------------------------------------------------------------------------MDTKKEPSISIDNSEIVVLSSDDENDSQNSHDNISNEKLSSSVISIEDDDDNNTNNDESAES---DCEILNVEDLQGEEKWRDIHGPFKRLNRLIQAGFNDPRLLLVRVFGMDE----NSLPSDPNQLLSLLLTLLA------------------------EPAPRRRLRRINSL**

**1Schis_ja ---------------------------------------------------------------------------------------------------------------MDTNKEQSVDVDNSQIVVLSSDDENDSQNSHDNLSNEKGSSSVISVDDDDDN-TNNDESAES---DCEILSVEDLQGEEKWRDIHGPFKRLSSLIQAGFNDPRLLLVRVFGMDE----DSLPSDPNQLLSLLLTLLA------------------------EPAPRRRLRHINSL**

**1Acyrt_pis MASEDNGDFDTDLSAAPAKRMRYSFADEIARTSHFEAFGQRFTGFPSGECYANNLYQQS----------------------------------------------------YKSLDTLS-PNYLQPNSPTPPLTMSSSLPESPEDS----SIFDESSDTKEEPEDDDSSSTSSS---DSSTHSENADSE---KGVPGSMDWVQRQIMGGIN-PRRLLHQVFGAS-----VPSQLEDITLWRIIMSMTD------------------------DSPIRNRLRSVSSL**

**1Trib_cas MDT--YPDVAEHESSAKRIKLDLR-ESNIGELCNGGDFSSYSTSVGGPIDIPEGISPDC-------------------------------------------------DSGYEASTLESIPTSSHQTSPHTDPGDVPESLSSLSEIFLTPSRTDQADTSQDSIEADDDNASTVS---EISGLSDLSGQD--WKPMAGSMIWIQKQMQNGVN-PRTLLSDLGVDL---DQVPQYVDEITLWKLIINMLA------------------------EPPRRNKLRHVNTL**

**1Apis_mel MAS--GSELPEYSSPAKRRKVDGIGYSGTSSQ--KPDF----QDCETSHDIPNEDPEET-------------------------------------------------YGGDSGFNELSDESKSTSISPDA-----TNLMTTPSRI---DSTSDDTGCLIDTADEKDEVSSTVSNLSDLSGLSDFSGEGDINHQWRNASSWVQKQMLIGAD-PRNLLHHLLMDS---TQIPEQVDDLTLWKIIINMMS------------------------EPPRRQKLRHINTL**

**1Pedi_hu_c MAS--NCDVQERTSSVKKQKIECS-SSGTLENNIKMDVNLFSDKFSTSSEILKESVQENRIAPLSTEQ--------------------------------------VLNGGDSGFSEIEMTSSTEVLSENARLQSFENDIQSPVPG---SSTFDSSMEIRDELNDNDDISSTIS---DLSGISDLSGQD--WKPTSGPMSWIHHQMANGAD-PRDLLFQLVQDKAAIAALPSGKDDFTLWKIIISMLS------------------------EPPRRKKLSTINTL**

**1Danio_rer ----------------------------------------------------------------------------------------------------------------------------------------------------------------------------------------------------------------------------------------------------------------------------------------------**

**1aNoth_fu -MADEENSLGTAFSGAFITDEPATKISKMDTPTNHGLKPTEPDQLSRVFAATESREAAIKRSQPSEKE-----------------------------------------AKPVMEVEQAPTAARDGDNNERGIPVSEPQRPAGKLVDSAVLCSTEEVAGHDDLPSNGLAASPENLNDEDDRSSHASSSDWTPQPQIGSYSFIQQHIR-ETD-PRAILRDLLPETI----LPPDLDDMTLWQIIIN-IS------------------------EPPKRKKRKDINTL**

**1bNoth_kuh -MADEENSLGTAFSGAFITDEPATKISKMDTPTNHGLKPTDPDQLSRVFAATESREAAIKRSQPSERE-----------------------------------------AKPVMEVEQAPTAARDGDNNERGIPVSEPQRPAGKLVDSAVLCSTEEVAGHDDLPSNGLAASPENLNDEDDRSSHASSSDWTPQPQIGSYSFIQQHIR-ETD-PRAILRDLLPETI----LPPDLDDMTLWQIIIN-IS------------------------EPPKRKKRKDINTL**

**1Xenop --DSDRVGFPIAGAVLAVSKE-------NGEPLSKRQRLEDTGGGGSQLVGAESEGKAALPPIAASLQ-------------------------------EEGEASSAMESKSRAHNGSGFQGLPLGTYLVQGQEEGGAEEMPNGDLSDQAVDYGAGIHLDDDLAG-GFHSCDS---EDDDGASHASSSDWAPRPCIGPYTFVQRHLMMGTD-PRTILKDLLPDTV----APSELDDMTLWQIVINILS------------------------DPPKRKKRKDINTI**

**1Taeniop MPNTLENKISPR-------------------------------------------------CTVPLG----------------------------------------------------------------WSVIPCS------------CCLADNFLLSDEIIANGFHSCDS---DEEDRASHASSSDWTPRPRIGPYTFVQQHLMLGTD-PRTILKDLLPETI----PPPELDDMTLWQIVINILS------------------------EPPKRKKRKDINTI**

**1Gallus MADGEAPLLRPRDGGPGAAAESVEPAPKRQRLNSEDGVCGRGAPPAHRPDRGAGPPPAAAAATEPPGDAAAVSADGDVRAREEDGGATTEGRSGADNRAAQRGLARAEPPPQPRRQGRGEGAEAAPGEDAAEAAIGCERAQRSNGAAGAPAPQPDNFLLSDEIIANGFHSCDS---DEDDRASHASSSDWTPRPRIGPYTFVQQHLMLGTD-PRTILKDLLPETI----PPPELDDMTLWQIVINILS------------------------EPPKRKKRKDVNTI**

**1Equus_cab --------MVSGKSESFVGVGKHLT----AQRIYVHP----------------------------------------------------------------------------------VKRET------------------------------DDLLFGDEIITNGFHSCES---DEDDRASHASSSDWTPRPRIGPYTFVQQHLMIGTD-PRTILKDLLPETI----PPPELDDMTLWQIVINILS------------------------EPPKRKKRKDINTI**

**1Mac_mul MADEAALALQPGGSPSAAGAEREAGSPPAGEPLRKRPRRDGPGLERSPGEPGGAAPEREVPAAG-GCP-VAAAALWR------------EAEAAAAGGEQEAQATAAAGEGDNGPGLQGPSREPPLADSFYDEDDDDEGEEEEE-AAAAAIGYRDNLLFGDEIITNGFHSCES---DEEDRASHASSSDWTPRPRIGPYTFVQQHLMIGTD-PRTILKDLLPETI----PPPELDDMTLWQIVINILS------------------------EPPKRKKRKDINTI**

**1Monode MADQAALALEP-------SAGGAGPAEPGGEPLSKRQRRDGPGQGPGAERAVGAGSPGPEAAAAPLGEASEAAAPGG-----------------------DNGPGPRSQRGLPREPPPAPPDDDDDDEEEDDDEEEEEEGGEDEDEAAAAIGYRENLFS-DETLANGFHSCDS---DEDDRASHASSSDWTPRPRTGPYTFVQQHLMIGTD-PRTILKDLLPETI----PPPELDDMTLWQIVINILS------------------------EPPKRKKRKDINTI**

**1Ornitho -----------------------------------------------------------------------------------------------------------------------------------------------------------------MTRVGGCQSSG------------------------------------GTQ-SR------------------------------------------------------------------------**

**1Canis_fam MADEAALALQPGGSPSAVAAEREAASPPAGEPLRKRPRRDCPGLGRSPGEPGGAAPEREVPAATGGCS-AAAAALWR--------------EAAAGGEREAQAAVAAAGEGDNGPGLQGLTREPLPADDFVDDDDDDEGEEEEEAAAASAIGYRDNLLFDDEIITNGFHSCES---DEDDRASHASSSDWTPRPRIGPYTFVQQHLMIGTD-PRTILKDLLPETI----PPPELDDMTLWQIVINILS------------------------EPPKRKKRKDINTI**

**1Bos_ta MADEAALALQPGGSPSVVAAEREAPSPPAGEPLRKRPRRDGPGVGRSSGEPGGTAPERELPAAAGSCP-AAAAALWR--------------EAQA-------AAAAAAEEEDNGPGLQGLSREAPPADDFYDDD--DEGEEEEE--AAAAIGYRDNLLFGDEIITNGFHSCES---DEDDRASHASSSDWTPRPRIGPYTFVQQHLMIGTD-PRTILKDLLPETI----PPPELDDMTLWQIVINILS------------------------EPPKRKKRKDINTI**

**1Sus_sc MADEAALALQPGGSPSAVAAEREAPSPPAGEPLRKKPRRDGPGVGRSPGEPGGAALERELPAAAGGCP--AAAVLWR--------------ETAAGGEREAQAA-AAAGEGNNGPGLQGLSREAPPADDFYDDDDDDEGEEEEEAAAAAAIGYRDNLLFGDEIVTNGFHSCES---DEDDRASHASSSDWTPRPRIGPYTFVQQHLMIGTD-PRTILKDLLPETI----PPPELDDMTLWQIVINILS------------------------EPPKRKKRKDINTI**

**1Rattus_no --------------------------------------------------------------------------------------------------------------------------------------------------------------------------------------------------------------MIGTD-PRTILKDLLPETI----PPPELDDMTLWQIVINILS------------------------EPPKRKKRKDINTI**

**1Mus_musc MADEVALALQAAGSPSAAAAM-EAASQPADEPLRKRPRRDGPGLGRSPGEPSAAVAPAAAGCEAASAA--APAALWR---------------EAAGAAASAEREAPATAVAGDGDNGSGLRREPRAADDFDDDEGEEEDEAAAA-AAAAAIGYRDNLLLTDGLLTNGFHSCES---DDDDRTSHASSSDWTPRPRIGPYTFVQQHLMIGTD-PRTILKDLLPETI----PPPELDDMTLWQIVINILS------------------------EPPKRKKRKDINTI**

**1HUMAN MADEAALALQPGGSPSAAGADREAASSPAGEPLRKRPRRDGPGLERSPGEPGGAAPEREVPAAARGCPGAAAAALWRE----------AEAEAAAAGGEQEAQATAAAGEGDNGPGLQGPSREPPLADNLYDEDDDDEGEEEEE-AAAAAIGYRDNLLFGDEIITNGFHSCES---DEEDRASHASSSDWTPRPRIGPYTFVQQHLMIGTD-PRTILKDLLPETI----PPPELDDMTLWQIVINILS------------------------EPPKRKKRKDINTI**

**2_Aj_derSLH1 -----------------------------------------------------------------------------------------------------------------------------------------------------------------------------------------------------------------------------------------MGNENSVLVDGSTAP----------------------------VTLKSRSVEG**

**2_aMic_CCMP ----------------------------------------------------------------------------------------------------------------------------------MGGSDPLPGDDPRDEAPAGITDDARRDDTTAKTDSTVEASDGSEEEHSNRGLAEVDSTDAVASV------------SGSDDDEEVEEELTGLAALIRKLALKAEEKNATEAAAEEEP-----------------------ATFDTTPLLSTFDVAG**

**2_Ostr_tau ----------------------------------------------------------------------------------------------------------------------------------MARDDAQS-DVSVDGANARDAEDARSSDDDAVNDASSSSSSSSSSSSSGYGFGR---------------------------------ESAEMVALMERLAAQRLG-TTTEALAR--------------------------ATVDKA—LESFDLAGV**

**2_Tric_ad -------------------------------------------------------------------------------------------------------------------------------------------------------------------------------------------------------------------------------LENVRSLLQRFNIFVGEN-DSEAEQP--------------------------QQLLSEVTFEG**

**2_Nema_vect -----------------------------------------------------------------------------------------------------------------------------------------------------------------------------------------------------------------------------------MKLFNQFRLNLTQE-KSEKEKP--------------------------EQLLDEVSFEG**

**2_Branc_fl --------------------------------------------------------------------------------------------------------------------------------------------------------------------------------------------------------------------------------------VEHLRRLFARTLGLSQERET------------------EEGTARPQQVLDEVTVEG**

**2_Strong_pu ------------------------------------------------------------------------------------------------------------------------------------------MSAKNEGAAAG-----------------------------------------ADDKEGGQSESVDSQ------------------VESLRNFLGRFHLSAGSSGQEEKPKP--------------------------EQLLKELTLEG**

**2_Cio_inte -----------------------------------------------------------------------------------------------------------------------------------------------------------------MAEGSNKSDDLP-------------------------KEAE---------------------SSLSANEVDQLKNLFS-TINLG-ELIN------------------KDNEEKPEQLLPEVTFKG**

**2_Bru_mal ------------------------------------------------------------------------------------------------------------------------------------------MSEEELTGN---------SSKKNKHRSSPLMESSKP----------------------------------------------TDNKNSISPSALEMLMTKLEKLTTKEKEKD------------------------TKQKLSSLTIEG**

**2_aSchis_j -------------------------------------------------------------------------------------------------------------------------------------------------------------------------------------------------------------------------------------MSFNFDRLKKTLFGGDKCP----------------------------PKLKSFDIEG**

**2_Sch_m -------------------------------------------------------------------------------------------------------------------------------------------------------------------------------------------------------------------------------------MSFDFLGIKKALFGDNTPR----------------------------PELKSLNIEG**

**2_aCali_cle ------------------------------------------------------------------------------------------------------------------------------------------MSGSDVDKH---------EHLRNPEYFSGSSSDDED----------------------------------------------DEDLRLFGNSDEIDGYLANRILGLDMNQPL------------------------PEQLLDEVSFSG**

**2_Cali_rog ------------------------------------------------------------------------------------------------------------------------------------------MSGEEMDKE---------DRLRDPEYFSGSSSSDDDD--------------------------------------------IEEHLRLLGHSDDIDGYLASRILHLDMNQPR------------------------PAQLLDEVSFEG**

**2_Lepeo_sal ------------------------------------------------------------------------------------------------------------------------------------------MSGGDGALL---------DKLDDPEYCSGSSSEDER-----------------------------------------------NNIDLFGNNDEIDGYLANRFLDLGVNHYY------------------------PEQILDEVSFSG**

**2_Aede_ae ------------------------------------------------------------------------------------------------------------------------------------------MSAENTLPDLLGGGAQGAKPDNKPLVDGEEAGGSVTSNSVPKASPLELDTAGSGRESNNSSQVQQAPDFSQFDDSEDEEHYHSGGFHSDNISIERIRQYLSDKLGFYTTDSNY----------------DDKDGVPRKRVLETVDIDG**

**2_Apis_mel ---------------------------------------------------------------------------------------------------------------------------------------------MKLYHNGREKKSN----EETTNISSEQEEIEESD-------------------------------------------------------MEKIRKYLAQKLRLFDSS--------------------NNNEQNNPNILRELSIDG**

**2_Bomb_mo ------------------------------------------------------------------------------------------------------------------------------------------MSANSPPGKSGGHETVDESPQNVPPTTSMESLRNMFR----------------------------------------------------DLDVDDVRMYLALKLGLFSPQ-------------------DLEPAEPPEKVLDEVSLMG**

**2_Droso_me --------------------------------------------------------------------------------------------------------------------------------------------------------------------------------------------------------------------------------------MDKVRRFFANTLHLGGSS-------------------DAKEEVKVEKVIPDLSFDG**

**2_Pedi_hu_c --------------------------------------------------------------------------------------------------------------------------------------------------------------------------------------------------------------------------------------MGANCSLLPVACSFCGQS----------------------KKEKAVQVLDEVSITG**

**2_Trib_ca ------------------------------------------------------------------------------------------------------------------------------------------MSRDSSK------DRAEAKPKQPPEAQSEEDNASGTS-----------------------------------------------------MSIDSLRKYLAEKLGISDKD-------------------DEKE---KIKILDDVSVDG**

**2_Danio_rer -------------------------------------------------------------------------------------------------------------------------------------------------------------MS--EEVSKRVEEEAD---TP-----------GLEGQSDDSSDEG---------------------DASGDTEMDFLRSLFSRTLGLSPGD----------------------------KVLDELTLDS**

**2_Salmo_sal -------------------------------------------------------------------------------------------------------------------------------------------------------------MSDVPEASNREEVEDN---TP-----------EPEEQSDDSSDDG---------------------EASGDTEMDFLRNLFSKTLGIS-GE----------------------------KVLDELTLEG**

**2_Xe_lae -----------------------------------------------------------------------------------------------------------------------------------MERNPVR---------------KAIPERQLQETCDRSETSDEGKRSPSSHKTESSEPADLKAQAEDSEDSDSSE------------------DNSGASEMDFLRNLFSRTLGIGTPE----------------------------KVLDELSLES**

**2_Gallus ------------------------------------------------------------------------------------------------------------------------------------------MSEADG------------NGGGGVEPGGSLRRSVTPRPIPAAPP--------PGADPDAEAEGG---------------------SEE-DSDMELLRNLLARTLGLGTEPP--------------------------ERVLDELSLAG**

**2_Bostau ------------------------------------------------------------------------------------------------------------------------------------------MADPDP------------SDP--EETQAGKVQEAQ------------------DSDSD--TEAG---------------------ATGGEAEMDFLRNFFSQTLGLGTQK---------------------------ERLLDELTLEG**

**2_Canis_fam --------------------------------------------------------------------------------------------------------------------------------------------------------------------------------------------------------------------------------MAACAEPSGTRSLAVRVF-LARER---------------------------QAPLDEK----**

**2_Equusca ------------------------------------------------------------------------------------------------------------------------------------------MAEPDP------------SDP--LETQAGKVQEAQ------------------DSDSD--TEGG---------------------AAGGEAEMDFLRNFFSQTLGLGTQK---------------------------ERLLDDLTLEG**

**2_Rattusnorv -----------------------------------------------------------------------------------------------------------------------------------------MKAVTGPRCLSGVVFFYPLSAPSCFRCRHGTEQSVTVPRAPTPFPW----PSRTDSDSD--TEGG---------------------ATGGEAEMDFLRNLFTQTLGLGSQK---------------------------ERLLDELTLEG**

**2_Musmuscu ------------------------------------------------------------------------------------------------------------------------------------------MAEPDP------------SDP--LETQAGKVQEAQ------------------DSDSD--TEGG---------------------ATGGEAEMDFLRNLFTQTLGLGSQK---------------------------ERLLDELTLEG**

**2_Susscr ------------------------------------------------------------------------------------------------------------------------------------------MAEPDP------------SDP--VETQTGKVQEAQ------------------DSDSDSDTEEG---------------------AAGGEAEMDFLRNFFSQTLGLGTQK---------------------------ERLLDELTLEG**

**2_HUMAN ------------------------------------------------------------------------------------------------------------------------------------------MAEPDP------------SHP--LETQAGKVQEAQ------------------DSDSD--SEGG---------------------AAGGEADMDFLRNLFSQTLSLGSQK---------------------------ERLLDELTLEG**

**3_Ciona_inte ----------------------------------------------------------------------------------------------------------------------------------MKCLGRCWLG-GKVGKLFIIQIKQECQKSLFLLKMNKSYDEGTGQRNGATTKKMENDQSFVEGDFSKSCSNKLKSGVEKTKKKTVKKKKKKKQEQKDEVTKISKSFSKLDINRRDVSKQRT----------------PQRRSQLGKTPSSLSSIKD**

**3_Xeno_tro ----------------------------------------------------------------------------------------------------------------------------------MSFHTSHFSGQDPVGKICSTPLSPKAATCVDQWPDAKMEVSATNS---AFQTTVINNQ--------------------NAQEAADLKHPTEYHSRKRENGRLSGVIRQSCLRKRQLPKSAL----------------AKNISAIKPN-IGCNNLED**

**3_Danio_re --------------------------------------------------------------------------------------------------------------------------------------------------MSKARLS----------RDRRAASVGVSR---VTRSSMMSPQ--------------------DCERSRAPDP-----GLLDELSLMSVSEQQASATRKGSSKPAL----------------S---SPSGRS-VSRGALET**

**3_Gallus ----------------------------------------------------------------------------------------------------------------------------------MERGVRRGAALVAAWRSLWER--------------------------------------------------------GGLALFRPQCRTGCGACRVQGTRPFSLSAAASAVLGLGS--------------------------WGGDSGKQKLTLQD**

**3_Taeniopy ---------------------------------------------------------------------------------------------------------------------------------------------MAVPSPAGHR--------------------------------------------------------AVIPLDKDTQSP-----RIQGSRPFCLSTAARAILGW----------------------------WGGDEGKQKLTLKD**

**3_Equ_cab ------------------------------------------------------------------------------------------------------------------------------------------------------------------------------------------------------------------------MVVLDS-GITGGRRPISFSAGASSVFGS-----------------------------GGNSRKEKLLLQD**

**3_isCan_fa ----------------------------------------------------------------------------------------------------------------------------------MTRCARPALAALGLWGPAGWRSLYTGVRDVLGEGHQRPSPGRMDMGAASLWG-------------------------VRKAPCAVELAVLNS-GITGGRRPISFSTRTSSIFGS-----------------------------GGD-HKKKLFLQD**

**3_Mus_mus -------------------------------------------------------------------------------------------------------------------------------------------------------------------------MALDPLGAVVLQS-------------------------IMALSGRLALAALRLWGPGGGRRPISLCVGASGGFGG-----------------------------GGS-SEKKFSLQD**

**3_aRat_norv ----------------------------------------------------------------------------------------------------------------------------------------------------------------------------------------------------------------MALSCRLVLAALRLWGPGGGRRPISLSVGASGGFGC-----------------------------GGH-SEKKLSLQD**

**3_Ory_cun ----------------------------------------------------------------------------------------------------------------------------------MALWSQRVVAALRLWG-----------------------------------------------------------------------P-------IGGRRPVSLSARAPSISGS-----------------------------RGRPER—FSLQDV**

**3_Monod_dom -----------------------MGQTSRRKRTLTSGAARTYFRRARTRYFRLRPRVRQAGVPSGHSGSWSTPVLRPGPPWVLRWGPREGSPRPPAVSVPPCACVGVRAAAGAEEGPLSRFPTAPLPSQSTIPRASQPVGKWNQWGVLRDRRLTLLGRACGSPLPPPVASLGALHRPLPSLCQGGGVSACAVREAPLEDLPSLEFGGAFEPSTDPAASLALCGGRCLHRGLWEGKLGPGLAFGC-----------------------------EVSTSSKKLSLQD**

**3_Mac_mu ----------------------------------------------------------------------------------------------------------------------------------MAFWGWRAVAAVRVWGRVAERAEAGGDVGPFQAWSCRLVLGGRDDVSAGPRGSRGVCGEPLDSARPLQRPPRPAVPRAFRRQPRAAAPGFFFSSIKGGRRPISFSVGASSVVGS-----------------------------GGDSDKGKLSLQD**

**3_Sus_scr ----------------------------------------------------------------------------------------------------------------------------------------------------------------------------------------------------------------------------------------------------------------------------------------------**

**3_HUMAN ----------------------------------------------------------------------------------------------------------------------------------MAFWGWRAAAALRLWGRVVERVEAGGGVGPFQACGCRLVLGGRDDVSAGLRGSHGARGEPLDPARPLQRPPRPEVPRAFRRQPRAAAPSFFFSSIKGGRRSISFSVGASSVVGS-----------------------------GGSSDKGKLSLQD**

**SIRT4ARAB ------------------------------------------------------------------------------------------------------------------------------------MKLHQDKNRRESHERIFDRELTRSSSRERDRSEGEIEAKERELRPQRENKIDTSDFCHLVFNNNKKFWKKIKRKTEEISFHPPLT----------TSQDLF-PSR-SMYRPLQSGGNLVMLFKGCRRFVRTTCRVSIPGGSLGNESKAPPRFLR**

**SIRT4ORIZA --------------------------------------------------------------------------------------------------------------------------------------------------MAAGAHASRASAPIIAGLTGALRAAYKGFSPQLCN-------FHASVNNG-----LLHRRKIQLHFICSFR----------SIQARY-NHS-SAVAPKDY----------CETYIQ---------------------FLR**

**SIRT4PHYSCOM ------------------------------------------------------------------------------------------------------------------------------------------------------MWKPNHSIRCLQLQTG----ARNKLSPCLLN-------------------------STRFCNYATLA----------ATGSVA-VPQ-EISSSLNS----------------------------------------**

**4_Stig_aura -----------------------------------------------------------------------------------------------------------------------------------------------------------------------------------------------------------------------------------------------------------------------------MSALPVPPSPTSVPSNV**

**4_Stron_pu ----------------------------------------------------------------------------------------------------------------------------------------------------------------------------------------------------------------------------------------------MGEREGG------------------------FLSQFVPESRPVTESSI**

**4_Acy_pisum --------------------------------------------------------------------------------------------------------------------------------------------------------------------------------------------------------------------------------------------------------------------------------------MPVQSSDA**

**4_Apis_ --------------------------------------------------------------------------------------------------------------------------------------------------------------------------------------------------------------------MRSYKS-----------------ANIIETLLYF---------------------------LFVPKCEPTKDSYL**

**4_Droso_me ---------------------------------------------------------------------------------------------------------------------------------------------------------------------------------------------------------------MRVGQLLRFRS-----------------TSLRSSTARQ---------------------------EYVPHHKPVVEDDI**

**4_Tribol -----------------------------------------------------------------------------------------------------------------------------------------------------------------------------------------------------------------MSNLTRQLP-----------------FLLKRTPILS---------------------------DFIPRHSPAPSTDV**

**4_Danio_re -----------------------------------------------------------------------------------------------------------------------------------------------------------------------------------------------------------------MLLSCRYLP------------PPVAVGRCASTIQAG-------------------------VRQFVPASGSFDSSAL**

**4_Xeno_tro -----------------------------------------------------------------------------------------------------------------------------------------------------------------------------------------------------------------MWKNVREG-----------SKVFWGINNITRSHKSH-----------------------LALSEFVPACPPPNPHQV**

**4_Gallus ---------------------------------------MAGYWEKFSTSNNRASDKPHWLRYCHCAKLTAPSAPPTRPEAAPPPTSQRAGALPRAPHLRPRAPPAAAAPRSERRCVRGGARRSGAAAELERGVCREPAMVMSAGRAAPAREEGLRRAAEGGCEALGAVPGLYPCRKLGFCSLNHGNCSRCAELCTDCISHKALLAGSRSRSWQRYERAFRVCAKANAVCGPR-SLNMFPARRLPGGCRAIRPHHLRH----HSVPSASPNLAFVPACLPPHPAEV**

**4_Tae_gut ---------------------------------------MQHTWEKQ--REDEESSKRGWA--------------PLIPAALPTP-------YPRKVQISPKN-------RLER---------------LRRG--REEMFAETRVRST------LLRAQE---------------RRAPFP-------AGTATACPGCGWTAVPPACPASSAPRRYR--------GAPFCPPF-WAPVTPAKAAG-----------------REYAKASPNLTFVPACLPPDPAEV**

**4_MOUSE -----------------------------------------------------------------------------------------------------------------------------------------------------------------------------------------------------------------MS-GLTFR----------PTKGRW-ITHLSRPRSCG------------------------PSGLFVPPSPPLDPEKI**

**4_Bos_tau ---------------------------------------------------------------------------------------------------------------------------------------------------------------------------------------------------------------MRMSFGLTFKR---------TAKVHW-RANFSQQCSLR------------------------STGLFVPPSPPLDPEKV**

**4_Equus_cab ---------------------------------------------------------------------------------------------------------------------------------------------------------------------------------------------------------------MRMSFGLTFR----------TAKGHW-LVNLSRQCSHG------------------------STGLFVPPSPPLDPEKV**

**4_Pan_trog ---------------------------------------------------------------------------------------------------------------------------------------------------------------------------------------------------------------MKMSFALTFR----------SAKGRW-IANPSQPCSKA------------------------SIGLFVPASPPLDPEKV**

**4_Cani_fam -----------------------------------------------------------------------------------------------------------------------------------------------------------------------------------------------------------------MNLGLTLK----------APKGLL-MVNISRQYSRR------------------------SIGFFVPSSPPLDPEKV**

**4_Ovis_ari ---------------------------------------------------------------------------------------------------------------------------------------------------------------------------------------------------------------MRMSFGLTFKR---------TAKVRW-RANFSQQCSLR------------------------STGLFVPPSPPLDTEKV**

**4_Macaca_mu ---------------------------------------------------------------------------------------------------------------------------------------------------------------------------------------------------------------MKMSFGLTFK----------SAKGRW-IANPSWQCSKA------------------------SIGLFVPASPPLDPEKV**

**4_Mus_mus ----------------------------------------------------------------------------------------------------------------------------MAWVEAQPQREKEGVSSISGTLTELSRKDSGQSVEERPRILAWETRRILGLVVFFQLDRLRGLHFPRCTRNKRVPLLGENCGRIRMS-GLTFR----------PTKGRW-ITHLSRPRSCG------------------------PSGLFVPPSPPLDPEKI**

**4_Ratt_nor -----------------------------------------------------------------------------------------------------------------------------------------------------------------------------------------------------------------MR-GLIFR----------PTRGRW-ITQMSQLRSHG------------------------STGLFVPPSPPLDHEKI**

**4_Sus_scr ---------------------------------------------------------------------------------------------------------------------------------------------------------------------------------------------------------------MRMSFGLIFR----------TAKGRW-MAKLSRQCSRG------------------------YTELFVPSSPPLDPEKA**

**4_HUMAN ---------------------------------------------------------------------------------------------------------------------------------------------------------------------------------------------------------------MKMSFALTFR----------SAKGRW-IANPSQPCSKA------------------------SIGLFVPASPPLDPEKV**

SIRT5PHYSCOM -----------------------------------------------------------------------------------------------------------------------------------------------------------------------------------------------------------------------------------MPRAGVAASAFKLRNIWH-------------------IHNLHRVRSQQTRLMS---DSV

**SIRT5ORIZA ----------------------------------------------------------------------------------------MYYGFDIGGTKIALGVFDKDLRLQWETRVPTPRESYDEFLTAIAALVAQADERFGVKGSVGIGIPGMPETDDGTLYAANVPAASGKALRADLSARLERDVRLDNDANCFALSEAWDDEFRQYPLVMGLILGTGVGGGIVINGKPITGRSYITGEFGHIRLPVDALDIVGREFPLTRCGCGQHGCIENYLSGRGFAWLY**

**5_Perkin_m --------------------------------------------------------------------------------------------------------------------------------------------------------------------------------------------------------------------------------------------------------------------------------------MMMSIRGL**

**5_Ciona_int --------------------------------------------------------------------------------------------------------------------------------------------------------------------------------------------------------------------------------------------------------------------------------------MSRPSSDM**

**5_Apis_mel ----------------------------------------------------------------------------------------------------------------------------------------------------------------------------------------------------------------------------------------------------------------------------MN--------------TM**

**5_Nason_vit ----------------------------------------------------------------------------------------------------------------------------------------------------------------------------------------------------------------------------------------------------------------------------MSSLPRTDTCITERYATY**

**5_Anopl ----------------------------------------------------------------------------------------------------------------------------------------------------------------------------------------------------------------------------------MIVRRLTCGGLVHSHLHAN-------------------LKRLQGSQ-----VMARPSSDL**

**5_Danio_re ----------------------------------------------------------------------------------------------------------------------------------------------------------------------------------------------------------------------------------MIVRQLWCSRGSTSHLCAA-------------------VRLNWRSP-----KMTRPSSDL**

**5_Salm ----------------------------------------------------------------------------------------------------------------------------------------------------------------------------------------------------------------------------------MIVRQFSSRG-VTSHLCAR-------------------FKETQTRQ-----VMARPSSDL**

**5_Taeniop -------------------------------------------------------------------------------------------------------------------------------------------------------------------MSSRCVRRSGHHGSPHPGVKWGGRSPELRARRSRLPSRRPRPAALPLRPSAGRQRPVKHFRGIPMCLFQSAARRLVPQVRCG-------------------LKASSSKKQKFGLEMARPSSNM**

**5_Bos_ta -----------------------------------------------------------------------------------------------------------------------------------------------------------------------------------------------------------------------------------MPPLWIIRNRLFSQLYCG-------------------LKSPVSTQTKICLTMARPSSNM**

**5_Canis_f -----------------------------------------------------------------------------------------------------------------------------------------------------------------------------------------------------------------------------------MQPLQIAPCRLLYGLYRG-------------------LKSPASTGTRICPAMARPSSNM**

**5_Pan_trog -----------------------------------------------------------------------------------------------------------------------------------------------------------------------------------------------------------------------------------MRPLQIVPGRLISQLYCG-------------------LKPPASTRNQICPKMARPSSSM**

**5_Macac_mu -----------------------------------------------------------------------------------------------------------------------------------------------------------------------------------------------------------------------------------MRPLQIVPSRLISQLYCG-------------------LKPPASTRNQICLKMARPSSSM**

**5_Mus_mus -----------------------------------------------------------------------------------------------------------------------------------------------------------------------------------------------------------------------------------MRPLLIAPGRFISQLCCR-------------------RKPPASPQSKICLTMARPSSNM**

**5_Ornit_an ---------------------------------------------------------------------------------------------------------------------------------------------MSMEVIKRPDLSHAGAGEGYGLSEMGRSAAVARGRWARANSKLQPPMVLRHRHQSLGSPNPCRVLPTSALPFSSGTLPGQCMAPAEQIPMPVTPANLVSRLGGE-------------------LKLPASKRLKICIGMARPSSNM**

**5_Rattus_nor -----------------------------------------------------------------------------------------------------------------------------------------------------------------------------------------------------------------------------------MRPLPVAPGRLFSQLCCG-------------------PKPSASPQSKICLTMARPSSNM**

**5_Sus_sc -----------------------------------------------------------------------------------------------------------------------------------------------------------------------------------------------------------------------------------MPPLWGVPGRLLSQLRCG-------------------LTSPASAPTRICPTMARPSSSM**

**5_HUMAN -----------------------------------------------------------------------------------------------------------------------------------------------------------------------------------------------------------------------------------MRPLQIVPSRLISQLYCG-------------------LKPPASTRNQICLKMARPSSSM**

**SIRT6VITIS ----------------------------------------------------------------------------------------------------------------------------------------------------------------------------------------------------------------------------------MSLGYAEKLSYIEDVGKVGMS--------------------------EICDPLHVLQEKV**

**SIRT6RICINUS -----------------------------------------------------------------------------------------------------------------------------------------------------------------------------------------------------------------------------------MSLGYAEKLSFIEDVGNVGM--------------------------AEFFDSSYVLQEK**

**SIRT6ARAB -----------------------------------------------------------------------------------------------------------------------------------------------------------------------------------------------------------------------------------MSLGYAEKLSFIEDVGQVGM--------------------------AEFFDPSHLLQCK**

**SIRT6ZEAMAYS -----------------------------------------------------------------------------------------------------------------------------------------------------------------------------------------------------------------------------------MSLGYAEKLSYREDVGTVGM--------------------------PEIFETPELVQNK**

**SIRT6TRITICUM -----------------------------------------------------------------------------------------------------------------------------------------------------------------------------------------------------------------------------------MSLGYAEKLSYREDVGTVGM--------------------------PEKFDSPKLLQGK**

**SIRT6ORIZA -----------------------------------------------------------------------------------------------------------------------------------------------------------------------------------------------------------------------------------MSLGYAEKLSYREDVGNVGM--------------------------PEIFDSPELLHKK**

**SIRT6PHYSCOM ----------------------------------------------------------------------------------------------------------------------------------------------------------------------------------------------------------------------------------MSSLGYAEKLSYRADVGTVGM--------------------------PELYDPAEDLQSK**

**6_Stron_p ----------------------------------------------------------------------------------------------------------------------------------------------------------------------------------------------------------------------------------------------------------------------------------------------**

**6_Lepeo_sal ----------------------------------------------------------------------------------------------------------------------------------------------------------------------------------------------------------------------------------MSCSYAEGLSDYANKGKLGLP--------------------------ESFDSPEDLKSKV**

**6_Acyrt_p ----------------------------------------------------------------------------------------------------------------------------------------------------------------------------------------------------------------------------------MSCNYADGLSPYEYKGEVGMN--------------------------EVFDTPEVLKQKI**

**6_Apis_mel ----------------------------------------------------------------------------------------------------------------------------------------------------------------------------------------------------------------------------------MSCSYADGLSQYENKGVLGLE--------------------------ERYDSVEALRLKC**

**6_Dros_mel ----------------------------------------------------------------------------------------------------------------------------------------------------------------------------------------------------------------------------------MSCNYADGLSAYDNKGILGAP--------------------------ESFDSDEVVAEKC**

**6_Danio_re ----------------------------------------------------------------------------------------------------------------------------------------------------------------------------------------------------------------------------------MSVNYAAGLSPYADKGICGLP--------------------------ETFDSPEELKTKV**

**6_Xenop_tr ----------------------------------------------------------------------------------------------------------------------------------------------------------------------------------------------------------------------------------MSVNYAAGLSPYSDKGRCGLP--------------------------EAFDPPDELCRKV**

**6_Gallus ----------------------------------------------------------------------------------------------------------------------------------------------------------------------------------------------------------------------------------MAVNYAAGLSPYSDKGKCGLP--------------------------EIFDPPEELERKV**

**6_Bos_ta ----------------------------------------------------------------------------------------------------------------------------------------------------------------------------------------------------------------------------------MSVNYAAGLSPYADKGKCGLP--------------------------EVFDPPEELEQKV**

**6_Canis_fam ----------------------------------------------------------------------------------------------------------------------------------------------------------------------------------------------------------------------------------MSVNYAAGLSPYADKGKCGLP--------------------------EIFDPPEELERKV**

**6_Mus_mus ----------------------------------------------------------------------------------------------------------------------------------------------------------------------------------------------------------------------------------MSVNYAAGLSPYADKGKCGLP--------------------------EIFDPPEELERKV**

**6_Pan_trogl ----------------------------------------------------------------------------------------------------------------------------------------------------------------------------------------------------------------------------------MSVNYAAGLSPYADKGKCGLP--------------------------EIFDPPEELERKV**

**6_Sus_sc ----------------------------------------------------------------------------------------------------------------------------------------------------------------------------------------------------------------------------------MSVNYAAGLSPYADKGKCGLP--------------------------EVFDPPEELEQKV**

**6_Macaca_mu ----------------------------------------------------------------------------------------------------------------------------------------------------------------------------------------------------------------------------------MSVNYAAGLSPYADKGKCGLP--------------------------EIFDPPEELERKV**

**6_Rattus_no ----------------------------------------------------------------------------------------------------------------------------------------------------------------------------------------------------------------------------------MSVNYAAGLSPYADKGKCGLP--------------------------EIFDPPEELECKV**

**6_HUMAN ----------------------------------------------------------------------------------------------------------------------------------------------------------------------------------------------------------------------------------MSVNYAAGLSPYADKGKCGLP--------------------------EIFDPPEELERKV**

**SIRT7PHYSCOM ------------------------------------------------------------------------------------------------------------------------------------------------------------MWKQWIQTTRSQMRTHG-LQKSTRVLFDGHKVLLFKDELRRHEGIHEKLIDSAC-E--AAGGAQECGCQGAEIPCGRLAKCWVALTERGRE------------------------AQEMEDEEEGPVLRA**

**7_Ciona_inte -----------------------------------------------------------------------------------------------------------------------------------------------------MQSVFLPATECGSSDDVFIGADNEEKEVEDEVATPRTLRPRKLAPTVTKRDLTKQIREVVARRHHSLDEKLFLDEHCSLVKAVRARASSYSKLKQRSQ--------------------------EVFDDPVTLHAKC**

**7_Apis_mel ---------------------------------------------------------------------------------------------------------------------------------------------------------------------MEETSNEKFLSRR-RSAALKAFKVKDE-RVATFKKVAAILQKSETDR--TAEETGILISCSDVVKEVNLRQEKRHRVKARLE--------------------------EIEDAPELLEEKC**

**7_Danio_re ---------------------------------------------------------------------------------------------------------------------------------------------------------------------MDVRINSGVSARAERKEQEKAKIIQREKQRQTMKTISKILQKCESEW--TEEERSMLQAHQDTVQELSRRQNRRHLLKRKQE--------------------------EVFDDAENLKTKV**

**7_Xenopus_t ------------------------------------------------------------------------------------------------------------------------------------------------------------------------------MGRAERKAEAREEILRREQNRDRLRRVCVILHKPAADR--SPEESELLGKCGDLVHELEKRRLHVERRRHREQ--------------------------EVLDDTDLLREKV**

**7_Bos_ta ------------------------------------------------------------------------------------------------------------------------------------------------------------------------MAAGG-LSRSERKAAERVRRLREEQQRERLRQVSRILRKAATER--SAEEGRLLAESEDLVTELQGRSRRREGLKRRQE--------------------------EVCDDPEELQRKV**

**7_Canis_fa ------------------------------------------------------------------------------------------------------------------------------------------------------------------------MAAGG-PSRSERKAAERVRRLREEQQRERLRQVSRILRKAVAER--SAEEGRLLAESEDLVTELQGRSRRREGLKRRQE--------------------------EVCDDPEELRRKV**

**7_Equus_cab -----------------------------------------------------------------------------------------------------------------------------------------------------------------------------------------MPYCRSVERRE----------KAGKAG--PAP----WSRSENLVTELQAEQRR--GLKRRQE--------------------------EVCDDPEELRRKV**

**7_Macaca ------------------------------------------------------------------------------------------------------------------------------------------------------------------------MAAGG-LSRSERKAAERVRRLREEQQRERLRQVSRILRKAAAER--SAEEGRLLAESADLVTELQGRSRRREGLKRRQE--------------------------EVCDDPEELRGKV**

**7_Mus_mus ------------------------------------------------------------------------------------------------------------------------------------------------------------------SAVKGAMAAGGGLSRSERKAAERVRRLREEQQRERLRQVSRILRKAAAER—SAEEGRLLAESEDLVTELQGRSRRREGLKRRQEEASRGQRVLGGGLREASGGNNQPGSYQVCDDPEELRRKVR**

**7_Pan_t ------------------------------------------------------------------------------------------------------------------------------------------------------------------------MAAGG-LSRSERKAAERVRRLREEQQRERLRQVSRILRKAAAER--SAEEGRLLAESADLVTELQGRSRRREGLKRRQE--------------------------EVCDDPEELRGKV**

**7_aRattus ------------------------------------------------------------------------------------------------------------------------------------------------------------------------MAAGGGLSRSERKAAERVRRLREEQQRERLRQVSRILRKAAAER--SAEEGRLLAESEDLVTELQGRSRRREGLKRRQE--------------------------EVCDDPEELRRKV**

**7_Sus_sc ------------------------------------------------------------------------------------------------------------------------------------------------------------------------MAAGG-LSRSERKAAERVRRLREEQQRERLRQVSRILRKAAAER--SAEEGRLLAESEDLVTELQGRSRRREGLKRRQE--------------------------EVCDDPEELRRKV**

**7_HUMAN ------------------------------------------------------------------------------------------------------------------------------------------------------------------------MAAGG-LSRSERKAAERVRRLREEQQRERLRQVSRILRKAAAER--SAEEGRLLAESADLVTELQGRSRRREGLKRRQE--------------------------EVCDDPEELRGKV**

**B) SIRTUINS – CARBOXY TERMINAL SEGMENTS: FIRST PART starting from the core on the left side of the table to the end)**

**A) UNORDERED STRUCTURE IN YELLOW,**

**B) NES (NUCLEAR EXPORT SIGNAL) IN RED**

**and C) SIRT1 REPEATS of *Strongylocentrotus* shown in various alternating colors**

**1-Hydra_magnipapillata DVIGT-------------YKP-----------------------------------------------------------------------------------------------------------------------------------------------------------------SFLSVSKTIKTFSNTSD--------SNKSLSGFDK-----------------------------------------------------------------------ECYNSSN------------------------------------------HVLQDQ-**

**1-Strongylocentrotus HVCQSQQRLTETTHIPNGFKPKESRQARKAVLEEGGTTVDQRPDPVLDEGETTVDQRPDPVLDEGETTVDQRSNPVLDEGETTVDQKPDPVLDEGETTVDQKPDPVLDEGETTVDQKPDPVLDEGETTVDQKPDPAGRIDQEESVSNP---------------------------------VNVKSKEDTQHSLSSLKE--------EGQVSSGDDKNEGSKAGEGDDRLARSNSQDSPISPEGHVKQSCDERLQAPENQTSLEHHINGLTNEGKIHVDALPCSRSETNCSPSSNSDRLQSVDEVENALASSEPQTSKTIPSESSQS----------HTAQDQT**

**1-Brugia_malayi DIFRKGRFKTNSND-----------------------------------------------------------------------------------------------------------------------------------------------------------------------------PTAIEEIYEKTT---------------------------------------------------------------------------------------LRKAINEIQFKKLLDQPTVKRARLKEG-------------------------------**

**1-aSchistosoma_mansoni GLSDYTP--------------------------------------------------------------------------------------------------------------------------------------------------------------------------------LTEIPLNSLKNTTEPL-----------KADEKSPNVS-------------------------------------------------------------------HLIVQDTVSSELVNDESQRNDNSEVN-------------------------------**

**1-Schistosoma_japonicum --------------------------------------------------------------------------------------------------------------------------------------------------------------------------------------------------------------------------------------------------------------------------------------------------------------------------------------------------------**

**1-Acyrthosiphon_pisum ELCWCKEELTEAKT----LNTPT--------------------------------------------------------------------------------------------------------------------------------------------------------------SSPRSNEATIETVEG--------------------------------------------------------------------------------------------EMSTDSTRDSANSMSPP---------LGDDRFTPG---------------TSGEQR**

**1-Tribolium_castaneum EGVFDDNMLEEASH----LLPLQEPSFD---------------------------------------------------------------------------------------------------------------------------------------------------------MTPPCVKSQTEPNCTDD------------NLEKASPCLCS------------------------------------------------------------------TKCSSSSTVEKDEELTLECSCNKQTSIINKQSLLDSDLVN------SLDLKSVKER-**

**1-Apis_mellifera EVCWNDTILKETTQ----LLALRYITDD---------------------------------------------------------------------------------------------------------------------------------------------------------TWEQSQDTSSNTILSRDSVEINFKAHDPQSIESQDNLMIHNNI---------------------------------------------------VEHYEENMNARISPFCNDHTNSIEEKFNLLGESPKRR--LGESSIESSPKRMNFGSNCVLHFNTCPSKT**

**1-Pediculus ELCWRDGKLVEATE----LLPVS--TDE---------------------------------------------------------------------------------------------------------------------------------------------------------SNNFKLDSEAENLTS--------------QINSQTEIFVG----------------------------------------------------------------KVSNQRCGHT-VQESKTDCNENCRKRS--LGTSDCPGNDSKR---------QKLTEEKT**

**1-Danio_rerio QLCYNSSRLSEITE-----KPAAPEHTEN--------------------------------------------------------------------------------------------------------------------------------------------------------TSADHSHADAEHIEN-------------TSADHSHADAE-------------------------------------------------------------------HIENTS-ADR--DDAKHTENTPTDHA-----------DAE----------HTKNTSA**

**1-aNothobranchius_f QLCFNTLRLNEITE-----KPPRLQEPRP--------------------------------------------------------------------------------------------------------------------------------------------------------SEALPAFSDAAQQQNEPS--------RTDSVNKPSEEAE-------------------------------------------------------------------SLTVTETADKNITPLEPCSNVQCPSE-----------DGA----------KSPELPK**

**1-bNothobranchius_k QLCFNTLRLNEITE-----KPPRLQEPRP--------------------------------------------------------------------------------------------------------------------------------------------------------SEALPAFSDAAQQQNEPS--------RTDSVNKPSEEAE-------------------------------------------------------------------SLTVTETADKNITPLEPCSNVQCPSE-----------DGA----------KSPELPK**

**1-Xenopus_tropicalis QLCTNSLKLSQITE-----KPPRIHKGFL--------------------------------------------------------------------------------------------------------------------------------------------------------TSPETVPSTDLNTGQ--S--------PALQSDLRGTDLQ-------------------------------------------------------------------LSASNTMRSLEKPEEASKLSHNCSEE-----NLEVSKEAN----------TQLSNEK**

**1-aeniopygia_guttata KLCYNSVKLSEITE-----KPPRPHKELE--------------------------------------------------------------------------------------------------------------------------------------------------------ALSAELPPTPLNISEGSS--------SPERMSP-ANSAR-------------------------------------------------------------------ASEHPPECKVENCQPAPETTGTCSEE--TLQDTQVSSENP----------ENPASEL**

**1-Gallus_gallus KLCYNSVKLSEITE-----KPPRMHKELE--------------------------------------------------------------------------------------------------------------------------------------------------------MHSSELPPTPLDISEDSG--------SPEQMTPPGTSVV-------------------------------------------------------------------PSEHAAECKVENSDPASETKGICTEE--KLQDTQASSENP----------ENPASEL**

**1-Equus_caballus KLCCNPIKLSEITE-----KPPRTQREL---------------------------------------------------------------------------------------------------------------------------------------------------------AHLSELPPTPLNISEDSS--------SPERTSPPDSSVI-------------------------------------------------------------------VTLLDQATKSNVDDPDVSKSKDCMEE--KSQEVQTSTRSF----------QSVTERL**

**1-Macaca_mulatta KLCCNPVKLSEITE-----KPPRTQKEL---------------------------------------------------------------------------------------------------------------------------------------------------------AYLSDLPPTPLHISEDSS--------SPERTSPPDSSVI-------------------------------------------------------------------VTLLDQAAKSN-DDLDVSESKGRMEE--KPQEVQTS-RNV----------ESIAEHM**

**1-Monodelphis_domestica RLCSNPTRLSEITE-----KPPRPQRELG--------------------------------------------------------------------------------------------------------------------------------------------------------ARWGALPPTPLHVSEDSS--------SPGRTSPPDSWAG-------------------------------------------------------------------ERRAGEADDG-------AASRGSCRADKPPPE-----VHP----------ASITEQL**

**1-Ornithorhynchus QLCSNPVKLSEITE-----KPPRTHKELE--------------------------------------------------------------------------------------------------------------------------------------------------------ARWAALPPTPLHVSEGSS--------SPDRTSPPDALAA-------------------------------------------------------------------PARPEQMTDSRVVQVESGAEAGGCPGGAAPQAGQSSSESA----------AAVSQQL**

**1-Canis_familiaris KLCCNPVKLSEITE-----KPPRTQKEL---------------------------------------------------------------------------------------------------------------------------------------------------------AHLSELPPTPLNISEDSS--------SPERTSPPDSSVI-------------------------------------------------------------------VTLLDEATKSNVDDPGVSESRDCMEE--KSQEGQNSIRNI----------ESVTEHL**

**1-Bos_taurus KLCCNPVKLSEITE-----KPPRIQKEL---------------------------------------------------------------------------------------------------------------------------------------------------------AHLSELPPTPLNISEGSS--------SPERTSPPDSSVI-------------------------------------------------------------------VTLLDQETKSNVDDPDVSESKDHVTE--KSQEVQTSTRSI----------ESVNEQL**

**1-Sus_scrofa KLCCNPVKLSEITE-----KPPRTQKEL---------------------------------------------------------------------------------------------------------------------------------------------------------AHLSELPPTPLNISEGSS--------SPERTSPPASSVT-------------------------------------------------------------------LPLLDQATKSNVDDLDVSESKDCVEE--KSQEVQTS-RSI----------ESVKEPM**

**1-Rattus_norvegicus KLCCNPVKLSEITE-----KPPRTQKEL---------------------------------------------------------------------------------------------------------------------------------------------------------VHLSELPPTPLHISEDSS--------SPERTVPQDSSVI-------------------------------------------------------------------ATLVDQTIKNKVDDLEVSEPKSCVEE--KSQEVQTY-RNV----------ESIN--V**

**1-Mus_musculus KLCCNPVKLSEITE-----KPPRPQKEL---------------------------------------------------------------------------------------------------------------------------------------------------------VHLSELPPTPLHISEDSS--------SPERTVPQDSSVI-------------------------------------------------------------------ATLVDQATNNNVNDLEVSES-SCVEE--KPQEVQTS-RNV----------ENIN--V**

**SIRT1_HUMAN KLCCNPVKLSEITE-----KPPRTQKEL---------------------------------------------------------------------------------------------------------------------------------------------------------AYLSELPPTPLHVSEDSS--------SPERTSPPDSSVI-------------------------------------------------------------------VTLLDQAAKSN-DDLDVSESKGCMEE--KPQEVQTS-RNV----------ESIAEQM**

**2_aAjellomyces_ER ----DDVLLLGECD------------------------------------------------------------------------------------------------------------------------------------------------------------------------------DGVLKLAEALG-------------------------------------------------------------------------------------------------------------------------------------------------**

**2_aMicromonas_CCMP1545 D-NRRDALFLGDCD------------------------------------------------------------------------------------------------------------------------------------------------------------------------------AGFAELARLLG-------------------------------------------------------------------------------------------------------------------------------------------------**

**2_Ostreococcus_tauri DTNYRDALYLGACD------------------------------------------------------------------------------------------------------------------------------------------------------------------------------DGIAELSELLG-------------------------------------------------------------------------------------------------------------------------------------------------**

**2_Trichoplax_adhaerens ESNYRDVFWQGTCD------------------------------------------------------------------------------------------------------------------------------------------------------------------------------DGCFTLAEALG-------------------------------------------------------------------------------------------------------------------------------------------------**

**2_Nematostella_vectensis EDNYRDVAWLGTTD------------------------------------------------------------------------------------------------------------------------------------------------------------------------------DGCLALAELLG-------------------------------------------------------------------------------------------------------------------------------------------------**

**2_Branchiostoma ENNYRDVAWLGDCD------------------------------------------------------------------------------------------------------------------------------------------------------------------------------EGCKALAELLG-------------------------------------------------------------------------------------------------------------------------------------------------**

**2_Strongylocentrotus DDKYRDVAYIGPCD------------------------------------------------------------------------------------------------------------------------------------------------------------------------------EGCEKLAGFVG-------------------------------------------------------------------------------------------------------------------------------------------------**

**2_Ciona_intestinalis DKAYRDVCWLGDCD------------------------------------------------------------------------------------------------------------------------------------------------------------------------------DGCLALADLLG-------------------------------------------------------------------------------------------------------------------------------------------------**

**2_Brugia_malayi KDNYRDVFWQGTTD------------------------------------------------------------------------------------------------------------------------------------------------------------------------------DGAWKLAELLG-------------------------------------------------------------------------------------------------------------------------------------------------**

**2_aSchistosoma_japonicum PGNKRDVFWSGNTD------------------------------------------------------------------------------------------------------------------------------------------------------------------------------DGVVKISELVG-------------------------------------------------------------------------------------------------------------------------------------------------**

**2_Schistosoma_mansoni SDNKRDIFWSGNAD------------------------------------------------------------------------------------------------------------------------------------------------------------------------------DGVVKISELLG-------------------------------------------------------------------------------------------------------------------------------------------------**

**2_aCaligus_clemensi DRRYRDVAEIGKCD------------------------------------------------------------------------------------------------------------------------------------------------------------------------------DGCKKLAEALG-------------------------------------------------------------------------------------------------------------------------------------------------**

**2_Lepeophtheirus DRRYRDVAEIGTCD------------------------------------------------------------------------------------------------------------------------------------------------------------------------------DGCKKLAEALG-------------------------------------------------------------------------------------------------------------------------------------------------**

**2_Aedes_aegypti PGNRRDVAWTGDCD------------------------------------------------------------------------------------------------------------------------------------------------------------------------------DGCFFLADKLG-------------------------------------------------------------------------------------------------------------------------------------------------**

**2_Apis_mellifera SNGARDVAWLGECD------------------------------------------------------------------------------------------------------------------------------------------------------------------------------IGCQLLADKLG-------------------------------------------------------------------------------------------------------------------------------------------------**

**2_Bombyx_mori EGSYRDVARLGDCD------------------------------------------------------------------------------------------------------------------------------------------------------------------------------EGCQDLADRLG-------------------------------------------------------------------------------------------------------------------------------------------------**

**2_Drosophila PNNTRDVAFLGDCD------------------------------------------------------------------------------------------------------------------------------------------------------------------------------AGVMALAKALG-------------------------------------------------------------------------------------------------------------------------------------------------**

**2_Pediculus DKNYRDVAFLGDCD------------------------------------------------------------------------------------------------------------------------------------------------------------------------------ELCEQLVDLLG-------------------------------------------------------------------------------------------------------------------------------------------------**

**2_Tribolium_castaneum KNNTRDVAWIGDCD------------------------------------------------------------------------------------------------------------------------------------------------------------------------------EGCQLLADKLG-------------------------------------------------------------------------------------------------------------------------------------------------**

**2_Danio_rerio DKAYRDVAHLSTCD------------------------------------------------------------------------------------------------------------------------------------------------------------------------------DGCMTLAELLG-------------------------------------------------------------------------------------------------------------------------------------------------**

**2_Salmo_salar --------------------------------------------------------------------------------------------------------------------------------------------------------------------------------------------------------------------------------------------------------------------------------------------------------------------------------------------------------**

**2_Xenopus_laevis EKAYRDVAWLGDCD------------------------------------------------------------------------------------------------------------------------------------------------------------------------------DGCLALADFLG-------------------------------------------------------------------------------------------------------------------------------------------------**

**2_Gallus_gallus DKAYRDVAWLGDCD------------------------------------------------------------------------------------------------------------------------------------------------------------------------------EGCLALAELLG-------------------------------------------------------------------------------------------------------------------------------------------------**

**2_Bostaurus KKAYRDVAWLGDCD------------------------------------------------------------------------------------------------------------------------------------------------------------------------------QGCLALADLLG-------------------------------------------------------------------------------------------------------------------------------------------------**

**2_Canis_familiaris KKAYRDVAWLGDCD------------------------------------------------------------------------------------------------------------------------------------------------------------------------------QGCLALADLLG-------------------------------------------------------------------------------------------------------------------------------------------------**

**2_Equuscaballus KKAYRDVAWLGDCD------------------------------------------------------------------------------------------------------------------------------------------------------------------------------QGCL-LADLLG-------------------------------------------------------------------------------------------------------------------------------------------------**

**2_Caligus_rogercresseyi ERRYRDVAEIGSCD------------------------------------------------------------------------------------------------------------------------------------------------------------------------------AGCSKLAEALG-------------------------------------------------------------------------------------------------------------------------------------------------**

**2_Rattusnorvegicus KKAYRDVAWLGDCD------------------------------------------------------------------------------------------------------------------------------------------------------------------------------QGCLALADLLG-------------------------------------------------------------------------------------------------------------------------------------------------**

**2_Musmusculus KKAYRDVAWLGDCD------------------------------------------------------------------------------------------------------------------------------------------------------------------------------QGCLALADLLG-------------------------------------------------------------------------------------------------------------------------------------------------**

**2_Susscrofa KKAYRDVAWLGDCD------------------------------------------------------------------------------------------------------------------------------------------------------------------------------QGCLALADLLG-------------------------------------------------------------------------------------------------------------------------------------------------**

**2_HUMAN KKAYRDVAWLGECD------------------------------------------------------------------------------------------------------------------------------------------------------------------------------QGCLALAELLG-------------------------------------------------------------------------------------------------------------------------------------------------**

**3_Ciona_intestinalis -----DLAVTGDIM------------------------------------------------------------------------------------------------------------------------------------------------------------------------------DSIQTFVDELG-------------------------------------------------------------------------------------------------------------------------------------------------**

**3_Xenopus_tropicalis -----DVAELGDLC------------------------------------------------------------------------------------------------------------------------------------------------------------------------------DIIHTMVSRLS-------------------------------------------------------------------------------------------------------------------------------------------------**

**3_Danio_rerioB -----DYMELGDLS------------------------------------------------------------------------------------------------------------------------------------------------------------------------------ESVRKLAEILG-------------------------------------------------------------------------------------------------------------------------------------------------**

**3_Gallus_gallus -----DIAQLGDVV------------------------------------------------------------------------------------------------------------------------------------------------------------------------------TGVEKMVELLD-------------------------------------------------------------------------------------------------------------------------------------------------**

**3_Taeniopygia_guttata -----DVAQLGDVV------------------------------------------------------------------------------------------------------------------------------------------------------------------------------SGVEKLVELLG-------------------------------------------------------------------------------------------------------------------------------------------------**

**3_Equus_caballus -----DVVQLGDVV------------------------------------------------------------------------------------------------------------------------------------------------------------------------------HGVEKLVELLG-------------------------------------------------------------------------------------------------------------------------------------------------**

**3_iso9Canis_familiaris -----DVVQLGDVV------------------------------------------------------------------------------------------------------------------------------------------------------------------------------HSVERLVELLG-------------------------------------------------------------------------------------------------------------------------------------------------**

**3_Mus_musculus -----DVVQLGDVV------------------------------------------------------------------------------------------------------------------------------------------------------------------------------HGVERLVDLLG-------------------------------------------------------------------------------------------------------------------------------------------------**

**3_aRattus_norvegicus -----DVVQLGDVV------------------------------------------------------------------------------------------------------------------------------------------------------------------------------QGVERLVDLLG-------------------------------------------------------------------------------------------------------------------------------------------------**

**3_Oryctolagus_cuniculus -----DVAQLGDVV------------------------------------------------------------------------------------------------------------------------------------------------------------------------------HGVGKLVELLG-------------------------------------------------------------------------------------------------------------------------------------------------**

**3_Monodelphis_domestica -----DVAQLGDII------------------------------------------------------------------------------------------------------------------------------------------------------------------------------GGVEMLVKALG-------------------------------------------------------------------------------------------------------------------------------------------------**

**3_Macaca_mulatta -----DVAKQLFCI------------------------------------------------------------------------------------------------------------------------------------------------------------------------------CV----------------------------------------------------------------------------------------------------------------------------------------------------------**

**3_Sus_scrofa -----DVVQLGDLV------------------------------------------------------------------------------------------------------------------------------------------------------------------------------HGVKRLVELLG-------------------------------------------------------------------------------------------------------------------------------------------------**

**3_HUMAN -----DVAQLGDVV------------------------------------------------------------------------------------------------------------------------------------------------------------------------------HGVESLVELLG-------------------------------------------------------------------------------------------------------------------------------------------------**

**4_Stigmatella_DW4/3 --------------------------------------------------------------------------------------------------------------------------------------------------------------------------------------------------------------------------------------------------------------------------------------------------------------------------------------------------------**

**4_Strongylocentrotus --------------------------------------------------------------------------------------------------------------------------------------------------------------------------------------------------------------------------------------------------------------------------------------------------------------------------------------------------------**

**4_Acyrthosiphon_pisum --------------------------------------------------------------------------------------------------------------------------------------------------------------------------------------------------------------------------------------------------------------------------------------------------------------------------------------------------------**

**4_Apis_mellifera --------------------------------------------------------------------------------------------------------------------------------------------------------------------------------------------------------------------------------------------------------------------------------------------------------------------------------------------------------**

**4_Drosophila VS------------------------------------------------------------------------------------------------------------------------------------------------------------------------------------------------------------------------------------------------------------------------------------------------------------------------------------------------------**

**4_Tribolium_castaneum --------------------------------------------------------------------------------------------------------------------------------------------------------------------------------------------------------------------------------------------------------------------------------------------------------------------------------------------------------**

**4_Danio_rerio --------------------------------------------------------------------------------------------------------------------------------------------------------------------------------------------------------------------------------------------------------------------------------------------------------------------------------------------------------**

**4_Xenopus_tropicalis EIQS----------------------------------------------------------------------------------------------------------------------------------------------------------------------------------------------------------------------------------------------------------------------------------------------------------------------------------------------------**

**4_Gallus_gallus --------------------------------------------------------------------------------------------------------------------------------------------------------------------------------------------------------------------------------------------------------------------------------------------------------------------------------------------------------**

**4_Taeniopygia_guttata --------------------------------------------------------------------------------------------------------------------------------------------------------------------------------------------------------------------------------------------------------------------------------------------------------------------------------------------------------**

**4_MOUSE VQRLEMNFPLSSAAQDP---------------------------------------------------------------------------------------------------------------------------------------------------------------------------------------------------------------------------------------------------------------------------------------------------------------------------------------**

**4_Bos_taurus --------------------------------------------------------------------------------------------------------------------------------------------------------------------------------------------------------------------------------------------------------------------------------------------------------------------------------------------------------**

**4_Equus_caballus --------------------------------------------------------------------------------------------------------------------------------------------------------------------------------------------------------------------------------------------------------------------------------------------------------------------------------------------------------**

**4_Pan_troglodytes --------------------------------------------------------------------------------------------------------------------------------------------------------------------------------------------------------------------------------------------------------------------------------------------------------------------------------------------------------**

**4_Canis_familiaris --------------------------------------------------------------------------------------------------------------------------------------------------------------------------------------------------------------------------------------------------------------------------------------------------------------------------------------------------------**

**4_Ovis_aries --------------------------------------------------------------------------------------------------------------------------------------------------------------------------------------------------------------------------------------------------------------------------------------------------------------------------------------------------------**

**4_Macaca_mulatta --------------------------------------------------------------------------------------------------------------------------------------------------------------------------------------------------------------------------------------------------------------------------------------------------------------------------------------------------------**

**4_Mus_musculus VQRLEMNFPLSSAAQDP---------------------------------------------------------------------------------------------------------------------------------------------------------------------------------------------------------------------------------------------------------------------------------------------------------------------------------------**

**4_Rattus_norvegicus --------------------------------------------------------------------------------------------------------------------------------------------------------------------------------------------------------------------------------------------------------------------------------------------------------------------------------------------------------**

**4_Sus_scrofa --------------------------------------------------------------------------------------------------------------------------------------------------------------------------------------------------------------------------------------------------------------------------------------------------------------------------------------------------------**

**4_HUMAN --------------------------------------------------------------------------------------------------------------------------------------------------------------------------------------------------------------------------------------------------------------------------------------------------------------------------------------------------------**

**5_Perkinsus_ATCC --------------------------------------------------------------------------------------------------------------------------------------------------------------------------------------------------------------------------------------------------------------------------------------------------------------------------------------------------------**

**5_Ciona_intestinalis --------------------------------------------------------------------------------------------------------------------------------------------------------------------------------------------------------------------------------------------------------------------------------------------------------------------------------------------------------**

**5_Apis_mellifera --------------------------------------------------------------------------------------------------------------------------------------------------------------------------------------------------------------------------------------------------------------------------------------------------------------------------------------------------------**

**5_Nasonia_vitripennis --------------------------------------------------------------------------------------------------------------------------------------------------------------------------------------------------------------------------------------------------------------------------------------------------------------------------------------------------------**

**5_Anoplopoma_fimbria TRVLDISCTQTTLSNTGGLYWYKYQKYLVDYNEQCGDGVLDK--------------------------------------------------------------------------------------------------------------------------------------------------------------------------------------------------------------------------------------------------------------------------------------------------------------**

**5_Danio_rerio V-------------------------------------------------------------------------------------------------------------------------------------------------------------------------------------------------------------------------------------------------------------------------------------------------------------------------------------------------------**

**5_Salmo_salar --------------------------------------------------------------------------------------------------------------------------------------------------------------------------------------------------------------------------------------------------------------------------------------------------------------------------------------------------------**

**5_Taeniopygia_guttata IS------------------------------------------------------------------------------------------------------------------------------------------------------------------------------------------------------------------------------------------------------------------------------------------------------------------------------------------------------**

**5_Bos_taurus VS------------------------------------------------------------------------------------------------------------------------------------------------------------------------------------------------------------------------------------------------------------------------------------------------------------------------------------------------------**

**5_Canis_familiaris VS------------------------------------------------------------------------------------------------------------------------------------------------------------------------------------------------------------------------------------------------------------------------------------------------------------------------------------------------------**

**5_Pan_troglodytes VS------------------------------------------------------------------------------------------------------------------------------------------------------------------------------------------------------------------------------------------------------------------------------------------------------------------------------------------------------**

**5_Macaca_mulatta VS------------------------------------------------------------------------------------------------------------------------------------------------------------------------------------------------------------------------------------------------------------------------------------------------------------------------------------------------------**

**5_Mus_musculus TS------------------------------------------------------------------------------------------------------------------------------------------------------------------------------------------------------------------------------------------------------------------------------------------------------------------------------------------------------**

**5_Ornithorhynchus VS------------------------------------------------------------------------------------------------------------------------------------------------------------------------------------------------------------------------------------------------------------------------------------------------------------------------------------------------------**

**5_Rattus_norvegicus IS------------------------------------------------------------------------------------------------------------------------------------------------------------------------------------------------------------------------------------------------------------------------------------------------------------------------------------------------------**

**5_Sus_scrofa VS------------------------------------------------------------------------------------------------------------------------------------------------------------------------------------------------------------------------------------------------------------------------------------------------------------------------------------------------------**

**5_HUMAN VS------------------------------------------------------------------------------------------------------------------------------------------------------------------------------------------------------------------------------------------------------------------------------------------------------------------------------------------------------**

SIRT6VITIS PFVRIDLFQVILTHTLSSDKRFV NWILR-----------------------------------------------VASV--HGQKAPLPF--------------------------------IKYVEVSFLDGQNYK**----**EA**------------------------**VLHK--QPFQLKRRTVKTKIFEVLLKLNFSDGCGCLSSQIKVPIDFKVSTDCFN--YDKDAILQKLRDTATGDPCCGRHEVIEKKPIPDPRSEATVYAIVTNVLQYN-KTAPESNGSVMKGRLGGLNGIE-----TSWKRSRSGK-------------------------------

SIRT6RICINUS PYVRIDLLQIIVTRSLSADKRFV NWTLR-----------------------------------------------IASV--HALKATLPF--------------------------------IKSIEVTFSDTQKYK**----**AA**------------------------**ILHE--QPFNLKRRTVTTESFEIFLKLNLSDGCGCLCTQINIPFGFKVLNDCFN--LKKDSVIQNLREKAIQVLGCGQNAMIERKTIIAPRSEVTVHAIVTNIKAFE-SDG-LSNGEVKRLRGSSINGIM-----TCRKRSNSRK-------------------------------

SIRT6ARAB PYVRIDLFQIILTQSISGDQRFI NWTLR-----------------------------------------------VASV--HGLTSQLPF--------------------------------IKSIEVSFSDNHNYK**----**DA**------------------------**VLDK--QPFLMKRRTARNETFDIFFKVNYSDGCDCVSTQLSLPFEFKISTEEHVEIIDKEAVLQSLREKAVEESSCGQSGVVERRVVSEPRSEAVVYATVTSLRTYHSQQSLLANGDLKWK----LEGSG-----TSRKRSRTGK-------------------------------

SIRT6ZEAMAYS PYIRTDFVQLTLRHSLK--KKCV RWTLR-----------------------------------------------VTSI--HGLRAPLPF--------------------------------LQSVKVSFPERPDLK**----**SV**------------------------**VLKE--QPFSLQRETSMNKPFFMLLTLNFSDGCSCLSSSIGWPVDFQKRKDSFV--RDRALVLRELYSAAQRESCIGQQEILERENL--PRAETSIHGIVTNIVRYDTEDEKLAPPKNDLMNHSRSNPAKRHVEGTDCHSSLPKK-------------------------------

SIRT6TRITICUM PYIRTDFIQLLLRHTVK--KKCV RWTLR-----------------------------------------------VTSV--HGMRAPLSF--------------------------------LRSIEVSFPDRSDMK**--------**PVVLME--QPFSLQRETSMTSIFSMLLTLKFSDGCGNH----------------------------------------------------------------------------------------------------------------------------------------------------------------

SIRT6ORIZA PYIRTDFVQISLRNSVK--KKCV RWTLR-----------------------------------------------VTSI--HGLRAPLPF--------------------------------LRSVEVSFPERPDMKPVVLKE**------------------------**--QPFSLQRETSMNRPFVMLLTFNFSDGCGCSSSSIEWPVDFLKQKDSFV--RDRSLVLQELQHAAEHRSRAGQHAILEREGV--PRAETSIHALVTNIVRYDTEDSKAAVPMATWMN-SNGSLSKRHMDAIGCNPASSKK-----------------------------------

SIRT6PHYSCOM PYVHIDRILLSYYYYWTK-KKSV KWYFR-----------------------------------------------ISSI--HGQKMALPF--------------------------------IKSIEVMFPNRPEFK**----**PA**------------------------**AFAK--PPCLVRRETMRLKELDVALKLHFAEGCMCSSGDIFQTLSFEVRDNYLLCRID----------------------------------------------------------------------------------------------------------------------

**6_Strongylocentrotus PEYTGPSLVLESQQGLSTKNIKDTMHVGDSQKNCKVDSECVDDRRKVAVKDEVKEESMNDGQKVEIKDEVKEKVQKERVSEDLEVSPEMKR-------------------------------EAHLNSHETPDQKVEQSCIRLE------------------------SIEDRQKVAVKDEVKESCLSDGQKVETKDEVKEKVQKERVSEDLEVPPEVKSEAHLDSHETPQENSEQSCKRLCPDTSEVIQDDVAKDTEKHEASDNSFRTCKHEDDDMRETVDIQETSCLVGSDSKQTFENMCSVKQEIHVKSGSADTTDIKPGCPDNWSDSKRTDDEESVENIT**

**6_Lepeophtheirus PEYDKEIDPIRMMD--------------------------------------------------------------KSKDP-NFFIDWTQS-------------------------------EKEAKKIIIKSDRLEDALKMKR------------------------KKDREVTAKLASKKNKSLCDDEEEKNNRGEVLNNIFILKKEETDHCNGENASSFELKNEKDL------------------------------------------------------------------------------------------------------------------**

**6_Acyrthosiphon_pisum --------------------------------------------------------------------------------------------------------------------------------------------------------------------------------------------------------------------------------------------------------------------------------------------------------------------------------------------------------**

**6_Apis_mellifera PEYESTMDPTRNSDTTS-----------------------------------------------------------KEMDW-TIPTSRIKE-------------------------------MNVLYKKVCKPMRRKRKTFMYE------------------------RERTDTKRETKTKKQAFMIKQDIKTEDTMNTANQICNNAVVSEDISSNTVKIEDEIKHVEPFEFTTNNMTQPDPGLEVNNIL----------------------------------------------------------------------------------------------**

**6_Drosophila PEYSEASDPTKQS---------------------------------------------------------------KPMEW-TIPTSNVNT-------------------------------FHRQYKKYVY--------FIY-----------------------------------------YLL-------------------------------------------------------------------------------------------------------------------------------------------------------------**

**6_Danio_rerio PEWAGPTLCEDSGGDLD----------------------------------------------------------ILPYG---AWKKEVKI-------------------------------ELKIEESKHTVSKKRK------------------------------RKEQHAEEDYKNGVKVEEEMKEEGKESDSHVHTHT---------------------------------------------------------------------------------------------------------------------------------------------**

**6_Xenopus_tropicalis PVWTG-------MPTKT-----------------------------------------------------------EPTN----GNYKEEN-------------------------------HFYNDSVLGANPNQKR-------------------------------------------EGCKEEPNLEPKKAKVEPACV----------------------------------------------------------------------------------------------------------------------------------------------**

**6_Gallus_gallus PEWTGPVVVESADSAKPEQ-----------------------------------------------------LYTFKPEA---HGLLKEEP-------------------------------FSQHNGTAGQCPDLGT-------------------------------TLVEHRDSLKQECPSPDTGPPLTKKMKVEPLLT----------------------------------------------------------------------------------------------------------------------------------------------**

**6_Bos_taurus PAWDGPHMVERALPPLP-----------------------------------------------------------RPPA--PKLEPKEEA-------------------------------SPQLNSPVPANPKQEPTAEPCT------------------------QHNGSGPTSPKRERPDSPSPHRPPKRVKTEVVPS----------------------------------------------------------------------------------------------------------------------------------------------**

**6_Canis_familiaris PAWDGPRVLERALPPLP-----------------------------------------------------------RPPAKPPEPEPKEEA-------------------------------PAQLNGPAPASPKQEPSTEPCT------------------------QHNGSGPGSPKRERLDSPVPHRPPKRVKAEVAPS----------------------------------------------------------------------------------------------------------------------------------------------**

**6_Mus_musculus PAWDGPCVLDKALPPLP-----------------------------------------------------------RPVA------LKAEP-------------------------------PVHLNGAVHVSYKSKP---------------------------------------------NSPILHRPPKRVKTEAAPS----------------------------------------------------------------------------------------------------------------------------------------------**

**6_Pan_troglodytes PAWDGPRVLERALPPLP-----------------------------------------------------------RPPT--PKLEPKEES-------------------------------PTRINGSIPAGPKQEP----CA------------------------QHNGSEPASPKRERPTSPAPHRPPKRVKAEAVPS----------------------------------------------------------------------------------------------------------------------------------------------**

**6_Sus_scrofa PAWDGPRVLERALPPLP-----------------------------------------------------------RPPA--PKLEPKEEA-------------------------------PAQFNCPAPASTKQEPKTEPCA------------------------QHNGSGPTSPKREQLDSPAPHKPPKRAKAEVIPS----------------------------------------------------------------------------------------------------------------------------------------------**

**6_Macaca_mulatta PAWDGPHVLERALPPLP-----------------------------------------------------------RPPT--PKLEPKEES-------------------------------PTRINGSIPAGPKQEP----CA------------------------QHNGSEPASPKRERPTSPAPNRPPKRVKAEAVPS----------------------------------------------------------------------------------------------------------------------------------------------**

**6_Rattus_norvegicus PTWDGPRVLEKALPPLP-----------------------------------------------------------RPVA------PKAEP-------------------------------PVHLNG----SYKPKP---------------------------------------------DSPVPHRPPKRVKTEAAAS----------------------------------------------------------------------------------------------------------------------------------------------**

**6_HUMAN PAWDGPRVLERALPPLP-----------------------------------------------------------RPPT--PKLEPKEES-------------------------------PTRINGSIPAGPKQEP----CA------------------------QHNGSEPASPKRERPTSPAPHRPPKRVKAKAVPS----------------------------------------------------------------------------------------------------------------------------------------------**

SIRT7PHYSCOM E------QNPSLVSS--------------------------------------------------------------NRALEHDVGVHEDM-------------------------------SSECYDQKLEEFERSFMVQTT--------------------------------------SFAP---------RT----------------------------------------------------------------------------------------------------------------------------------------------------

**7_Ciona_intestinalis AVYFQHTDSLWGLARQPKLNELSTFTTTSIT---------------------------------------------YPISTAHQSSLSEQSNIINTHVCSANRTASWPGPALIPQPIESPNFLSDTNRENVLDEKPYLVPAVTHSQYPSASSQVTGQSVVSVADMSSKVEQQTLSQQCVVSPAPSWFGKGYRSKVNKKNGFKKRGKRKLS--------------------------------------------------------------------------------------------------------------------------------------**

**7_Apis_mellifera PQYNRAKDPIFFHAV--------------------------------------------------------------RLRNNEQYTTSQPC-------------------------------LEEKR-------------------------------------------------------------MHFTQKSN----------------------------------------------------------------------------------------------------------------------------------------------------**

**7_Danio_rerio PVYSRLQDPIFSMAK--------------------------------------------------------------PLSPQEQKSHSRKE-------------------------------IAPPSALEEVSQSAPPQGEGPA-------------------------------------VQGGWFGRGYSKGRRKK---------KSS--------------------------------------------------------------------------------------------------------------------------------------**

**7_Xenopus_tropicalis PVYDRSQDPIFSLAV--------------------------------------------------------------PLHSSEEQSHTRKP-------------------------------IKTADSHNIIPEQEQPVVEQP--------------------------------------VSGGWFGKGYTKGRRMR---------R----------------------------------------------------------------------------------------------------------------------------------------**

**7_Bos_taurus PRYSRWQDPIFSLAT--------------------------------------------------------------PLRAGEEGSHSRKS-------------------------------LCRSREEPGPGDRGAPLSSAP--------------------------------------ILGGWFGRGCTKRTKRK---------KVT--------------------------------------------------------------------------------------------------------------------------------------**

**7_Canis_familiaris PPYSRWQDPIFTLAT--------------------------------------------------------------PLRAGEEGSHSRKS-------------------------------LCRSREDPPPGDRGAALSSAP--------------------------------------VLGGWFGRGCAKRTKRR---------KIT--------------------------------------------------------------------------------------------------------------------------------------**

**7_Equus_caballus PPYSRWQDPIFSLAT--------------------------------------------------------------PLRAGEEGSHSRKS-------------------------------LCRSREEPPPGDRGSPLSSAP--------------------------------------VLGGWFGRGCAKRTKRK---------KVL--------------------------------------------------------------------------------------------------------------------------------------**

**7_Macaca_mulatta PAYSRWQDPIFSLAT--------------------------------------------------------------PLRAGEEGSHSRKS-------------------------------LCRSREEAPPGDRGAPLSSAP--------------------------------------ILGGWFGRGCTKRTKKK---------KVT--------------------------------------------------------------------------------------------------------------------------------------**

**7_Mus_musculus PVYNRWQDPIFSLAT--------------------------------------------------------------PLRAGEEGSHSRKS-------------------------------LCRSREEAPPGDQSDPLASAPP-------------------------------------ILGGWFGRGCAKRAKRK---------KVA--------------------------------------------------------------------------------------------------------------------------------------**

**7_Pan_troglodytes PAYSRWQDPIFSLAT--------------------------------------------------------------PLRAGEEGSHSRKS-------------------------------LCRSREEAPPGDRGAPLSSAP--------------------------------------ILGGWFGRGCTKRTKRK---------KVT--------------------------------------------------------------------------------------------------------------------------------------**

**7_aRattus_norvegicus PVYNRWQDPIFSLAT--------------------------------------------------------------PLRAGEEGSHSRKS-------------------------------LCRSREEPPPGDQSAPLASATP-------------------------------------ILGGWFGRGCAKRAKRK---------KAA--------------------------------------------------------------------------------------------------------------------------------------**

**7_Sus_scrofa PPYSRWQDPIFSLAT--------------------------------------------------------------PLRAGEEGSHSRKS-------------------------------LCRSREEPGPEDRGAPRSSAP--------------------------------------ALGGWFGRGCTKRTKRK---------KVT--------------------------------------------------------------------------------------------------------------------------------------**

**7_HUMAN PAYSRWQDPIFSLAT--------------------------------------------------------------PLRAGEEGSHSRKS-------------------------------LCRSREEAPPGDRGAPLSSAP--------------------------------------ILGGWFGRGCTKRTKRK---------KVT--------------------------------------------------------------------------------------------------------------------------------------**

**B (SECOND PART: continuing from the precedent to the end (at right)**

**1-Hydra_magnipapillata ------------------------------------------QSDSPQIDEG-----------------------------------------------------------------------------------------------------------------------------IYFLPIPPNKFVFHGAELYTEDCP----------------------------------------------------------------------------------------------------------------------------------------------**

**1-Strongylocentrotus AQRNSAAEQYMLFQRTLDSIGNSSTGASSLDVMAGNDSVASGESESTQTHVASDC----------------------------------------KEPSTDVKAEAEDLGLCVNETDRTNSQDDLNSEVGNVSSSSGSVLEHREQASPAPDSEAKPSPSTSAAAKTPRATISTQLKESSFLFIPPMRYVFHGAEVFLSDDDNEIEHGLEGLNKDMDINDLHNELENGDLHNDLGSGDLHNELENGDLHNHLHNGDDLPDLSEEDHSPAKEKTVSLDDLTPTVPSTDRETSQYGQSASKAQCVPETNGRDGSPFEAPSGETVYTHIDLPVTP------------**

**1-Brugia_malayi ---------------------------------------------------------------------------------------------------------------------------------------------------------------MPSMWDARYISVGSKLPVDGYLFIVPNKNIFPGAEIYYDKDDDIFRQLPEHYHSSAASSSSDSGESVTNQEEIYCNVGVRTMSLEESSPARAGSESDERSSSCPPKMDLETKYSAESSCAGNRLSVRLEPFLQTITVQTREETTHGLVRTSTSNHLLL--------------------------**

**1-aSchistosoma_mansoni --------------------------------------------------------------------------------------------------------------------------------------------------------------------EKTYTSTVVSSSDNSSELMNKNSSNDAVIEVSEDEEDGECVWEVASSLPR---------------------------------------------------------------------------------------------------------------------------------**

**1-Schistosoma_japonicum -------------------------------------------------------------------------------------------------------------------------------------------------------------------------------------------------------------------------------------------------------------------------------------------------------------------------------------------------------**

**1-Acyrthosiphon_pisum NLSTD-----------------------------------STRDSGIDPDD------------------------------------------------------------------------------------------------------------------PQKSSLAAYLPSNKYYMLKKRRYMFSGAEVD------------LNDMNDD---------------SETESDSSEKSETPPLH------------------------------------------------------------------------------------------------**

**1-Tribolium_castaneum HMSVD-----------------------------------SARDSGIGDNS--------------------------------------------NFTDLETKYDDTSDENPDLGEYNTNTNTASDPQFTNISNDEANKTNTYLESTTNTSDLKG------FWQPKIKKSLAERLPPKSFYLVKPSRYIFPGAEIYYDPDE------KFGYYEGS---------------SSSNNSDSESENGEPVSN-----------------------------------------------------------------------------------------------**

**1-Apis_mellifera EIISE-----------------------------------SSIDYKFHTVSVESTSKDIGKIYSLEECQVFPRIIEISSESALLDSTLKPHHCVENRTSLKMNNDYSTMNSTEIEKTNFKPRQASIDSALDSGVGDSCNSVDSHEDKNSKEELKNGTLNQHCWHPKIRKSLAERLPENSYYQLAPGKYIFPGAEVYSDPEEYDHCSLSINSESSD---------------SDSDSSSIDEEEDDEVEEEEEEEEEEEQDEENERNENQVEDNDDKLKEKLGMKKEGHEEEINEKNIKEDVKGHRKIETKNNKIETENNQRKIMEREDERRNISKFHNENILEN**

**1-Pediculus NVITQ-----------------------------------SLRDVRV--------------------------------------------------------------------------------------------------------------------------------------------------------------------------------------------------------------------------------------------------------------------------------------------------------**

**1-Danio_rerio DHANA------------------------------------EHTENTSAGH----------------------------------------------------------------------------------------------------------VNAEHIEHMSKD----HANPK----DDQSSLSVNEEELASPAAET--HALDSTEISAH---------------TERS-----KEADAVNTDDAACVK-------DEE-NTDRLR-----------VEMRRR----CWRSRICQSPISKRLG-----------------------------------**

**1-aNothobranchius_f VEMGD------------------------------------FKSQNSSFDY----------------------------------------------------------------------------------------------------------RKRYWVSRISRSPISKRLNPGQYLFQSPNRYLFHGAEVYSDSEEE--TSSSCGSDSDE---------------SECSPGGVEDDSDPEDVSEAAAAD-------GETRIKDMFR-----------NSANEE----ESSVQIDSSSEKAHSATNQA-------------------------------**

**1-bNothobranchius_k VEMGD------------------------------------FKSQNSSFDY----------------------------------------------------------------------------------------------------------RKRYWVSGISRSPISKRLNPGQYLFQSPNRYLFHGAEVYSDSEEE--TSSSCGSDSDE---------------SECSPGGVEDDSDPEDVSEAAAAD-------GETRIKDMFR-----------NSANEE----KSSVQIDSSSEKAHSATNQA-------------------------------**

**1-Xenopus_tropicalis DQETA------------------------------------EKDTDIDSAK-------------------------------------------------------------------------------------------------------------DLESKYTKEQISKRLDSTQFLFLAPNRYIFHGAEVFSDSDED-LTSSSCGTNSDS---------------ESLLSPSLHEPIEEDSDTEECFHAK------YENETDTDNR-----------ADLERE--PERVVLYQSDDLLGIDGTTMNL-------------------------------**

**1-Taeniopygia_guttata MNSET------------------------------------MKENGSNDGE------------------------------------------------------------------------------------------------NKEKS---EILKKCWVNRSAKEQISKRLDGTQYLFLPPNRYIFHGAEVYSDSEDDIISSSSCGSSSES---------------GSCRSQSLD--VEDESEMEEFYNG-------IEDED-APER---EEEPGFGEDGAEQEELAAEESAETNEAAGTEHP-SNAL-------------------------------**

**1-Gallus_gallus MNSET------------------------------------MKENGTNNGE------------------------------------------------------------------------------------------------SKEKN---EIVKKCWVNRSAKEQISKRLDGTQYLFLPPNRYIFHGAEVYSDSEDDMISSSSCGSSSES---------------GSCHSQSLD--VEDESEIEEFYNG-------IEDED-APER---EVEAAFEEDGVEQD--AADESAYTNEAAGNDHPTSNKL-------------------------------**

**1-Equus_caballus GSPY-------------------------------------LKNVGSDTGE------------------------------------------------------------------------------------------------KNERTSVAEAVRKCWPARLAKEQISKRLDGNQYLFLPPNRYIFHGAEVYSDSEEDVLSSSSCGSNSDS---------------GTCQSPSLEEPMEDESEIEEFYNG-------LEDDADVNER---AGGTGFRADGSDQE--AVNETISTKQEATDINYSSNKS-------------------------------**

**1-Macaca_mulatta GNPD-------------------------------------LKNVGSSTGE------------------------------------------------------------------------------------------------KNERTSVAGTVRKCWPSRVAKEQISKRLDGNQYLFLPPNRYIFHGAEVYSDSEDDVLSSSSCGSNSDS---------------GTCQSPSLEEPMEDESEIEEFYNG-------LEDEPDIPER---AGGAGFGTDGDDQE--AINEAISMKQEVTDMNYPSNKS-------------------------------**

**1-Monodelphis_domestica E------------------------------------------DAGANGGE------------------------------------------------------------------------------------------------KNERTNVVETLRKCWPNRLAKEQISKRLDGNQYLFLPPNRYIFHGAEVYSDSEDDVLSSSSCGSNSDS---------------GTCRSPSLEEPMEDESEMEEFYNG-------LEEAD-GPERGGPACAC----DKEDQR--AVGEAASITDEAAAIDHPSSRL-------------------------------**

**1-Ornithorhynchus EGLDR------------------------------------TKDAAASIGENKE---------------------------------------------------------------------------------------------RNERTSAAETLRKCWGSRLAKEQISKRLDGNQYLFLPPNRYIFHGAEVYSDSEDDILSSSSCGSNSDS---------------GTCHSPSLEEPLEDESEIEEFYNG-------LEDDAVGPERGGGGAAAGFGGDDDEQE--AVTEAVSVKVEAAEMDHSSNGL-------------------------------**

**1-Canis_familiaris ESPD-------------------------------------LKNVGCNTGE------------------------------------------------------------------------------------------------KNERTSVADPVRKCWPARLAKEQISKRLDGNQYLFLPPNRYIFHGAEVYSDSEDDVLSSSSCGSNSDS---------------GTCQSPSLEEHLEDESEIEEFYNG-------LEDEADVNER---AGGTGFGIDGGDQE--AVNEAISMKQEATDTNYPSNKS-------------------------------**

**1-Bos_taurus ESPD-------------------------------------LKNAVSNSGE------------------------------------------------------------------------------------------------KNERTSVAETVRKCWPARLAKEQISKRLDDNQYLFLPPNRYIFHGAEVYSDSEDDVLSSSSCGSNSDS---------------GTCQSPSLEEPMEDESENEEFYNG-------LEDDADVNER---AGGTVFEADGGDQE--AINEAISVKQEATCINYPSNKS-------------------------------**

**1-Sus_scrofa ESPG-------------------------------------LKNVACSNGE------------------------------------------------------------------------------------------------KNERTSVAETVRKCWPARLAKEQISKRLDGNQYLFLPPNRYIFHGAEVYSDSEDDVLSSSSCGSNSES---------------GTCQSPSLEEPMEDESEIEEFYNG-------LEDDADVNMR---AGGTGFGADGSDQE--AVNEAISMKQEATGVNYPSNKS-------------------------------**

**1-Rattus_norvegicus ENPD-------------------------------------FKAVGSSTGD------------------------------------------------------------------------------------------------KNERTSVAETVRKCWPNRLAKEQISKRLDGNQYLFVPPNRYIFHGAEVYSDSEDDALSSSSCGSNSDS---------------GTCQSPSLEEPLEDESEIEEFYNG-------LEDDADRPEC---AGGS--GADGGDQE--AVNEAIAMKQELTDVNCTPDKSEHY----------------------------**

**1-Mus_musculus ENPD-------------------------------------FKAVGSSTAD------------------------------------------------------------------------------------------------KNERTSVAETVRKCWPNRLAKEQISKRLEGNQYLFVPPNRYIFHGAEVYSDSEDDVLSSSSCGSNSDS---------------GTCQSPSLEEPLEDESEIEEFYNG-------LEDDTERPEC---AGGSGFGADGGDQE--VVNEAIATRQELTDVNYPSDKS-------------------------------**

**SIRT1_HUMAN ENPD-------------------------------------LKNVGSSTGE------------------------------------------------------------------------------------------------KNERTSVAGTVRKCWPNRVAKEQISRRLDGNQYLFLPPNRYIFHGAEVYSDSEDDVLSSSSCGSNSDS---------------GTCQSPSLEEPMEDESEIEEFYNG-------LEDEPDVPER---AGGAGFGTDGDDQE--AINEAISVKQEVTDMNYPSNKS-------------------------------**

**2_aAjellomyces_ER -----------------------------------------------------------------------------------------------------------------------------------------------------------------WREELEELWAQTNPEKAGLMEKEKEKPARSKNELLRDEIERLTEEVEHTLHISRAHEDRVRSELQKGKESEVGNGEDATDKREPTPTSQDDEDRVMKKGNEPSESEQLDPVPQQESKSETKSSDDCATKQENSSTAGREPNKESDQETER--------------------------------**

**2_aMicromonas_CCMP1545 -----------------------------------------------------------------------------------------------------------------------------------------------------------------WGDELDELVRAG-----------VEKVKSPTIDR----------------------------------------------------------------------------------------------------------------------------------------------------**

**2_Ostreococcus_tauri -----------------------------------------------------------------------------------------------------------------------------------------------------------------WKEDLDALIKSC-----------EIRNKLAQEAPAECLNG----------------------------------------------------------------------------------------------------------------------------------------------**

**2_Trichoplax_adhaerens -----------------------------------------------------------------------------------------------------------------------------------------------------------------WKEELLQLKERGHKILIEKYPQMVTKEAKVSLISSAPEQHSQAVESKSNADGNKESTEDEGSQNFDAIAVTQEHQIN---------------------------------------------------------------------------------------------------------**

**2_Nematostella_vectensis -----------------------------------------------------------------------------------------------------------------------------------------------------------------WKAS-------------EPFAIV---------------------------------------------------------------------------------------------------------------------------------------------------------------**

**2_Branchiostoma -----------------------------------------------------------------------------------------------------------------------------------------------------------------WKEQLEELVHREHTRIEAQ-------------------------------------------------------------------------------------------------------------------------------------------------------------------**

**2_Strongylocentrotus -----------------------------------------------------------------------------------------------------------------------------------------------------------------WKKEMTVLVNKGE---AKKKKKGETESPTKDETAKTPTSATKDQKPKTSASPGKKS---SASPTKDKKPSSKKD------------------------------------------------------------------------------------------------------------**

**2_Ciona_intestinalis -----------------------------------------------------------------------------------------------------------------------------------------------------------------WKNELKTMVCNQHKEIDQNSQATASKE-----KCPSKKSA----------------------------------------------------------------------------------------------------------------------------------------------**

**2_Brugia_malayi -----------------------------------------------------------------------------------------------------------------------------------------------------------------WKTELNKLIETELKKINEKXEKDEKNIKSTVTTIDNISINEEKNGKSIERXKSVDXKSNKL-------------------------------------------------------------------------------------------------------------------------**

**2_aSchistosoma_japonicum -----------------------------------------------------------------------------------------------------------------------------------------------------------------WKDDLLKLKEETDSR---------LIAQFVEKKSQQ--------------------------------------------------------------------------------------------------------------------------------------------------**

**2_Schistosoma_mansoni -----------------------------------------------------------------------------------------------------------------------------------------------------------------WKDDLLRLKKETDSR---------LNEEFLAKKSQDKTNGQ---------------------------------------------------------------------------------------------------------------------------------------------**

**2_aCaligus_clemensi -----------------------------------------------------------------------------------------------------------------------------------------------------------------WKEELISLMGGS--------------------------------------------------------------------------------------------------------------------------------------------------------------------------**

**2_Caligus_rogercresseyi -----------------------------------------------------------------------------------------------------------------------------------------------------------------WKEELISLMGGS--------------------------------------------------------------------------------------------------------------------------------------------------------------------------**

**2_Lepeophtheirus -----------------------------------------------------------------------------------------------------------------------------------------------------------------WKEELESLMNK---------------------------------------------------------------------------------------------------------------------------------------------------------------------------**

**2_Aedes_aegypti -----------------------------------------------------------------------------------------------------------------------------------------------------------------MGDELREMIKREHAKLDAAQHKHFPVAEPNPTETASATASQNHDSDDVMIHDVQHAVDEPATNIAPVPDLVPVDINHHGEMKDLGEGKSTTVADGTKND-----------------------------------------------------------------------------------**

**2_Apis_mellifera -----------------------------------------------------------------------------------------------------------------------------------------------------------------WGDELKNLIKREYERLNR------G-------------------------------------------------------------------------------------------------------------------------------------------------------------**

**2_Bombyx_mori -----------------------------------------------------------------------------------------------------------------------------------------------------------------WGDELRALVAREHERLDQ------ELLTASPHAPVLIPSEANAGPSASE-------------------------------------------------------------------------------------------------------------------------------------**

**2_Drosophila -----------------------------------------------------------------------------------------------------------------------------------------------------------------WDQELQQLITSERKKLSGSQ----NSEELQQGKEKPQSDPDKMTSGDRDKKDASL-------------------------------------------------------------------------------------------------------------------------------**

**2_Pediculus -----------------------------------------------------------------------------------------------------------------------------------------------------------------WKQEFDEIIKKSDEQENY----------MKSKGELFKFDMSNMRNIV---------------------------------------------------------------------------------------------------------------------------------------**

**2_Tribolium_castaneum -----------------------------------------------------------------------------------------------------------------------------------------------------------------WGDELKKLRQTEIEKIEK--------AEATPVKSSM--------------------------------------------------------------------------------------------------------------------------------------------------**

**2_Danio_rerio -----------------------------------------------------------------------------------------------------------------------------------------------------------------WKKELEEMVKREHALIDS--KDAKKTD-----KEASQSSKSAVAEAEKTDKTE---------------------------------------------------------------------------------------------------------------------------------**

**2_Salmo_salar -------------------------------------------------------------------------------------------------------------------------------------------------------------------------------------------------------------------------------------------------------------------------------------------------------------------------------------------------------**

**2_Xenopus_laevis -----------------------------------------------------------------------------------------------------------------------------------------------------------------WKAELEELVKKEHAEIDAAAEAAKKKE-----SKPDNSSKEESSAANTSDDKANN-------------------------------------------------------------------------------------------------------------------------------**

**2_Gallus_gallus -----------------------------------------------------------------------------------------------------------------------------------------------------------------WKKELQELVRKEHAAIDAVAAPEDTSSASGGDPTSRRGRTDGSGGRAESSGASSEQRGDGKEP-----------------------------------------------------------------------------------------------------------------------**

**2_Bostaurus -----------------------------------------------------------------------------------------------------------------------------------------------------------------WKKELEDLVRKEHASIDAQSGSGASNP--ATSASPRNSPPPPTKEEPRTTEGEKPQ------------------------------------------------------------------------------------------------------------------------------**

**2_Canis_familiaris -----------------------------------------------------------------------------------------------------------------------------------------------------------------WKKELEDLVRKEHAHIDAQAGSGAPNPSTSTSASPSKSPPP-AKEEARTKEGEKPQ------------------------------------------------------------------------------------------------------------------------------**

**2_Equuscaballus -----------------------------------------------------------------------------------------------------------------------------------------------------------------WKTTHS-------------GATKVPPP--SLSVSPSKSPPP-AKEEARTTEREKPQ------------------------------------------------------------------------------------------------------------------------------**

**2_Rattusnorvegicus -----------------------------------------------------------------------------------------------------------------------------------------------------------------WKKELEDLVRREHANIDAQSGSQASNP--SATVSPRKSPPP-AKEAARTKEKEEH-------------------------------------------------------------------------------------------------------------------------------**

**2_Musmusculus -----------------------------------------------------------------------------------------------------------------------------------------------------------------WKKELEDLVRREHANIDAQSGSQAPNP--STTISPGKSPPP-AKEAARTKEKEEQQ------------------------------------------------------------------------------------------------------------------------------**

**2_Susscrofa -----------------------------------------------------------------------------------------------------------------------------------------------------------------WKKELEDLVRKEHASIDAQSGSGTPNP--TTSASPRKSPPP-AKAEARTSEGEKPQ------------------------------------------------------------------------------------------------------------------------------**

**2_HUMAN -----------------------------------------------------------------------------------------------------------------------------------------------------------------WKKELEDLVRREHASIDAQSGAGVPNP--STSASPKKSPPP-AKDEARTTEREKPQ------------------------------------------------------------------------------------------------------------------------------**

**3_Ciona_intestinalis -----------------------------------------------------------------------------------------------------------------------------------------------------------------WDEFIKEVVDSNEKVLHVNDPPHEDSGISSASSTSSERKVIPPVDKNVKRYFHSLRSTPLPNNNKETGSGEPPHSYYEFMKHSIQNPPPKSTSALSKQRPPNKQHPPVIQYTRTILSKSKSFPRMRSSISVPSLYRGNKVETTSESETSDASSSSGVDS-----------------------**

**3_Xenopus_tropicalis -----------------------------------------------------------------------------------------------------------------------------------------------------------------WQAELDQLMNS------------PTSGVCVMSSNGHMAWEKTNWV-----------------------------------------------------------------------------------------------------------------------------------------**

**3_Danio_rerioB -----------------------------------------------------------------------------------------------------------------------------------------------------------------WHTEIQTLMNS------------HENGLYSYISSS---------------------------------------------------------------------------------------------------------------------------------------------------**

**3_Gallus_gallus -----------------------------------------------------------------------------------------------------------------------------------------------------------------WNEEMQTLIQK-------------EKEKLDAKDK----------------------------------------------------------------------------------------------------------------------------------------------------**

**3_Taeniopygia_guttata -----------------------------------------------------------------------------------------------------------------------------------------------------------------WNKEMQTLIQK-------------EKEKLDAKDK----------------------------------------------------------------------------------------------------------------------------------------------------**

**3_Equus_caballus -----------------------------------------------------------------------------------------------------------------------------------------------------------------WTEEMQDLIQR-------------ETGKLDGQDR----------------------------------------------------------------------------------------------------------------------------------------------------**

**3_iso9Canis_familiaris -----------------------------------------------------------------------------------------------------------------------------------------------------------------WREELQDLIQQ-------------ETEKLDGRDG----------------------------------------------------------------------------------------------------------------------------------------------------**

**3_Mus_musculus -----------------------------------------------------------------------------------------------------------------------------------------------------------------WTQELLDLMQR-------------ERGKLDGQDR----------------------------------------------------------------------------------------------------------------------------------------------------**

**3_aRattus_norvegicus -----------------------------------------------------------------------------------------------------------------------------------------------------------------WTQELQDLIQR-------------ENGKLDGQDG----------------------------------------------------------------------------------------------------------------------------------------------------**

**3_Oryctolagus_cuniculus -----------------------------------------------------------------------------------------------------------------------------------------------------------------WTEEMHDLIQR-------------ETRKLDGRDT----------------------------------------------------------------------------------------------------------------------------------------------------**

**3_Monodelphis_domestica -----------------------------------------------------------------------------------------------------------------------------------------------------------------WTQEIEDLVQR-------------ETSKVGQKGKEGRSSRARQQAKGLTASWGPRNNPGKKELAKVSRTGRA--------------------------------------------------------------------------------------------------------------**

**3_Macaca_mulatta -------------------------------------------------------------------------------------------------------------------------------------------------------------------------------------------------------------------------------------------------------------------------------------------------------------------------------------------------------**

**3_Sus_scrofa -----------------------------------------------------------------------------------------------------------------------------------------------------------------WTEEMQDLIQQ-------------ETGKFDGWDK----------------------------------------------------------------------------------------------------------------------------------------------------**

**3_HUMAN -----------------------------------------------------------------------------------------------------------------------------------------------------------------WTEEMRDLVQR-------------ETGKLDGPDK----------------------------------------------------------------------------------------------------------------------------------------------------**

**4_Stigmatella_DW4/3 -------------------------------------------------------------------------------------------------------------------------------------------------------------------------------------------------------------------------------------------------------------------------------------------------------------------------------------------------------**

**4_Strongylocentrotus -------------------------------------------------------------------------------------------------------------------------------------------------------------------------------------------------------------------------------------------------------------------------------------------------------------------------------------------------------**

**4_Acyrthosiphon_pisum -------------------------------------------------------------------------------------------------------------------------------------------------------------------------------------------------------------------------------------------------------------------------------------------------------------------------------------------------------**

**4_Apis_mellifera -------------------------------------------------------------------------------------------------------------------------------------------------------------------------------------------------------------------------------------------------------------------------------------------------------------------------------------------------------**

**4_Drosophila -------------------------------------------------------------------------------------------------------------------------------------------------------------------------------------------------------------------------------------------------------------------------------------------------------------------------------------------------------**

**4_Tribolium_castaneum -------------------------------------------------------------------------------------------------------------------------------------------------------------------------------------------------------------------------------------------------------------------------------------------------------------------------------------------------------**

**4_Danio_rerio -------------------------------------------------------------------------------------------------------------------------------------------------------------------------------------------------------------------------------------------------------------------------------------------------------------------------------------------------------**

**4_Xenopus_tropicalis -------------------------------------------------------------------------------------------------------------------------------------------------------------------------------------------------------------------------------------------------------------------------------------------------------------------------------------------------------**

**4_Gallus_gallus -------------------------------------------------------------------------------------------------------------------------------------------------------------------------------------------------------------------------------------------------------------------------------------------------------------------------------------------------------**

**4_Taeniopygia_guttata -------------------------------------------------------------------------------------------------------------------------------------------------------------------------------------------------------------------------------------------------------------------------------------------------------------------------------------------------------**

**4_MOUSE -------------------------------------------------------------------------------------------------------------------------------------------------------------------------------------------------------------------------------------------------------------------------------------------------------------------------------------------------------**

**4_Bos_taurus -------------------------------------------------------------------------------------------------------------------------------------------------------------------------------------------------------------------------------------------------------------------------------------------------------------------------------------------------------**

**4_Equus_caballus -------------------------------------------------------------------------------------------------------------------------------------------------------------------------------------------------------------------------------------------------------------------------------------------------------------------------------------------------------**

**4_Pan_troglodytes -------------------------------------------------------------------------------------------------------------------------------------------------------------------------------------------------------------------------------------------------------------------------------------------------------------------------------------------------------**

**4_Canis_familiaris -------------------------------------------------------------------------------------------------------------------------------------------------------------------------------------------------------------------------------------------------------------------------------------------------------------------------------------------------------**

**4_Ovis_aries -------------------------------------------------------------------------------------------------------------------------------------------------------------------------------------------------------------------------------------------------------------------------------------------------------------------------------------------------------**

**4_Macaca_mulatta -------------------------------------------------------------------------------------------------------------------------------------------------------------------------------------------------------------------------------------------------------------------------------------------------------------------------------------------------------**

**4_Mus_musculus -------------------------------------------------------------------------------------------------------------------------------------------------------------------------------------------------------------------------------------------------------------------------------------------------------------------------------------------------------**

**4_Rattus_norvegicus -------------------------------------------------------------------------------------------------------------------------------------------------------------------------------------------------------------------------------------------------------------------------------------------------------------------------------------------------------**

**4_Sus_scrofa -------------------------------------------------------------------------------------------------------------------------------------------------------------------------------------------------------------------------------------------------------------------------------------------------------------------------------------------------------**

**4_HUMAN -------------------------------------------------------------------------------------------------------------------------------------------------------------------------------------------------------------------------------------------------------------------------------------------------------------------------------------------------------**

**5_Perkinsus_ATCC -------------------------------------------------------------------------------------------------------------------------------------------------------------------------------------------------------------------------------------------------------------------------------------------------------------------------------------------------------**

**5_Ciona_intestinalis -------------------------------------------------------------------------------------------------------------------------------------------------------------------------------------------------------------------------------------------------------------------------------------------------------------------------------------------------------**

**5_Apis_mellifera -------------------------------------------------------------------------------------------------------------------------------------------------------------------------------------------------------------------------------------------------------------------------------------------------------------------------------------------------------**

**5_Nasonia_vitripennis -------------------------------------------------------------------------------------------------------------------------------------------------------------------------------------------------------------------------------------------------------------------------------------------------------------------------------------------------------**

**5_Anoplopoma_fimbria -------------------------------------------------------------------------------------------------------------------------------------------------------------------------------------------------------------------------------------------------------------------------------------------------------------------------------------------------------**

**5_Danio_rerio -------------------------------------------------------------------------------------------------------------------------------------------------------------------------------------------------------------------------------------------------------------------------------------------------------------------------------------------------------**

**5_Salmo_salar -------------------------------------------------------------------------------------------------------------------------------------------------------------------------------------------------------------------------------------------------------------------------------------------------------------------------------------------------------**

**5_Taeniopygia_guttata -------------------------------------------------------------------------------------------------------------------------------------------------------------------------------------------------------------------------------------------------------------------------------------------------------------------------------------------------------**

**5_Bos_taurus -------------------------------------------------------------------------------------------------------------------------------------------------------------------------------------------------------------------------------------------------------------------------------------------------------------------------------------------------------**

**5_Canis_familiaris -------------------------------------------------------------------------------------------------------------------------------------------------------------------------------------------------------------------------------------------------------------------------------------------------------------------------------------------------------**

**5_Pan_troglodytes -------------------------------------------------------------------------------------------------------------------------------------------------------------------------------------------------------------------------------------------------------------------------------------------------------------------------------------------------------**

**5_Macaca_mulatta -------------------------------------------------------------------------------------------------------------------------------------------------------------------------------------------------------------------------------------------------------------------------------------------------------------------------------------------------------**

**5_Mus_musculus -------------------------------------------------------------------------------------------------------------------------------------------------------------------------------------------------------------------------------------------------------------------------------------------------------------------------------------------------------**

**5_Ornithorhynchus -------------------------------------------------------------------------------------------------------------------------------------------------------------------------------------------------------------------------------------------------------------------------------------------------------------------------------------------------------**

**5_Rattus_norvegicus -------------------------------------------------------------------------------------------------------------------------------------------------------------------------------------------------------------------------------------------------------------------------------------------------------------------------------------------------------**

**5_Sus_scrofa -------------------------------------------------------------------------------------------------------------------------------------------------------------------------------------------------------------------------------------------------------------------------------------------------------------------------------------------------------**

**5_HUMAN -------------------------------------------------------------------------------------------------------------------------------------------------------------------------------------------------------------------------------------------------------------------------------------------------------------------------------------------------------**

**6_Strongylocentrotus SGAVKRDAEGNIKVC----------------------------------------------------------------------------------------------------------------------------------------------------------------------------------------------------------------------------------------------------------------------------------------------------------------------------------------**

**6_Lepeophtheirus -------------------------------------------------------------------------------------------------------------------------------------------------------------------------------------------------------------------------------------------------------------------------------------------------------------------------------------------------------**

**6_Acyrthosiphon_pisum -------------------------------------------------------------------------------------------------------------------------------------------------------------------------------------------------------------------------------------------------------------------------------------------------------------------------------------------------------**

**6_Apis_mellifera -------------------------------------------------------------------------------------------------------------------------------------------------------------------------------------------------------------------------------------------------------------------------------------------------------------------------------------------------------**

**6_Drosophila -------------------------------------------------------------------------------------------------------------------------------------------------------------------------------------------------------------------------------------------------------------------------------------------------------------------------------------------------------**

**6_Danio_rerio -------------------------------------------------------------------------------------------------------------------------------------------------------------------------------------------------------------------------------------------------------------------------------------------------------------------------------------------------------**

**6_Xenopus_tropicalis -------------------------------------------------------------------------------------------------------------------------------------------------------------------------------------------------------------------------------------------------------------------------------------------------------------------------------------------------------**

**6_Gallus_gallus -------------------------------------------------------------------------------------------------------------------------------------------------------------------------------------------------------------------------------------------------------------------------------------------------------------------------------------------------------**

**6_Bos_taurus -------------------------------------------------------------------------------------------------------------------------------------------------------------------------------------------------------------------------------------------------------------------------------------------------------------------------------------------------------**

**6_Canis_familiaris -------------------------------------------------------------------------------------------------------------------------------------------------------------------------------------------------------------------------------------------------------------------------------------------------------------------------------------------------------**

**6_Mus_musculus -------------------------------------------------------------------------------------------------------------------------------------------------------------------------------------------------------------------------------------------------------------------------------------------------------------------------------------------------------**

**6_Pan_troglodytes -------------------------------------------------------------------------------------------------------------------------------------------------------------------------------------------------------------------------------------------------------------------------------------------------------------------------------------------------------**

**6_Sus_scrofa -------------------------------------------------------------------------------------------------------------------------------------------------------------------------------------------------------------------------------------------------------------------------------------------------------------------------------------------------------**

**6_Macaca_mulatta -------------------------------------------------------------------------------------------------------------------------------------------------------------------------------------------------------------------------------------------------------------------------------------------------------------------------------------------------------**

**6_Rattus_norvegicus -------------------------------------------------------------------------------------------------------------------------------------------------------------------------------------------------------------------------------------------------------------------------------------------------------------------------------------------------------**

**6_HUMAN -------------------------------------------------------------------------------------------------------------------------------------------------------------------------------------------------------------------------------------------------------------------------------------------------------------------------------------------------------**

**7_Ciona_intestinalis -------------------------------------------------------------------------------------------------------------------------------------------------------------------------------------------------------------------------------------------------------------------------------------------------------------------------------------------------------**

**7_Apis_mellifera -------------------------------------------------------------------------------------------------------------------------------------------------------------------------------------------------------------------------------------------------------------------------------------------------------------------------------------------------------**

**7_Danio_rerio -------------------------------------------------------------------------------------------------------------------------------------------------------------------------------------------------------------------------------------------------------------------------------------------------------------------------------------------------------**

**7_Xenopus_tropicalis -------------------------------------------------------------------------------------------------------------------------------------------------------------------------------------------------------------------------------------------------------------------------------------------------------------------------------------------------------**

**7_Bos_taurus -------------------------------------------------------------------------------------------------------------------------------------------------------------------------------------------------------------------------------------------------------------------------------------------------------------------------------------------------------**

**7_Canis_familiaris -------------------------------------------------------------------------------------------------------------------------------------------------------------------------------------------------------------------------------------------------------------------------------------------------------------------------------------------------------**

**7_Equus_caballus -------------------------------------------------------------------------------------------------------------------------------------------------------------------------------------------------------------------------------------------------------------------------------------------------------------------------------------------------------**

**7_Macaca_mulatta -------------------------------------------------------------------------------------------------------------------------------------------------------------------------------------------------------------------------------------------------------------------------------------------------------------------------------------------------------**

**7_Mus_musculus -------------------------------------------------------------------------------------------------------------------------------------------------------------------------------------------------------------------------------------------------------------------------------------------------------------------------------------------------------**

**7_Pan_troglodytes -------------------------------------------------------------------------------------------------------------------------------------------------------------------------------------------------------------------------------------------------------------------------------------------------------------------------------------------------------**

**7_aRattus_norvegicus -------------------------------------------------------------------------------------------------------------------------------------------------------------------------------------------------------------------------------------------------------------------------------------------------------------------------------------------------------**

**7_Sus_scrofa -------------------------------------------------------------------------------------------------------------------------------------------------------------------------------------------------------------------------------------------------------------------------------------------------------------------------------------------------------**

**7_HUMAN -------------------------------------------------------------------------------------------------------------------------------------------------------------------------------------------------------------------------------------------------------------------------------------------------------------------------------------------------------**

**C) SIRTUINS – AMINO TERMINAL SEGMENTS: central core (missing) on the right side**

**A)NEGATIVE SEQUENCE STRETCHS IN RED,**

**B)POSITIVE SEQUENCE STRETCHS IN LIGHT BLUE,**

**C)PREDICTED PHOSPHORYLATION SITE IN GREEN**

**1-HyDRa_mag --------------------------------------------------------------------------------------------------------------------------------------------------------------MADPIANNSMQLVKD----------EATFSPHDFIKN--PLQWIQNQIENEVD-PR-ILIRQLVPQIK---LPHDVEDSTLWNVIFEIIS------------------------EPSPRKRLSNINSL**

**1.Stron_p ---------------------------------------------------------------------------------------------------------EEGHVEQEVSDGPAPSWGSQASSSDSSSSSSSGHQPGGDSSSEGSITDSFDMGLGDIRPDSLAGVLHC----------NHSYLVCDTLPQPGPMGWLQKQMMSGTN-PKSILMRIIPSGMT---IPEEMDEFEMWSIIAEYLRSI---------------------DEPPPRQKLEQYNTF**

**1.Brugia_mal ----------------------------------------------------------------------------------------------------------------------------------------MHSGGCGNELISENIVLDGNMGTETDTYASSVTNSSSMG--NSAVQMQGLTDSRDSSADSGDISAVDEVSGESES-ARDVVRCLLPHVN----LPENLCEQDLYRIIQKILYS-----------------------ERPKRTKLSEFNSF**

**11aSchis_ma ---------------------------------------------------------------------------------------------------------------MDTKKEPSISIDNSEIVVLSSDDENDSQNSHDNISNEKLSSSVISIEDDDDNNTNNDESAES---DCEILNVEDLQGEEKWRDIHGPFKRLNRLIQAGFNDPRLLLVRVFGMDE----NSLPSDPNQLLSLLLTLLA------------------------EPAPRRRLRRINSL**

**1Schis_ja ---------------------------------------------------------------------------------------------------------------MDTNKEQSVDVDNSQIVVLSSDDENDSQNSHDNLSNEKGSSSVISVDDDDDN-TNNDESAES---DCEILSVEDLQGEEKWRDIHGPFKRLSSLIQAGFNDPRLLLVRVFGMDE----DSLPSDPNQLLSLLLTLLA------------------------EPAPRRRLRHINSL**

**1Acyrt_pis MASEDNGDFDTDLSAAPAKRMRYSFADEIARTSHFEAFGQRFTGFPSGECYANNLYQQS----------------------------------------------------YKSLDTLS-PNYLQPNSPTPPLTMSSSLPESPEDS----SIFDESSDTKEEPEDDDSSSTSSS---DSSTHSENADSE---KGVPGSMDWVQRQIMGGIN-PRRLLHQVFGAS-----VPSQLEDITLWRIIMSMTD------------------------DSPIRNRLRSVSSL**

**1Trib_cas MDT--YPDVAEHESSAKRIKLDLR-ESNIGELCNGGDFSSYSTSVGGPIDIPEGISPDC-------------------------------------------------DSGYEASTLESIPTSSHQTSPHTDPGDVPESLSSLSEIFLTPSRTDQADTSQDSIEADDDNASTVS---EISGLSDLSGQD--WKPMAGSMIWIQKQMQNGVN-PRTLLSDLGVDL---DQVPQYVDEITLWKLIINMLA------------------------EPPRRNKLRHVNTL**

**1Apis_mel MAS--GSELPEYSSPAKRRKVDGIGYSGTSSQ--KPDF----QDCETSHDIPNEDPEET-------------------------------------------------YGGDSGFNELSDESKSTSISPDA-----TNLMTTPSRI---DSTSDDTGCLIDTADEKDEVSSTVSNLSDLSGLSDFSGEGDINHQWRNASSWVQKQMLIGAD-PRNLLHHLLMDS---TQIPEQVDDLTLWKIIINMMS------------------------EPPRRQKLRHINTL**

**1Pedi_Hu_c MAS--NCDVQERTSSVKKQKIECS-SSGTLENNIKMDVNLFSDKFSTSSEILKESVQENRIAPLSTEQ--------------------------------------VLNGGDSGFSEIEMTSSTEVLSENARLQSFENDIQSPVPG---SSTFDSSMEIRDELNDNDDISSTIS---DLSGISDLSGQD--WKPTSGPMSWIHHQMANGAD-PRDLLFQLVQDKAAIAALPSGKDDFTLWKIIISMLS------------------------EPPRRKKLSTINTL**

**1Danio_Rer ----------------------------------------------------------------------------------------------------------------------------------------------------------------------------------------------------------------------------------------------------------------------------------------------**

**1aNoth_fu -MADEENSLGTAFSGAFITDEPATKISKMDTPTNHGLKPTEPDQLSRVFAATESREAAIKRSQPSEKE-----------------------------------------AKPVMEVEQAPTAARDGDNNERGIPVSEPQRPAGKLVDSAVLCSTEEVAGHDDLPSNGLAASPENLNDEDDRSSHASSSDWTPQPQIGSYSFIQQHIR-ETD-PRAILRDLLPETI----LPPDLDDMTLWQIIIN-IS------------------------EPPKRKKRKDINTL**

**1bNoth_KuH -MADEENSLGTAFSGAFITDEPATKISKMDTPTNHGLKPTDPDQLSRVFAATESREAAIKRSQPSERE-----------------------------------------AKPVMEVEQAPTAARDGDNNERGIPVSEPQRPAGKLVDSAVLCSTEEVAGHDDLPSNGLAASPENLNDEDDRSSHASSSDWTPQPQIGSYSFIQQHIR-ETD-PRAILRDLLPETI----LPPDLDDMTLWQIIIN-IS------------------------EPPKRKKRKDINTL**

**1Xenop --DSDRVGFPIAGAVLAVSKE-------NGEPLSKRQRLEDTGGGGSQLVGAESEGKAALPPIAASLQ-------------------------------EEGEASSAMESKSRAHNGSGFQGLPLGTYLVQGQEEGGAEEMPNGDLSDQAVDYGAGIHLDDDLAG-GFHSCDS---EDDDGASHASSSDWAPRPCIGPYTFVQRHLMMGTD-PRTILKDLLPDTV----APSELDDMTLWQIVINILS------------------------DPPKRKKRKDINTI**

**1Taeniop MPNTLENKISPR-------------------------------------------------CTVPLG----------------------------------------------------------------WSVIPCS------------CCLADNFLLSDEIIANGFHSCDS---DEEDRASHASSSDWTPRPRIGPYTFVQQHLMLGTD-PRTILKDLLPETI----PPPELDDMTLWQIVINILS------------------------EPPKRKKRKDINTI**

**1Gallus MADGEAPLLRPRDGGPGAAAESVEPAPKRQRLNSEDGVCGRGAPPAHRPDRGAGPPPAAAAATEPPGDAAAVSADGDVRAREEDGGATTEGRSGADNRAAQRGLARAEPPPQPRRQGRGEGAEAAPGEDAAEAAIGCERAQRSNGAAGAPAPQPDNFLLSDEIIANGFHSCDS---DEDDRASHASSSDWTPRPRIGPYTFVQQHLMLGTD-PRTILKDLLPETI----PPPELDDMTLWQIVINILS------------------------EPPKRKKRKDVNTI**

**1Equus_cab --------MVSGKSESFVGVGKHLT----AQRIYVHP----------------------------------------------------------------------------------VKRET------------------------------DDLLFGDEIITNGFHSCES---DEDDRASHASSSDWTPRPRIGPYTFVQQHLMIGTD-PRTILKDLLPETI----PPPELDDMTLWQIVINILS------------------------EPPKRKKRKDINTI**

**1Mac_mul MADEAALALQPGGSPSAAGAEREAGSPPAGEPLRKRPRRDGPGLERSPGEPGGAAPEREVPAAG-GCP-VAAAALWR------------EAEAAAAGGEQEAQATAAAGEGDNGPGLQGPSREPPLADSFYDEDDDDEGEEEEE-AAAAAIGYRDNLLFGDEIITNGFHSCES---DEEDRASHASSSDWTPRPRIGPYTFVQQHLMIGTD-PRTILKDLLPETI----PPPELDDMTLWQIVINILS------------------------EPPKRKKRKDINTI**

**1Monode MADQAALALEP-------SAGGAGPAEPGGEPLSKRQRRDGPGQGPGAERAVGAGSPGPEAAAAPLGEASEAAAPGG-----------------------DNGPGPRSQRGLPREPPPAPPDDDDDDEEEDDDEEEEEEGGEDEDEAAAAIGYRENLFS-DETLANGFHSCDS---DEDDRASHASSSDWTPRPRTGPYTFVQQHLMIGTD-PRTILKDLLPETI----PPPELDDMTLWQIVINILS------------------------EPPKRKKRKDINTI**

**1Ornitho -----------------------------------------------------------------------------------------------------------------------------------------------------------------MTRVGGCQSSG------------------------------------GTQ-SR------------------------------------------------------------------------**

**1Canis_fam MADEAALALQPGGSPSAVAAEREAASPPAGEPLRKRPRRDCPGLGRSPGEPGGAAPEREVPAATGGCS-AAAAALWR--------------EAAAGGEREAQAAVAAAGEGDNGPGLQGLTREPLPADDFVDDDDDDEGEEEEEAAAASAIGYRDNLLFDDEIITNGFHSCES---DEDDRASHASSSDWTPRPRIGPYTFVQQHLMIGTD-PRTILKDLLPETI----PPPELDDMTLWQIVINILS------------------------EPPKRKKRKDINTI**

**1Bos_ta MADEAALALQPGGSPSVVAAEREAPSPPAGEPLRKRPRRDGPGVGRSSGEPGGTAPERELPAAAGSCP-AAAAALWR--------------EAQA-------AAAAAAEEEDNGPGLQGLSREAPPADDFYDDD--DEGEEEEE--AAAAIGYRDNLLFGDEIITNGFHSCES---DEDDRASHASSSDWTPRPRIGPYTFVQQHLMIGTD-PRTILKDLLPETI----PPPELDDMTLWQIVINILS------------------------EPPKRKKRKDINTI**

**1Sus_sc MADEAALALQPGGSPSAVAAEREAPSPPAGEPLRKKPRRDGPGVGRSPGEPGGAALERELPAAAGGCP--AAAVLWR--------------ETAAGGEREAQAA-AAAGEGNNGPGLQGLSREAPPADDFYDDDDDDEGEEEEEAAAAAAIGYRDNLLFGDEIVTNGFHSCES---DEDDRASHASSSDWTPRPRIGPYTFVQQHLMIGTD-PRTILKDLLPETI----PPPELDDMTLWQIVINILS------------------------EPPKRKKRKDINTI**

**1Rattus_no --------------------------------------------------------------------------------------------------------------------------------------------------------------------------------------------------------------MIGTD-PRTILKDLLPETI----PPPELDDMTLWQIVINILS------------------------EPPKRKKRKDINTI**

**1Mus_musc MADEVALALQAAGSPSAAAAM-EAASQPADEPLRKRPRRDGPGLGRSPGEPSAAVAPAAAGCEAASAA--APAALWR---------------EAAGAAASAEREAPATAVAGDGDNGSGLRREPRAADDFDDDEGEEEDEAAAA-AAAAAIGYRDNLLLTDGLLTNGFHSCES---DDDDRTSHASSSDWTPRPRIGPYTFVQQHLMIGTD-PRTILKDLLPETI----PPPELDDMTLWQIVINILS------------------------EPPKRKKRKDINTI**

**1HUMAN MADEAALALQPGGSPSAAGADREAASSPAGEPLRKRPRRDGPGLERSPGEPGGAAPEREVPAAARGCPGAAAAALWRE----------AEAEAAAAGGEQEAQATAAAGEGDNGPGLQGPSREPPLADNLYDEDDDDEGEEEEE-AAAAAIGYRDNLLFGDEIITNGFHSCES---DEEDRASHASSSDWTPRPRIGPYTFVQQHLMIGTD-PRTILKDLLPETI----PPPELDDMTLWQIVINILS------------------------EPPKRKKRKDINTI**

**14 27 47 159 162 172 173 174**

**2_Aj_DERSLH1 -----------------------------------------------------------------------------------------------------------------------------------------------------------------------------------------------------------------------------------------MGNENSVLVDGSTAP----------------------------VTLKSRSVEG**

**2_aMic_CCMP ----------------------------------------------------------------------------------------------------------------------------------MGGSDPLPGDDPRDEAPAGITDDARRDDTTAKTDSTVEASDGSEEEHSNRGLAEVDSTDAVASV------------SGSDDDEEVEEELTGLAALIRKLALKAEEKNATEAAAEEEP-----------------------ATFDTTPLLSTFDVAG**

**2_OstR_tau ----------------------------------------------------------------------------------------------------------------------------------MARDDAQS-DVSVDGANARDAEDARSSDDDAVNDASSSSSSSSSSSSSGYGFGR---------------------------------ESAEMVALMERLAAQRLG-TTTEALAR--------------------------ATVDKA—LESFDLAGV**

**2_TRic_aD -------------------------------------------------------------------------------------------------------------------------------------------------------------------------------------------------------------------------------LENVRSLLQRFNIFVGEN-DSEAEQP--------------------------QQLLSEVTFEG**

**2_NEma_vEct -----------------------------------------------------------------------------------------------------------------------------------------------------------------------------------------------------------------------------------MKLFNQFRLNLTQE-KSEKEKP--------------------------EQLLDEVSFEG**

**2_BRanc_fl --------------------------------------------------------------------------------------------------------------------------------------------------------------------------------------------------------------------------------------VEHLRRLFARTLGLSQERET------------------EEGTARPQQVLDEVTVEG**

**2_StRong_pu ------------------------------------------------------------------------------------------------------------------------------------------MSAKNEGAAAG-----------------------------------------ADDKEGGQSESVDSQ------------------VESLRNFLGRFHLSAGSSGQEEKPKP--------------------------EQLLKELTLEG**

**2_Cio_intE -----------------------------------------------------------------------------------------------------------------------------------------------------------------MAEGSNKSDDLP-------------------------KEAE---------------------SSLSANEVDQLKNLFS-TINLG-ELIN------------------KDNEEKPEQLLPEVTFKG**

**2_BRu_mal ------------------------------------------------------------------------------------------------------------------------------------------MSEEELTGN---------SSKKNKHRSSPLMESSKP----------------------------------------------TDNKNSISPSALEMLMTKLEKLTTKEKEKD------------------------TKQKLSSLTIEG**

**2_aScHis_j -------------------------------------------------------------------------------------------------------------------------------------------------------------------------------------------------------------------------------------MSFNFDRLKKTLFGGDKCP----------------------------PKLKSFDIEG**

**2_ScH_m -------------------------------------------------------------------------------------------------------------------------------------------------------------------------------------------------------------------------------------MSFDFLGIKKALFGDNTPR----------------------------PELKSLNIEG**

**2_aCali_clE ------------------------------------------------------------------------------------------------------------------------------------------MSGSDVDKH---------EHLRNPEYFSGSSSDDED----------------------------------------------DEDLRLFGNSDEIDGYLANRILGLDMNQPL------------------------PEQLLDEVSFSG**

**2_Cali_Rog ------------------------------------------------------------------------------------------------------------------------------------------MSGEEMDKE---------DRLRDPEYFSGSSSSDDDD--------------------------------------------IEEHLRLLGHSDDIDGYLASRILHLDMNQPR------------------------PAQLLDEVSFEG**

**2_LEpEo_sal ------------------------------------------------------------------------------------------------------------------------------------------MSGGDGALL---------DKLDDPEYCSGSSSEDER-----------------------------------------------NNIDLFGNNDEIDGYLANRFLDLGVNHYY------------------------PEQILDEVSFSG**

**2_AEDE_aE ------------------------------------------------------------------------------------------------------------------------------------------MSAENTLPDLLGGGAQGAKPDNKPLVDGEEAGGSVTSNSVPKASPLELDTAGSGRESNNSSQVQQAPDFSQFDDSEDEEHYHSGGFHSDNISIERIRQYLSDKLGFYTTDSNY----------------DDKDGVPRKRVLETVDIDG**

**2_Apis_mEl ---------------------------------------------------------------------------------------------------------------------------------------------MKLYHNGREKKSN----EETTNISSEQEEIEESD-------------------------------------------------------MEKIRKYLAQKLRLFDSS--------------------NNNEQNNPNILRELSIDG**

**2_Bomb_mo ------------------------------------------------------------------------------------------------------------------------------------------MSANSPPGKSGGHETVDESPQNVPPTTSMESLRNMFR----------------------------------------------------DLDVDDVRMYLALKLGLFSPQ-------------------DLEPAEPPEKVLDEVSLMG**

**2_DRoso_mE --------------------------------------------------------------------------------------------------------------------------------------------------------------------------------------------------------------------------------------MDKVRRFFANTLHLGGSS-------------------DAKEEVKVEKVIPDLSFDG**

**2_PEDi_Hu_c --------------------------------------------------------------------------------------------------------------------------------------------------------------------------------------------------------------------------------------MGANCSLLPVACSFCGQS----------------------KKEKAVQVLDEVSITG**

**2_TRib_ca ------------------------------------------------------------------------------------------------------------------------------------------MSRDSSK------DRAEAKPKQPPEAQSEEDNASGTS-----------------------------------------------------MSIDSLRKYLAEKLGISDKD-------------------DEKE---KIKILDDVSVDG**

**2_Danio_RER -------------------------------------------------------------------------------------------------------------------------------------------------------------MS--EEVSKRVEEEAD---TP-----------GLEGQSDDSSDEG---------------------DASGDTEMDFLRSLFSRTLGLSPGD----------------------------KVLDELTLDS**

**2_Salmo_sal -------------------------------------------------------------------------------------------------------------------------------------------------------------MSDVPEASNREEVEDN---TP-----------EPEEQSDDSSDDG---------------------EASGDTEMDFLRNLFSKTLGIS-GE----------------------------KVLDELTLEG**

**2_XE_laE -----------------------------------------------------------------------------------------------------------------------------------MERNPVR---------------KAIPERQLQETCDRSETSDEGKRSPSSHKTESSEPADLKAQAEDSEDSDSSE------------------DNSGASEMDFLRNLFSRTLGIGTPE----------------------------KVLDELSLES**

**2_Gallus ------------------------------------------------------------------------------------------------------------------------------------------MSEADG------------NGGGGVEPGGSLRRSVTPRPIPAAPP--------PGADPDAEAEGG---------------------SEE-DSDMELLRNLLARTLGLGTEPP--------------------------ERVLDELSLAG**

**2_Bostau ------------------------------------------------------------------------------------------------------------------------------------------MADPDP------------SDP--EETQAGKVQEAQ------------------DSDSD--TEAG---------------------ATGGEAEMDFLRNFFSQTLGLGTQK---------------------------ERLLDELTLEG**

**2_Canis_fam --------------------------------------------------------------------------------------------------------------------------------------------------------------------------------------------------------------------------------MAACAEPSGTRSLAVRVF-LARER---------------------------QAPLDEK----**

**2_Equusca ------------------------------------------------------------------------------------------------------------------------------------------MAEPDP------------SDP--LETQAGKVQEAQ------------------DSDSD--TEGG---------------------AAGGEAEMDFLRNFFSQTLGLGTQK---------------------------ERLLDDLTLEG**

**2_RattusnoRv -----------------------------------------------------------------------------------------------------------------------------------------MKAVTGPRCLSGVVFFYPLSAPSCFRCRHGTEQSVTVPRAPTPFPW----PSRTDSDSD--TEGG---------------------ATGGEAEMDFLRNLFTQTLGLGSQK---------------------------ERLLDELTLEG**

**2_Musmuscu ------------------------------------------------------------------------------------------------------------------------------------------MAEPDP------------SDP--LETQAGKVQEAQ------------------DSDSD--TEGG---------------------ATGGEAEMDFLRNLFTQTLGLGSQK---------------------------ERLLDELTLEG**

**2_SusscR ------------------------------------------------------------------------------------------------------------------------------------------MAEPDP------------SDP--VETQTGKVQEAQ------------------DSDSDSDTEEG---------------------AAGGEAEMDFLRNFFSQTLGLGTQK---------------------------ERLLDELTLEG**

**2_HUMAN ------------------------------------------------------------------------------------------------------------------------------------------MAEPDP------------SHP--LETQAGKVQEAQ------------------DSDSD--SEGG---------------------AAGGEADMDFLRNLFSQTLSLGSQK---------------------------ERLLDELTLEG**

**3_Ciona_intE ----------------------------------------------------------------------------------------------------------------------------------MKCLGRCWLG-GKVGKLFIIQIKQECQKSLFLLKMNKSYDEGTGQRNGATTKKMENDQSFVEGDFSKSCSNKLKSGVEKTKKKTVKKKKKKKQEQKDEVTKISKSFSKLDINRRDVSKQRT----------------PQRRSQLGKTPSSLSSIKD**

**3_XEno_tRo ----------------------------------------------------------------------------------------------------------------------------------MSFHTSHFSGQDPVGKICSTPLSPKAATCVDQWPDAKMEVSATNS---AFQTTVINNQ--------------------NAQEAADLKHPTEYHSRKRENGRLSGVIRQSCLRKRQLPKSAL----------------AKNISAIKPN-IGCNNLED**

**3_Danio_RE --------------------------------------------------------------------------------------------------------------------------------------------------MSKARLS----------RDRRAASVGVSR---VTRSSMMSPQ--------------------DCERSRAPDP-----GLLDELSLMSVSEQQASATRKGSSKPAL----------------S---SPSGRS-VSRGALET**

**3_Gallus ----------------------------------------------------------------------------------------------------------------------------------MERGVRRGAALVAAWRSLWER--------------------------------------------------------GGLALFRPQCRTGCGACRVQGTRPFSLSAAASAVLGLGS--------------------------WGGDSGKQKLTLQD**

**3_TaEniopy ---------------------------------------------------------------------------------------------------------------------------------------------MAVPSPAGHR--------------------------------------------------------AVIPLDKDTQSP-----RIQGSRPFCLSTAARAILGW----------------------------WGGDEGKQKLTLKD**

**3_Equ_cab ------------------------------------------------------------------------------------------------------------------------------------------------------------------------------------------------------------------------MVVLDS-GITGGRRPISFSAGASSVFGS-----------------------------GGNSRKEKLLLQD**

**3_isCan_fa ----------------------------------------------------------------------------------------------------------------------------------MTRCARPALAALGLWGPAGWRSLYTGVRDVLGEGHQRPSPGRMDMGAASLWG-------------------------VRKAPCAVELAVLNS-GITGGRRPISFSTRTSSIFGS-----------------------------GGD-HKKKLFLQD**

**3_Mus_mus -------------------------------------------------------------------------------------------------------------------------------------------------------------------------MALDPLGAVVLQS-------------------------IMALSGRLALAALRLWGPGGGRRPISLCVGASGGFGG-----------------------------GGS-SEKKFSLQD**

**3_aRat_noRv ----------------------------------------------------------------------------------------------------------------------------------------------------------------------------------------------------------------MALSCRLVLAALRLWGPGGGRRPISLSVGASGGFGC-----------------------------GGH-SEKKLSLQD**

**3_ORy_cun ----------------------------------------------------------------------------------------------------------------------------------MALWSQRVVAALRLWG-----------------------------------------------------------------------P-------IGGRRPVSLSARAPSISGS-----------------------------RGRPER—FSLQDV**

**3_MonoD_Dom -----------------------MGQTSRRKRTLTSGAARTYFRRARTRYFRLRPRVRQAGVPSGHSGSWSTPVLRPGPPWVLRWGPREGSPRPPAVSVPPCACVGVRAAAGAEEGPLSRFPTAPLPSQSTIPRASQPVGKWNQWGVLRDRRLTLLGRACGSPLPPPVASLGALHRPLPSLCQGGGVSACAVREAPLEDLPSLEFGGAFEPSTDPAASLALCGGRCLHRGLWEGKLGPGLAFGC-----------------------------EVSTSSKKLSLQD**

**3_Mac_mu ----------------------------------------------------------------------------------------------------------------------------------MAFWGWRAVAAVRVWGRVAERAEAGGDVGPFQAWSCRLVLGGRDDVSAGPRGSRGVCGEPLDSARPLQRPPRPAVPRAFRRQPRAAAPGFFFSSIKGGRRPISFSVGASSVVGS-----------------------------GGDSDKGKLSLQD**

**3_Sus_scR ----------------------------------------------------------------------------------------------------------------------------------------------------------------------------------------------------------------------------------------------------------------------------------------------**

**3_HUMAN ----------------------------------------------------------------------------------------------------------------------------------MAFWGWRAAAALRLWGRVVERVEAGGGVGPFQACGCRLVLGGRDDVSAGLRGSHGARGEPLDPARPLQRPPRPEVPRAFRRQPRAAAPSFFFSSIKGGRRSISFSVGASSVVGS-----------------------------GGSSDKGKLSLQD**

**SIRT4ARAB ------------------------------------------------------------------------------------------------------------------------------------MKLHQDKNRRESHERIFDRELTRSSSRERDRSEGEIEAKERELRPQRENKIDTSDFCHLVFNNNKKFWKKIKRKTEEISFHPPLT----------TSQDLF-PSR-SMYRPLQSGGNLVMLFKGCRRFVRTTCRVSIPGGSLGNESKAPPRFLR**

**SIRT4ORIZA --------------------------------------------------------------------------------------------------------------------------------------------------MAAGAHASRASAPIIAGLTGALRAAYKGFSPQLCN-------FHASVNNG-----LLHRRKIQLHFICSFR----------SIQARY-NHS-SAVAPKDY----------CETYIQ---------------------FLR**

**SIRT4PHYSCOM ------------------------------------------------------------------------------------------------------------------------------------------------------MWKPNHSIRCLQLQTG----ARNKLSPCLLN-------------------------STRFCNYATLA----------ATGSVA-VPQ-EISSSLNS----------------------------------------**

**4_Stig_auRa -----------------------------------------------------------------------------------------------------------------------------------------------------------------------------------------------------------------------------------------------------------------------------MSALPVPPSPTSVPSNV**

**4_StRon_pu ----------------------------------------------------------------------------------------------------------------------------------------------------------------------------------------------------------------------------------------------MGEREGG------------------------FLSQFVPESRPVTESSI**

**4_Acy_pisum --------------------------------------------------------------------------------------------------------------------------------------------------------------------------------------------------------------------------------------------------------------------------------------MPVQSSDA**

**4_Apis_ --------------------------------------------------------------------------------------------------------------------------------------------------------------------------------------------------------------------MRSYKS-----------------ANIIETLLYF---------------------------LFVPKCEPTKDSYL**

**4_DRoso_mE ---------------------------------------------------------------------------------------------------------------------------------------------------------------------------------------------------------------MRVGQLLRFRS-----------------TSLRSSTARQ---------------------------EYVPHHKPVVEDDI**

**4_TRibol -----------------------------------------------------------------------------------------------------------------------------------------------------------------------------------------------------------------MSNLTRQLP-----------------FLLKRTPILS---------------------------DFIPRHSPAPSTDV**

**4_Danio_RE -----------------------------------------------------------------------------------------------------------------------------------------------------------------------------------------------------------------MLLSCRYLP------------PPVAVGRCASTIQAG-------------------------VRQFVPASGSFDSSAL**

**4_XEno_tRo -----------------------------------------------------------------------------------------------------------------------------------------------------------------------------------------------------------------MWKNVREG-----------SKVFWGINNITRSHKSH-----------------------LALSEFVPACPPPNPHQV**

**4_Gallus ---------------------------------------MAGYWEKFSTSNNRASDKPHWLRYCHCAKLTAPSAPPTRPEAAPPPTSQRAGALPRAPHLRPRAPPAAAAPRSERRCVRGGARRSGAAAELERGVCREPAMVMSAGRAAPAREEGLRRAAEGGCEALGAVPGLYPCRKLGFCSLNHGNCSRCAELCTDCISHKALLAGSRSRSWQRYERAFRVCAKANAVCGPR-SLNMFPARRLPGGCRAIRPHHLRH----HSVPSASPNLAFVPACLPPHPAEV**

**4_TaE_gut ---------------------------------------MQHTWEKQ--REDEESSKRGWA--------------PLIPAALPTP-------YPRKVQISPKN-------RLER---------------LRRG--REEMFAETRVRST------LLRAQE---------------RRAPFP-------AGTATACPGCGWTAVPPACPASSAPRRYR--------GAPFCPPF-WAPVTPAKAAG-----------------REYAKASPNLTFVPACLPPDPAEV**

**4_MOUSE -----------------------------------------------------------------------------------------------------------------------------------------------------------------------------------------------------------------MS-GLTFR----------PTKGRW-ITHLSRPRSCG------------------------PSGLFVPPSPPLDPEKI**

**4_Bos_tau ---------------------------------------------------------------------------------------------------------------------------------------------------------------------------------------------------------------MRMSFGLTFKR---------TAKVHW-RANFSQQCSLR------------------------STGLFVPPSPPLDPEKV**

**4_Equus_cab ---------------------------------------------------------------------------------------------------------------------------------------------------------------------------------------------------------------MRMSFGLTFR----------TAKGHW-LVNLSRQCSHG------------------------STGLFVPPSPPLDPEKV**

**4_Pan_tRog ---------------------------------------------------------------------------------------------------------------------------------------------------------------------------------------------------------------MKMSFALTFR----------SAKGRW-IANPSQPCSKA------------------------SIGLFVPASPPLDPEKV**

**4_Cani_fam -----------------------------------------------------------------------------------------------------------------------------------------------------------------------------------------------------------------MNLGLTLK----------APKGLL-MVNISRQYSRR------------------------SIGFFVPSSPPLDPEKV**

**4_Ovis_aRi ---------------------------------------------------------------------------------------------------------------------------------------------------------------------------------------------------------------MRMSFGLTFKR---------TAKVRW-RANFSQQCSLR------------------------STGLFVPPSPPLDTEKV**

**4_Macaca_mu ---------------------------------------------------------------------------------------------------------------------------------------------------------------------------------------------------------------MKMSFGLTFK----------SAKGRW-IANPSWQCSKA------------------------SIGLFVPASPPLDPEKV**

**4_Mus_mus ----------------------------------------------------------------------------------------------------------------------------MAWVEAQPQREKEGVSSISGTLTELSRKDSGQSVEERPRILAWETRRILGLVVFFQLDRLRGLHFPRCTRNKRVPLLGENCGRIRMS-GLTFR----------PTKGRW-ITHLSRPRSCG------------------------PSGLFVPPSPPLDPEKI**

**4_Ratt_noR -----------------------------------------------------------------------------------------------------------------------------------------------------------------------------------------------------------------MR-GLIFR----------PTRGRW-ITQMSQLRSHG------------------------STGLFVPPSPPLDHEKI**

**4_Sus_scR ---------------------------------------------------------------------------------------------------------------------------------------------------------------------------------------------------------------MRMSFGLIFR----------TAKGRW-MAKLSRQCSRG------------------------YTELFVPSSPPLDPEKA**

**4_HUMAN ---------------------------------------------------------------------------------------------------------------------------------------------------------------------------------------------------------------MKMSFALTFR----------SAKGRW-IANPSQPCSKA------------------------SIGLFVPASPPLDPEKV**

**SIRT5PHYSCOM -----------------------------------------------------------------------------------------------------------------------------------------------------------------------------------------------------------------------------------MPRAGVAASAFKLRNIWH-------------------IHNLHRVRSQQTRLMS---DSV**

**SIRT5ORIZA ----------------------------------------------------------------------------------------MYYGFDIGGTKIALGVFDKDLRLQWETRVPTPRESYDEFLTAIAALVAQADERFGVKGSVGIGIPGMPETDDGTLYAANVPAASGKALRADLSARLERDVRLDNDANCFALSEAWDDEFRQYPLVMGLILGTGVGGGIVINGKPITGRSYITGEFGHIRLPVDALDIVGREFPLTRCGCGQHGCIENYLSGRGFAWLY**

**5_PERKin_m --------------------------------------------------------------------------------------------------------------------------------------------------------------------------------------------------------------------------------------------------------------------------------------MMMSIRGL**

**5_Ciona_int --------------------------------------------------------------------------------------------------------------------------------------------------------------------------------------------------------------------------------------------------------------------------------------MSRPSSDM**

**5_Apis_mEl ----------------------------------------------------------------------------------------------------------------------------------------------------------------------------------------------------------------------------------------------------------------------------MN--------------TM**

**5_Nason_vit ----------------------------------------------------------------------------------------------------------------------------------------------------------------------------------------------------------------------------------------------------------------------------MSSLPRTDTCITERYATY**

**5_Anopl ----------------------------------------------------------------------------------------------------------------------------------------------------------------------------------------------------------------------------------MIVRRLTCGGLVHSHLHAN-------------------LKRLQGSQ-----VMARPSSDL**

**5_Danio_RE ----------------------------------------------------------------------------------------------------------------------------------------------------------------------------------------------------------------------------------MIVRQLWCSRGSTSHLCAA-------------------VRLNWRSP-----KMTRPSSDL**

**5_Salm ----------------------------------------------------------------------------------------------------------------------------------------------------------------------------------------------------------------------------------MIVRQFSSRG-VTSHLCAR-------------------FKETQTRQ-----VMARPSSDL**

**5_TaEniop -------------------------------------------------------------------------------------------------------------------------------------------------------------------MSSRCVRRSGHHGSPHPGVKWGGRSPELRARRSRLPSRRPRPAALPLRPSAGRQRPVKHFRGIPMCLFQSAARRLVPQVRCG-------------------LKASSSKKQKFGLEMARPSSNM**

**5_Bos_ta -----------------------------------------------------------------------------------------------------------------------------------------------------------------------------------------------------------------------------------MPPLWIIRNRLFSQLYCG-------------------LKSPVSTQTKICLTMARPSSNM**

**5_Canis_f -----------------------------------------------------------------------------------------------------------------------------------------------------------------------------------------------------------------------------------MQPLQIAPCRLLYGLYRG-------------------LKSPASTGTRICPAMARPSSNM**

**5_Pan_tRog -----------------------------------------------------------------------------------------------------------------------------------------------------------------------------------------------------------------------------------MRPLQIVPGRLISQLYCG-------------------LKPPASTRNQICPKMARPSSSM**

**5_Macac_mu -----------------------------------------------------------------------------------------------------------------------------------------------------------------------------------------------------------------------------------MRPLQIVPSRLISQLYCG-------------------LKPPASTRNQICLKMARPSSSM**

**5_Mus_mus -----------------------------------------------------------------------------------------------------------------------------------------------------------------------------------------------------------------------------------MRPLLIAPGRFISQLCCR-------------------RKPPASPQSKICLTMARPSSNM**

**5_ORnit_an ---------------------------------------------------------------------------------------------------------------------------------------------MSMEVIKRPDLSHAGAGEGYGLSEMGRSAAVARGRWARANSKLQPPMVLRHRHQSLGSPNPCRVLPTSALPFSSGTLPGQCMAPAEQIPMPVTPANLVSRLGGE-------------------LKLPASKRLKICIGMARPSSNM**

**5_Rattus_noR -----------------------------------------------------------------------------------------------------------------------------------------------------------------------------------------------------------------------------------MRPLPVAPGRLFSQLCCG-------------------PKPSASPQSKICLTMARPSSNM**

**5_Sus_sc -----------------------------------------------------------------------------------------------------------------------------------------------------------------------------------------------------------------------------------MPPLWGVPGRLLSQLRCG-------------------LTSPASAPTRICPTMARPSSSM**

**5_HUMAN -----------------------------------------------------------------------------------------------------------------------------------------------------------------------------------------------------------------------------------MRPLQIVPSRLISQLYCG-------------------LKPPASTRNQICLKMARPSSSM**

**SIRT6VITIS ----------------------------------------------------------------------------------------------------------------------------------------------------------------------------------------------------------------------------------MSLGYAEKLSYIEDVGKVGMS--------------------------EICDPLHVLQEKV**

**SIRT6RICINUS -----------------------------------------------------------------------------------------------------------------------------------------------------------------------------------------------------------------------------------MSLGYAEKLSFIEDVGNVGM--------------------------AEFFDSSYVLQEK**

**SIRT6ARAB -----------------------------------------------------------------------------------------------------------------------------------------------------------------------------------------------------------------------------------MSLGYAEKLSFIEDVGQVGM--------------------------AEFFDPSHLLQCK**

**SIRT6ZEAMAYS -----------------------------------------------------------------------------------------------------------------------------------------------------------------------------------------------------------------------------------MSLGYAEKLSYREDVGTVGM--------------------------PEIFETPELVQNK**

**SIRT6TRITICUM -----------------------------------------------------------------------------------------------------------------------------------------------------------------------------------------------------------------------------------MSLGYAEKLSYREDVGTVGM--------------------------PEKFDSPKLLQGK**

**SIRT6ORIZA -----------------------------------------------------------------------------------------------------------------------------------------------------------------------------------------------------------------------------------MSLGYAEKLSYREDVGNVGM--------------------------PEIFDSPELLHKK**

**SIRT6PHYSCOM ----------------------------------------------------------------------------------------------------------------------------------------------------------------------------------------------------------------------------------MSSLGYAEKLSYRADVGTVGM--------------------------PELYDPAEDLQSK**

**6_StRon_p ----------------------------------------------------------------------------------------------------------------------------------------------------------------------------------------------------------------------------------------------------------------------------------------------**

**6_LEpEo_sal ----------------------------------------------------------------------------------------------------------------------------------------------------------------------------------------------------------------------------------MSCSYAEGLSDYANKGKLGLP--------------------------ESFDSPEDLKSKV**

**6_AcyRt_p ----------------------------------------------------------------------------------------------------------------------------------------------------------------------------------------------------------------------------------MSCNYADGLSPYEYKGEVGMN--------------------------EVFDTPEVLKQKI**

**6_Apis_mEl ----------------------------------------------------------------------------------------------------------------------------------------------------------------------------------------------------------------------------------MSCSYADGLSQYENKGVLGLE--------------------------ERYDSVEALRLKC**

**6_Dros_mEl ----------------------------------------------------------------------------------------------------------------------------------------------------------------------------------------------------------------------------------MSCNYADGLSAYDNKGILGAP--------------------------ESFDSDEVVAEKC**

**6_Danio_rE ----------------------------------------------------------------------------------------------------------------------------------------------------------------------------------------------------------------------------------MSVNYAAGLSPYADKGICGLP--------------------------ETFDSPEELKTKV**

**6_XEnop_tR ----------------------------------------------------------------------------------------------------------------------------------------------------------------------------------------------------------------------------------MSVNYAAGLSPYSDKGRCGLP--------------------------EAFDPPDELCRKV**

**6_Gallus ----------------------------------------------------------------------------------------------------------------------------------------------------------------------------------------------------------------------------------MAVNYAAGLSPYSDKGKCGLP--------------------------EIFDPPEELERKV**

**6_Bos_ta ----------------------------------------------------------------------------------------------------------------------------------------------------------------------------------------------------------------------------------MSVNYAAGLSPYADKGKCGLP--------------------------EVFDPPEELEQKV**

**6_Canis_fam ----------------------------------------------------------------------------------------------------------------------------------------------------------------------------------------------------------------------------------MSVNYAAGLSPYADKGKCGLP--------------------------EIFDPPEELERKV**

**6_Mus_mus ----------------------------------------------------------------------------------------------------------------------------------------------------------------------------------------------------------------------------------MSVNYAAGLSPYADKGKCGLP--------------------------EIFDPPEELERKV**

**6_Pan_trogl ----------------------------------------------------------------------------------------------------------------------------------------------------------------------------------------------------------------------------------MSVNYAAGLSPYADKGKCGLP--------------------------EIFDPPEELERKV**

**6_Sus_sc ----------------------------------------------------------------------------------------------------------------------------------------------------------------------------------------------------------------------------------MSVNYAAGLSPYADKGKCGLP--------------------------EVFDPPEELEQKV**

**6_Macaca_mu ----------------------------------------------------------------------------------------------------------------------------------------------------------------------------------------------------------------------------------MSVNYAAGLSPYADKGKCGLP--------------------------EIFDPPEELERKV**

**6_Rattus_no ----------------------------------------------------------------------------------------------------------------------------------------------------------------------------------------------------------------------------------MSVNYAAGLSPYADKGKCGLP--------------------------EIFDPPEELECKV**

**6_HUMAN ----------------------------------------------------------------------------------------------------------------------------------------------------------------------------------------------------------------------------------MSVNYAAGLSPYADKGKCGLP--------------------------EIFDPPEELERKV**

**SIRT7PHYSCOM ------------------------------------------------------------------------------------------------------------------------------------------------------------MWKQWIQTTRSQMRTHG-LQKSTRVLFDGHKVLLFKDELRRHEGIHEKLIDSAC-E--AAGGAQECGCQGAEIPCGRLAKCWVALTERGRE------------------------AQEMEDEEEGPVLRA**

**7_Ciona_intE -----------------------------------------------------------------------------------------------------------------------------------------------------MQSVFLPATECGSSDDVFIGADNEEKEVEDEVATPRTLRPRKLAPTVTKRDLTKQIREVVARRHHSLDEKLFLDEHCSLVKAVRARASSYSKLKQRSQ--------------------------EVFDDPETLHAKC**

**7_Apis_mEl ---------------------------------------------------------------------------------------------------------------------------------------------------------------------MEETSNEKFLSRR-RSAALKAFKVKDE-RVATFKKVAAILQKSETDR--TAEETGILISCSDVVKEVNLRQEKRHRVKARLE--------------------------EIEDAPELLEEKC**

**7_Danio_RE ---------------------------------------------------------------------------------------------------------------------------------------------------------------------MDVRINSGVSARAERKEQEKAKIIQREKQRQTMKTISKILQKCESEW--TEEERSMLQAHQDTVQELSRRQNRRHLLKRKQE--------------------------EVFDDAENLKTKV**

**7_XEnopus_t ------------------------------------------------------------------------------------------------------------------------------------------------------------------------------MGRAERKAEAREEILRREQNRDRLRRVCVILHKPAADR--SPEESELLGKCGDLVHELEKRRLHVERRRHREQ--------------------------EVLDDTDLLREKV**

**7_Bos_ta ------------------------------------------------------------------------------------------------------------------------------------------------------------------------MAAGG-LSRSERKAAERVRRLREEQQRERLRQVSRILRKAATER--SAEEGRLLAESEDLVTELQGRSRRREGLKRRQE--------------------------EVCDDPEELQRKV**

**7_Canis_fa ------------------------------------------------------------------------------------------------------------------------------------------------------------------------MAAGG-PSRSERKAAERVRRLREEQQRERLRQVSRILRKAVAER--SAEEGRLLAESEDLVTELQGRSRRREGLKRRQE--------------------------EVCDDPEELRRKV**

**7_Equus_cab -----------------------------------------------------------------------------------------------------------------------------------------------------------------------------------------MPYCRSVERRE----------KAGKAG--PAP----WSRSENLVTELQAEQRR--GLKRRQE--------------------------EVCDDPEELRRKV**

**7_Macaca ------------------------------------------------------------------------------------------------------------------------------------------------------------------------MAAGG-LSRSERKAAERVRRLREEQQRERLRQVSRILRKAAAER--SAEEGRLLAESADLVTELQGRSRRREGLKRRQE--------------------------EVCDDPEELRGKV**

**7_Mus_mus -------------------------------------------------------------------------------------------------------------------------------------------------------------------SAVKGAMAAGGGLSRSERKAAERVRRLREEQQRERLRQVSRILRKAAAER—SAEEGRLLAESEDLVTELQGRSRRREGLKRRQEEASRGQRVLGGGLREASGGNNQPGSYQVCDDPEELRRKV**

**7_Pan_t ------------------------------------------------------------------------------------------------------------------------------------------------------------------------MAAGG-LSRSERKAAERVRRLREEQQRERLRQVSRILRKAAAER--SAEEGRLLAESADLVTELQGRSRRREGLKRRQE--------------------------EVCDDPEELRGKV**

**7_aRattus ------------------------------------------------------------------------------------------------------------------------------------------------------------------------MAAGGGLSRSERKAAERVRRLREEQQRERLRQVSRILRKAAAER--SAEEGRLLAESEDLVTELQGRSRRREGLKRRQE--------------------------EVCDDPEELRRKV**

**7_Sus_sc ------------------------------------------------------------------------------------------------------------------------------------------------------------------------MAAGG-LSRSERKAAERVRRLREEQQRERLRQVSRILRKAAAER--SAEEGRLLAESEDLVTELQGRSRRREGLKRRQE--------------------------EVCDDPEELRRKV**

**7_HUMAN ------------------------------------------------------------------------------------------------------------------------------------------------------------------------MAAGG-LSRSERKAAERVRRLREEQQRERLRQVSRILRKAAAER--SAEEGRLLAESADLVTELQGRSRRREGLKRRQE--------------------------EVCDDPEELRGKV**

**D)SIRTUINS – CARBOXY TERMINAL SEGMENTS: FIRST PART starting from the core on the right side of the table to the end(at right)**

**A)NEGATIVE SEQUENCE STRETCHS IN RED,**

**B) POSITIVE SEQUENCE STRETCHS IN LIGHT BLUE,**

**C) PREDICTED PHOSPHORYLATION SITES IN GREEN**

**1.Hydra_magnipapillata DVIGT-------------YKP-----------------------------------------------------------------------------------------------------------------------------------------------------------------SFLSVSKTIKTFSNTSD--------SNKSLSGFDK-----------------------------------------------------------------------ECYNSSN------------------------------------------HVLQDQ-**

**1.Strongylocentrotus HVCQSQQRLTETTHIPNGFKPKESRQARKAVLEEGGTTVDQRPDPVLDEGETTVDQRPDPVLDEGETTVDQRSNPVLDEGETTVDQKPDPVLDEGETTVDQKPDPVLDEGETTVDQKPDPVLDEGETTVDQKPDPAGRIDQEESVSNP---------------------------------VNVKSKEDTQHSLSSLKE--------EGQVSSGDDKNEGSKAGEGDDRLARSNSQDSPISPEGHVKQSCDERLQAPENQTSLEHHINGLTNEGKIHVDALPCSRSETNCSPSSNSDRLQSVDEVENALASSEPQTSKTIPSESSQS----------HTAQDQT**

**1.Brugia_malayi DIFRKGRFKTNSND-----------------------------------------------------------------------------------------------------------------------------------------------------------------------------PTAIEEIYEKTT---------------------------------------------------------------------------------------LRKAINEIQFKKLLDQPTVKRARLKEG-------------------------------**

**1.aSchistosoma_mansoni GLSDYTP--------------------------------------------------------------------------------------------------------------------------------------------------------------------------------LTEIPLNSLKNTTEPL-----------KADEKSPNVS-------------------------------------------------------------------HLIVQDTVSSELVNDESQRNDNSEVN-------------------------------**

**1.Schistosoma_japonicum --------------------------------------------------------------------------------------------------------------------------------------------------------------------------------------------------------------------------------------------------------------------------------------------------------------------------------------------------------**

**1.Acyrthosiphon_pisum ELCWCKEELTEAKT----LNTPT--------------------------------------------------------------------------------------------------------------------------------------------------------------SSPRSNEATIETVEG--------------------------------------------------------------------------------------------EMSTDSTRDSANSMSPP---------LGDDRFTPG---------------TSGEQR**

**1.Tribolium_castaneum EGVFDDNMLEEASH----LLPLQEPSFD---------------------------------------------------------------------------------------------------------------------------------------------------------MTPPCVKSQTEPNCTDD------------NLEKASPCLCS------------------------------------------------------------------TKCSSSSTVEKDEELTLECSCNKQTSIINKQSLLDSDLVN------SLDLKSVKER-**

**1.Apis_mellifera EVCWNDTILKETTQ----LLALRYITDD---------------------------------------------------------------------------------------------------------------------------------------------------------TWEQSQDTSSNTILSRDSVEINFKAHDPQSIESQDNLMIHNNI---------------------------------------------------VEHYEENMNARISPFCNDHTNSIEEKFNLLGESPKRR--LGESSIESSPKRMNFGSNCVLHFNTCPSKT**

**1.Pediculus ELCWRDGKLVEATE----LLPVS--TDE---------------------------------------------------------------------------------------------------------------------------------------------------------SNNFKLDSEAENLTS--------------QINSQTEIFVG----------------------------------------------------------------KVSNQRCGHT-VQESKTDCNENCRKRS--LGTSDCPGNDSKR---------QKLTEEKT**

**1.Danio_Rerio QLCYNSSRLSEITE-----KPAAPEHTEN--------------------------------------------------------------------------------------------------------------------------------------------------------TSADHSHADAEHIEN-------------TSADHSHADAE-------------------------------------------------------------------HIENTS-ADR--DDAKHTENTPTDHA-----------DAE----------HTKNTSA**

**1.aNothobranchius_f QLCFNTLRLNEITE-----KPPRLQEPRP--------------------------------------------------------------------------------------------------------------------------------------------------------SEALPAFSDAAQQQNEPS--------RTDSVNKPSEEAE-------------------------------------------------------------------SLTVTETADKNITPLEPCSNVQCPSE-----------DGA----------KSPELPK**

**1.bNothobranchius_K QLCFNTLRLNEITE-----KPPRLQEPRP--------------------------------------------------------------------------------------------------------------------------------------------------------SEALPAFSDAAQQQNEPS--------RTDSVNKPSEEAE-------------------------------------------------------------------SLTVTETADKNITPLEPCSNVQCPSE-----------DGA----------KSPELPK**

**1.Xenopus_tropicalis QLCTNSLKLSQITE-----KPPRIHKGFL--------------------------------------------------------------------------------------------------------------------------------------------------------TSPETVPSTDLNTGQ--S--------PALQSDLRGTDLQ-------------------------------------------------------------------LSASNTMRSLEKPEEASKLSHNCSEE-----NLEVSKEAN----------TQLSNEK**

**1.Taeniopygia_guttata KLCYNSVKLSEITE-----KPPRPHKELE--------------------------------------------------------------------------------------------------------------------------------------------------------ALSAELPPTPLNISEGSS--------SPERMSP-ANSAR-------------------------------------------------------------------ASEHPPECKVENCQPAPETTGTCSEE--TLQDTQVSSENP----------ENPASEL**

**1.Gallus_gallus KLCYNSVKLSEITE-----KPPRMHKELE--------------------------------------------------------------------------------------------------------------------------------------------------------MHSSELPPTPLDISEDSG--------SPEQMTPPGTSVV-------------------------------------------------------------------PSEHAAECKVENSDPASETKGICTEE--KLQDTQASSENP----------ENPASEL**

**1.Equus_caballus KLCCNPIKLSEITE-----KPPRTQREL---------------------------------------------------------------------------------------------------------------------------------------------------------AHLSELPPTPLNISEDSS--------SPERTSPPDSSVI-------------------------------------------------------------------VTLLDQATKSNVDDPDVSKSKDCMEE--KSQEVQTSTRSF----------QSVTERL**

**1.Macaca_mulatta KLCCNPVKLSEITE-----KPPRTQKEL---------------------------------------------------------------------------------------------------------------------------------------------------------AYLSDLPPTPLHISEDSS--------SPERTSPPDSSVI-------------------------------------------------------------------VTLLDQAAKSN-DDLDVSESKGRMEE--KPQEVQTS-RNV----------ESIAEHM**

**1.Monodelphis_Domestica RLCSNPTRLSEITE-----KPPRPQRELG--------------------------------------------------------------------------------------------------------------------------------------------------------ARWGALPPTPLHVSEDSS--------SPGRTSPPDSWAG-------------------------------------------------------------------ERRAGEADDG-------AASRGSCRADKPPPE-----VHP----------ASITEQL**

**1.Ornithorhynchus QLCSNPVKLSEITE-----KPPRTHKELE--------------------------------------------------------------------------------------------------------------------------------------------------------ARWAALPPTPLHVSEGSS--------SPDRTSPPDALAA-------------------------------------------------------------------PARPEQMTDSRVVQVESGAEAGGCPGGAAPQAGQSSSESA----------AAVSQQL**

**1.Canis_familiaris KLCCNPVKLSEITE-----KPPRTQKEL---------------------------------------------------------------------------------------------------------------------------------------------------------AHLSELPPTPLNISEDSS--------SPERTSPPDSSVI-------------------------------------------------------------------VTLLDEATKSNVDDPGVSESRDCMEE--KSQEGQNSIRNI----------ESVTEHL**

**1.Bos_taurus KLCCNPVKLSEITE-----KPPRIQKEL---------------------------------------------------------------------------------------------------------------------------------------------------------AHLSELPPTPLNISEGSS--------SPERTSPPDSSVI-------------------------------------------------------------------VTLLDQETKSNVDDPDVSESKDHVTE--KSQEVQTSTRSI----------ESVNEQL**

**1.Sus_scrofa KLCCNPVKLSEITE-----KPPRTQKEL---------------------------------------------------------------------------------------------------------------------------------------------------------AHLSELPPTPLNISEGSS--------SPERTSPPASSVT-------------------------------------------------------------------LPLLDQATKSNVDDLDVSESKDCVEE--KSQEVQTS-RSI----------ESVKEPM**

**1.Rattus_norvegicus KLCCNPVKLSEITE-----KPPRTQKEL---------------------------------------------------------------------------------------------------------------------------------------------------------VHLSELPPTPLHISEDSS--------SPERTVPQDSSVI-------------------------------------------------------------------ATLVDQTIKNKVDDLEVSEPKSCVEE--KSQEVQTY-RNV----------ESIN--V**

**1.Mus_musculus KLCCNPVKLSEITE-----KPPRPQKEL---------------------------------------------------------------------------------------------------------------------------------------------------------VHLSELPPTPLHISEDSS--------SPERTVPQDSSVI-------------------------------------------------------------------ATLVDQATNNNVNDLEVSES-SCVEE--KPQEVQTS-RNV----------ENIN--V**

**1.SIRT1_HUMAN KLCCNPVKLSEITE-----KPPRTQKEL---------------------------------------------------------------------------------------------------------------------------------------------------------AYLSELPPTPLHVSEDSS--------SPERTSPPDSSVI-------------------------------------------------------------------VTLLDQAAKSN-DDLDVSESKGCMEE--KPQEVQTS-RNV----------ESIAEQM**

530 538/539 540 544/545

**2_aAjellomyces_er ----DDVLLLGECD------------------------------------------------------------------------------------------------------------------------------------------------------------------------------DGVLKLAEALG-------------------------------------------------------------------------------------------------------------------------------------------------**

**2_aMicromonas_CCMP1545 D-NRRDALFLGDCD------------------------------------------------------------------------------------------------------------------------------------------------------------------------------AGFAELARLLG-------------------------------------------------------------------------------------------------------------------------------------------------**

**2_Ostreococcus_tauri DTNYRDALYLGACD------------------------------------------------------------------------------------------------------------------------------------------------------------------------------DGIAELSELLG-------------------------------------------------------------------------------------------------------------------------------------------------**

**2_Trichoplax_adhaerens ESNYRDVFWQGTCD------------------------------------------------------------------------------------------------------------------------------------------------------------------------------DGCFTLAEALG-------------------------------------------------------------------------------------------------------------------------------------------------**

**2_Nematostella_vectensis EDNYRDVAWLGTTD------------------------------------------------------------------------------------------------------------------------------------------------------------------------------DGCLALAELLG-------------------------------------------------------------------------------------------------------------------------------------------------**

**2_Branchiostoma ENNYRDVAWLGDCD------------------------------------------------------------------------------------------------------------------------------------------------------------------------------EGCKALAELLG-------------------------------------------------------------------------------------------------------------------------------------------------**

**2_Strongylocentrotus DDKYRDVAYIGPCD------------------------------------------------------------------------------------------------------------------------------------------------------------------------------EGCEKLAGFVG-------------------------------------------------------------------------------------------------------------------------------------------------**

**2_Ciona_intestinalis DKAYRDVCWLGDCD------------------------------------------------------------------------------------------------------------------------------------------------------------------------------DGCLALADLLG-------------------------------------------------------------------------------------------------------------------------------------------------**

**2_Brugia_malayi KDNYRDVFWQGTTD------------------------------------------------------------------------------------------------------------------------------------------------------------------------------DGAWKLAELLG-------------------------------------------------------------------------------------------------------------------------------------------------**

**2_aSchistosoma_japonicum PGNKRDVFWSGNTD------------------------------------------------------------------------------------------------------------------------------------------------------------------------------DGVVKISELVG-------------------------------------------------------------------------------------------------------------------------------------------------**

**2_Schistosoma_mansoni SDNKRDIFWSGNAD------------------------------------------------------------------------------------------------------------------------------------------------------------------------------DGVVKISELLG-------------------------------------------------------------------------------------------------------------------------------------------------**

**2_aCaligus_clemensi DRRYRDVAEIGKCD------------------------------------------------------------------------------------------------------------------------------------------------------------------------------DGCKKLAEALG-------------------------------------------------------------------------------------------------------------------------------------------------**

**2_Lepeophteeirus DRRYRDVAEIGTCD------------------------------------------------------------------------------------------------------------------------------------------------------------------------------DGCKKLAEALG-------------------------------------------------------------------------------------------------------------------------------------------------**

**2_Aedes_aegypti PGNRRDVAWTGDCD------------------------------------------------------------------------------------------------------------------------------------------------------------------------------DGCFFLADKLG-------------------------------------------------------------------------------------------------------------------------------------------------**

**2_Apis_mellifera SNGARDVAWLGECD------------------------------------------------------------------------------------------------------------------------------------------------------------------------------IGCQLLADKLG-------------------------------------------------------------------------------------------------------------------------------------------------**

**2_Bombyx_mori EGSYRDVARLGDCD------------------------------------------------------------------------------------------------------------------------------------------------------------------------------EGCQDLADRLG-------------------------------------------------------------------------------------------------------------------------------------------------**

**2_Drosophila PNNTRDVAFLGDCD------------------------------------------------------------------------------------------------------------------------------------------------------------------------------AGVMALAKALG-------------------------------------------------------------------------------------------------------------------------------------------------**

**2_Pediculus DKNYRDVAFLGDCD------------------------------------------------------------------------------------------------------------------------------------------------------------------------------ELCEQLVDLLG-------------------------------------------------------------------------------------------------------------------------------------------------**

**2_Tribolium_castaneum KNNTRDVAWIGDCD------------------------------------------------------------------------------------------------------------------------------------------------------------------------------EGCQLLADKLG-------------------------------------------------------------------------------------------------------------------------------------------------**

**2_Danio_Rerio DKAYRDVAHLSTCD------------------------------------------------------------------------------------------------------------------------------------------------------------------------------DGCMTLAELLG-------------------------------------------------------------------------------------------------------------------------------------------------**

**2_Salmo_salar --------------------------------------------------------------------------------------------------------------------------------------------------------------------------------------------------------------------------------------------------------------------------------------------------------------------------------------------------------**

**2_Xenopus_laevis EKAYRDVAWLGDCD------------------------------------------------------------------------------------------------------------------------------------------------------------------------------DGCLALADFLG-------------------------------------------------------------------------------------------------------------------------------------------------**

**2_Gallus_gallus DKAYRDVAWLGDCD------------------------------------------------------------------------------------------------------------------------------------------------------------------------------EGCLALAELLG-------------------------------------------------------------------------------------------------------------------------------------------------**

**2_Bostaurus KKAYRDVAWLGDCD------------------------------------------------------------------------------------------------------------------------------------------------------------------------------QGCLALADLLG-------------------------------------------------------------------------------------------------------------------------------------------------**

**2_Canis_familiaris KKAYRDVAWLGDCD------------------------------------------------------------------------------------------------------------------------------------------------------------------------------QGCLALADLLG-------------------------------------------------------------------------------------------------------------------------------------------------**

**2_Equus_caballus KKAYRDVAWLGDCD------------------------------------------------------------------------------------------------------------------------------------------------------------------------------QGCL-LADLLG-------------------------------------------------------------------------------------------------------------------------------------------------**

**2_Caligus_Rogercresseyi ERRYRDVAEIGSCD------------------------------------------------------------------------------------------------------------------------------------------------------------------------------AGCSKLAEALG-------------------------------------------------------------------------------------------------------------------------------------------------**

**2_Rattus_norvegicus KKAYRDVAWLGDCD------------------------------------------------------------------------------------------------------------------------------------------------------------------------------QGCLALADLLG-------------------------------------------------------------------------------------------------------------------------------------------------**

**2_Mus_musculus KKAYRDVAWLGDCD------------------------------------------------------------------------------------------------------------------------------------------------------------------------------QGCLALADLLG-------------------------------------------------------------------------------------------------------------------------------------------------**

**2_Sus_scRofa KKAYRDVAWLGDCD------------------------------------------------------------------------------------------------------------------------------------------------------------------------------QGCLALADLLG-------------------------------------------------------------------------------------------------------------------------------------------------**

**2_HUMAN KKAYRDVAWLGECD------------------------------------------------------------------------------------------------------------------------------------------------------------------------------QGCLALAELLG-------------------------------------------------------------------------------------------------------------------------------------------------**

**3_Ciona_intEstinalis -----DLAVTGDIM------------------------------------------------------------------------------------------------------------------------------------------------------------------------------DSIQTFVDELG-------------------------------------------------------------------------------------------------------------------------------------------------**

**3_XEnopus_tRopicalis -----DVAELGDLC------------------------------------------------------------------------------------------------------------------------------------------------------------------------------DIIHTMVSRLS-------------------------------------------------------------------------------------------------------------------------------------------------**

**3_Danio_RERioB -----DYMELGDLS------------------------------------------------------------------------------------------------------------------------------------------------------------------------------ESVRKLAEILG-------------------------------------------------------------------------------------------------------------------------------------------------**

**3_Gallus_gallus -----DIAQLGDVV------------------------------------------------------------------------------------------------------------------------------------------------------------------------------TGVEKMVELLD-------------------------------------------------------------------------------------------------------------------------------------------------**

**3_TaEniopygia_guttata -----DVAQLGDVV------------------------------------------------------------------------------------------------------------------------------------------------------------------------------SGVEKLVELLG-------------------------------------------------------------------------------------------------------------------------------------------------**

**3_Equus_caballus -----DVVQLGDVV------------------------------------------------------------------------------------------------------------------------------------------------------------------------------HGVEKLVELLG-------------------------------------------------------------------------------------------------------------------------------------------------**

**3_iso9Canis_familiaRis -----DVVQLGDVV------------------------------------------------------------------------------------------------------------------------------------------------------------------------------HSVERLVELLG-------------------------------------------------------------------------------------------------------------------------------------------------**

**3_Mus_musculus -----DVVQLGDVV------------------------------------------------------------------------------------------------------------------------------------------------------------------------------HGVERLVDLLG-------------------------------------------------------------------------------------------------------------------------------------------------**

**3_aRattus_noRvEgicus -----DVVQLGDVV------------------------------------------------------------------------------------------------------------------------------------------------------------------------------QGVERLVDLLG-------------------------------------------------------------------------------------------------------------------------------------------------**

**3_ORyctolagus_cuniculus -----DVAQLGDVV------------------------------------------------------------------------------------------------------------------------------------------------------------------------------HGVGKLVELLG-------------------------------------------------------------------------------------------------------------------------------------------------**

**3_MonoDElpHis_DomEstica -----DVAQLGDII------------------------------------------------------------------------------------------------------------------------------------------------------------------------------GGVEMLVKALG-------------------------------------------------------------------------------------------------------------------------------------------------**

**3_Macaca_mulatta -----DVAKQLFCI------------------------------------------------------------------------------------------------------------------------------------------------------------------------------CV----------------------------------------------------------------------------------------------------------------------------------------------------------**

**3_Sus_scRofa -----DVVQLGDLV------------------------------------------------------------------------------------------------------------------------------------------------------------------------------HGVKRLVELLG-------------------------------------------------------------------------------------------------------------------------------------------------**

**3_HUMAN -----DVAQLGDVV------------------------------------------------------------------------------------------------------------------------------------------------------------------------------HGVESLVELLG-------------------------------------------------------------------------------------------------------------------------------------------------**

**4_Stigmatella_DW4/3 --------------------------------------------------------------------------------------------------------------------------------------------------------------------------------------------------------------------------------------------------------------------------------------------------------------------------------------------------------**

**4_Strongylocentrotus --------------------------------------------------------------------------------------------------------------------------------------------------------------------------------------------------------------------------------------------------------------------------------------------------------------------------------------------------------**

**4_Acyrthosiphon_pisum --------------------------------------------------------------------------------------------------------------------------------------------------------------------------------------------------------------------------------------------------------------------------------------------------------------------------------------------------------**

**4_Apis_mellifera --------------------------------------------------------------------------------------------------------------------------------------------------------------------------------------------------------------------------------------------------------------------------------------------------------------------------------------------------------**

**4_Drosophila VS------------------------------------------------------------------------------------------------------------------------------------------------------------------------------------------------------------------------------------------------------------------------------------------------------------------------------------------------------**

**4_TRibolium_castaneum --------------------------------------------------------------------------------------------------------------------------------------------------------------------------------------------------------------------------------------------------------------------------------------------------------------------------------------------------------**

**4_Danio_Rerio --------------------------------------------------------------------------------------------------------------------------------------------------------------------------------------------------------------------------------------------------------------------------------------------------------------------------------------------------------**

**4_Xenopus_tropicalis EIQS----------------------------------------------------------------------------------------------------------------------------------------------------------------------------------------------------------------------------------------------------------------------------------------------------------------------------------------------------**

**4_Gallus_gallus --------------------------------------------------------------------------------------------------------------------------------------------------------------------------------------------------------------------------------------------------------------------------------------------------------------------------------------------------------**

**4_Taeniopygia_guttata --------------------------------------------------------------------------------------------------------------------------------------------------------------------------------------------------------------------------------------------------------------------------------------------------------------------------------------------------------**

**4_Mouse VQRLEMNFPLSSAAQDP---------------------------------------------------------------------------------------------------------------------------------------------------------------------------------------------------------------------------------------------------------------------------------------------------------------------------------------**

**4_Bos_tauRus --------------------------------------------------------------------------------------------------------------------------------------------------------------------------------------------------------------------------------------------------------------------------------------------------------------------------------------------------------**

**4_Equus_caballus --------------------------------------------------------------------------------------------------------------------------------------------------------------------------------------------------------------------------------------------------------------------------------------------------------------------------------------------------------**

**4_Pan_troglodytes --------------------------------------------------------------------------------------------------------------------------------------------------------------------------------------------------------------------------------------------------------------------------------------------------------------------------------------------------------**

**4_Canis_familiaris --------------------------------------------------------------------------------------------------------------------------------------------------------------------------------------------------------------------------------------------------------------------------------------------------------------------------------------------------------**

**4_Ovis_aries --------------------------------------------------------------------------------------------------------------------------------------------------------------------------------------------------------------------------------------------------------------------------------------------------------------------------------------------------------**

**4_Macaca_mulatta --------------------------------------------------------------------------------------------------------------------------------------------------------------------------------------------------------------------------------------------------------------------------------------------------------------------------------------------------------**

**4_Mus_musculus VQRLEMNFPLSSAAQDP---------------------------------------------------------------------------------------------------------------------------------------------------------------------------------------------------------------------------------------------------------------------------------------------------------------------------------------**

**4_Rattus_norvegicus --------------------------------------------------------------------------------------------------------------------------------------------------------------------------------------------------------------------------------------------------------------------------------------------------------------------------------------------------------**

**4_Sus_scrofa --------------------------------------------------------------------------------------------------------------------------------------------------------------------------------------------------------------------------------------------------------------------------------------------------------------------------------------------------------**

**4_HUMAN --------------------------------------------------------------------------------------------------------------------------------------------------------------------------------------------------------------------------------------------------------------------------------------------------------------------------------------------------------**

**5_Perkinsus_ATCC --------------------------------------------------------------------------------------------------------------------------------------------------------------------------------------------------------------------------------------------------------------------------------------------------------------------------------------------------------**

**5_Ciona_intestinalis --------------------------------------------------------------------------------------------------------------------------------------------------------------------------------------------------------------------------------------------------------------------------------------------------------------------------------------------------------**

**5_Apis_mellifera --------------------------------------------------------------------------------------------------------------------------------------------------------------------------------------------------------------------------------------------------------------------------------------------------------------------------------------------------------**

**5_Nasonia_vitripennis --------------------------------------------------------------------------------------------------------------------------------------------------------------------------------------------------------------------------------------------------------------------------------------------------------------------------------------------------------**

**5_Anoplopoma_fimbria TRVLDISCTQTTLSNTGGLYWYKYQKYLVDYNEQCGDGVLDK--------------------------------------------------------------------------------------------------------------------------------------------------------------------------------------------------------------------------------------------------------------------------------------------------------------**

**5_Danio_Rerio V-------------------------------------------------------------------------------------------------------------------------------------------------------------------------------------------------------------------------------------------------------------------------------------------------------------------------------------------------------**

**5_Salmo_salar --------------------------------------------------------------------------------------------------------------------------------------------------------------------------------------------------------------------------------------------------------------------------------------------------------------------------------------------------------**

**5_Taeniopygia_guttata IS------------------------------------------------------------------------------------------------------------------------------------------------------------------------------------------------------------------------------------------------------------------------------------------------------------------------------------------------------**

**5_Bos_taurus VS------------------------------------------------------------------------------------------------------------------------------------------------------------------------------------------------------------------------------------------------------------------------------------------------------------------------------------------------------**

**5_Canis_familiaris VS------------------------------------------------------------------------------------------------------------------------------------------------------------------------------------------------------------------------------------------------------------------------------------------------------------------------------------------------------**

**5_Pan_troglodytes VS------------------------------------------------------------------------------------------------------------------------------------------------------------------------------------------------------------------------------------------------------------------------------------------------------------------------------------------------------**

**5_Macaca_mulatta VS------------------------------------------------------------------------------------------------------------------------------------------------------------------------------------------------------------------------------------------------------------------------------------------------------------------------------------------------------**

**5_Mus_musculus TS------------------------------------------------------------------------------------------------------------------------------------------------------------------------------------------------------------------------------------------------------------------------------------------------------------------------------------------------------**

**5_Ornithorhynchus VS------------------------------------------------------------------------------------------------------------------------------------------------------------------------------------------------------------------------------------------------------------------------------------------------------------------------------------------------------**

**5_Rattus_norvegicus IS------------------------------------------------------------------------------------------------------------------------------------------------------------------------------------------------------------------------------------------------------------------------------------------------------------------------------------------------------**

**5_Sus_scrofa VS------------------------------------------------------------------------------------------------------------------------------------------------------------------------------------------------------------------------------------------------------------------------------------------------------------------------------------------------------**

**5_HUMAN VS------------------------------------------------------------------------------------------------------------------------------------------------------------------------------------------------------------------------------------------------------------------------------------------------------------------------------------------------------**

SIRT6VITIS PFVRIDLFQVILTHTLSSDKRFV NWILR-----------------------------------------------VASV--HGQKAPLPF--------------------------------IKYVEVSFLDGQNYK**----**EA**------------------------**VLHK--QPFQLKRRTVKTKIFEVLLKLNFSDGCGCLSSQIKVPIDFKVSTDCFN--YDKDAILQKLRDTATGDPCCGRHEVIEKKPIPDPRSEATVYAIVTNVLQYN-KTAPESNGSVMKGRLGGLNGIE-----TSWKRSRSGK-------------------------------

SIRT6RICINUS PYVRIDLLQIIVTRSLSADKRFV NWTLR-----------------------------------------------IASV--HALKATLPF--------------------------------IKSIEVTFSDTQKYK**----**AA**------------------------**ILHE--QPFNLKRRTVTTESFEIFLKLNLSDGCGCLCTQINIPFGFKVLNDCFN--LKKDSVIQNLREKAIQVLGCGQNAMIERKTIIAPRSEVTVHAIVTNIKAFE-SDG-LSNGEVKRLRGSSINGIM-----TCRKRSNSRK-------------------------------

SIRT6ARAB PYVRIDLFQIILTQSISGDQRFI NWTLR-----------------------------------------------VASV--HGLTSQLPF--------------------------------IKSIEVSFSDNHNYK**----**DA**------------------------**VLDK--QPFLMKRRTARNETFDIFFKVNYSDGCDCVSTQLSLPFEFKISTEEHVEIIDKEAVLQSLREKAVEESSCGQSGVVERRVVSEPRSEAVVYATVTSLRTYHSQQSLLANGDLKWK----LEGSG-----TSRKRSRTGK-------------------------------

SIRT6ZEAMAYS PYIRTDFVQLTLRHSLK--KKCV RWTLR-----------------------------------------------VTSI--HGLRAPLPF--------------------------------LQSVKVSFPERPDLK**----**SV**------------------------**VLKE--QPFSLQRETSMNKPFFMLLTLNFSDGCSCLSSSIGWPVDFQKRKDSFV--RDRALVLRELYSAAQRESCIGQQEILERENL--PRAETSIHGIVTNIVRYDTEDEKLAPPKNDLMNHSRSNPAKRHVEGTDCHSSLPKK-------------------------------

SIRT6TRITICUM PYIRTDFIQLLLRHTVK--KKCV RWTLR-----------------------------------------------VTSV--HGMRAPLSF--------------------------------LRSIEVSFPDRSDMK**--------**PVVLME--QPFSLQRETSMTSIFSMLLTLKFSDGCGNH----------------------------------------------------------------------------------------------------------------------------------------------------------------

SIRT6ORIZA PYIRTDFVQISLRNSVK--KKCV RWTLR-----------------------------------------------VTSI--HGLRAPLPF--------------------------------LRSVEVSFPERPDMKPVVLKE**------------------------**--QPFSLQRETSMNRPFVMLLTFNFSDGCGCSSSSIEWPVDFLKQKDSFV--RDRSLVLQELQHAAEHRSRAGQHAILEREGV--PRAETSIHALVTNIVRYDTEDSKAAVPMATWMN-SNGSLSKRHMDAIGCNPASSKK-----------------------------------

SIRT6PHYSCOM PYVHIDRILLSYYYYWTK-KKSV KWYFR-----------------------------------------------ISSI--HGQKMALPF--------------------------------IKSIEVMFPNRPEFK**----**PA**------------------------**AFAK--PPCLVRRETMRLKELDVALKLHFAEGCMCSSGDIFQTLSFEVRDNYLLCRID----------------------------------------------------------------------------------------------------------------------

**6_Strongylocentrotus PEYTGPSLVLESQQGLSTKNIKDTMHVGDSQKNCKVDSECVDDRRKVAVKDEVKEESMNDGQKVEIKDEVKEKVQKERVSEDLEVSPEMKR-------------------------------EAHLNSHETPDQKVEQSCIRLE------------------------SIEDRQKVAVKDEVKESCLSDGQKVETKDEVKEKVQKERVSEDLEVPPEVKSEAHLDSHETPQENSEQSCKRLCPDTSEVIQDDVAKDTEKHEASDNSFRTCKHEDDDMRETVDIQETSCLVGSDSKQTFENMCSVKQEIHVKSGSADTTDIKPGCPDNWSDSKRTDDEESVENIT**

**6_Lepeophtheirus PEYDKEIDPIRMMD--------------------------------------------------------------KSKDP-NFFIDWTQS-------------------------------EKEAKKIIIKSDRLEDALKMKR------------------------KKDREVTAKLASKKNKSLCDDEEEKNNRGEVLNNIFILKKEETDHCNGENASSFELKNEKDL------------------------------------------------------------------------------------------------------------------**

**6_Acyrthosiphon_pisum --------------------------------------------------------------------------------------------------------------------------------------------------------------------------------------------------------------------------------------------------------------------------------------------------------------------------------------------------------**

**6_Apis_mellifera PEYESTMDPTRNSDTTS-----------------------------------------------------------KEMDW-TIPTSRIKE-------------------------------MNVLYKKVCKPMRRKRKTFMYE------------------------RERTDTKRETKTKKQAFMIKQDIKTEDTMNTANQICNNAVVSEDISSNTVKIEDEIKHVEPFEFTTNNMTQPDPGLEVNNIL----------------------------------------------------------------------------------------------**

**6_Drosophila PEYSEASDPTKQS---------------------------------------------------------------KPMEW-TIPTSNVNT-------------------------------FHRQYKKYVY--------FIY-----------------------------------------YLL-------------------------------------------------------------------------------------------------------------------------------------------------------------**

**6_Danio_Rerio PEWAGPTLCEDSGGDLD----------------------------------------------------------ILPYG---AWKKEVKI-------------------------------ELKIEESKHTVSKKRK------------------------------RKEQHAEEDYKNGVKVEEEMKEEGKESDSHVHTHT---------------------------------------------------------------------------------------------------------------------------------------------**

**6_Xenopus_tropicalis PVWTG-------MPTKT-----------------------------------------------------------EPTN----GNYKEEN-------------------------------HFYNDSVLGANPNQKR-------------------------------------------EGCKEEPNLEPKKAKVEPACV----------------------------------------------------------------------------------------------------------------------------------------------**

**6_Gallus_gallus PEWTGPVVVESADSAKPEQ-----------------------------------------------------LYTFKPEA---HGLLKEEP-------------------------------FSQHNGTAGQCPDLGT-------------------------------TLVEHRDSLKQECPSPDTGPPLTKKMKVEPLLT----------------------------------------------------------------------------------------------------------------------------------------------**

**6_Bos_taurus PAWDGPHMVERALPPLP-----------------------------------------------------------RPPA--PKLEPKEEA-------------------------------SPQLNSPVPANPKQEPTAEPCT------------------------QHNGSGPTSPKRERPDSPSPHRPPKRVKTEVVPS----------------------------------------------------------------------------------------------------------------------------------------------**

**6_Canis_familiaRis PAWDGPRVLERALPPLP-----------------------------------------------------------RPPAKPPEPEPKEEA-------------------------------PAQLNGPAPASPKQEPSTEPCT------------------------QHNGSGPGSPKRERLDSPVPHRPPKRVKAEVAPS----------------------------------------------------------------------------------------------------------------------------------------------**

**6_Mus_musculus PAWDGPCVLDKALPPLP-----------------------------------------------------------RPVA------LKAEP-------------------------------PVHLNGAVHVSYKSKP---------------------------------------------NSPILHRPPKRVKTEAAPS----------------------------------------------------------------------------------------------------------------------------------------------**

**6_Pan_troglodytes PAWDGPRVLERALPPLP-----------------------------------------------------------RPPT--PKLEPKEES-------------------------------PTRINGSIPAGPKQEP----CA------------------------QHNGSEPASPKRERPTSPAPHRPPKRVKAEAVPS----------------------------------------------------------------------------------------------------------------------------------------------**

**6_Sus_scrofa PAWDGPRVLERALPPLP-----------------------------------------------------------RPPA--PKLEPKEEA-------------------------------PAQFNCPAPASTKQEPKTEPCA------------------------QHNGSGPTSPKREQLDSPAPHKPPKRAKAEVIPS----------------------------------------------------------------------------------------------------------------------------------------------**

**6_Macaca_mulatta PAWDGPHVLERALPPLP-----------------------------------------------------------RPPT--PKLEPKEES-------------------------------PTRINGSIPAGPKQEP----CA------------------------QHNGSEPASPKRERPTSPAPNRPPKRVKAEAVPS----------------------------------------------------------------------------------------------------------------------------------------------**

**6_Rattus_norvegicus PTWDGPRVLEKALPPLP-----------------------------------------------------------RPVA------PKAEP-------------------------------PVHLNG----SYKPKP---------------------------------------------DSPVPHRPPKRVKTEAAAS----------------------------------------------------------------------------------------------------------------------------------------------**

**6_HUMAN PAWDGPRVLERALPPLP-----------------------------------------------------------RPPT--PKLEPKEES-------------------------------PTRINGSIPAGPKQEP----CA------------------------QHNGSEPASPKRERPTSPAPHRPPKRVKAKAVPS----------------------------------------------------------------------------------------------------------------------------------------------**

SIRT7PHYSCOM E------QNPSLVSS--------------------------------------------------------------NRALEHDVGVHEDM-------------------------------SSECYDQKLEEFERSFMVQTT--------------------------------------SFAP---------RT----------------------------------------------------------------------------------------------------------------------------------------------------

**7_Ciona_intestinalis AVYFQHTDSLWGLARQPKLNELSTFTTTSIT---------------------------------------------YPISTAHQSSLSEQSNIINTHVCSANRTASWPGPALIPQPIESPNFLSDTNRENVLDEKPYLVPAVTHSQYPSASSQVTGQSVVSVADMSSKVEQQTLSQQCVVSPAPSWFGKGYRSKVNKKNGFKKRGKRKLS--------------------------------------------------------------------------------------------------------------------------------------**

**7_Apis_mellifera PQYNRAKDPIFFHAV--------------------------------------------------------------RLRNNEQYTTSQPC-------------------------------LEEKR-------------------------------------------------------------MHFTQKSN----------------------------------------------------------------------------------------------------------------------------------------------------**

**7_Danio_Rerio PVYSRLQDPIFSMAK--------------------------------------------------------------PLSPQEQKSHSRKE-------------------------------IAPPSALEEVSQSAPPQGEGPA-------------------------------------VQGGWFGRGYSKGRRKK---------KSS--------------------------------------------------------------------------------------------------------------------------------------**

**7_Xenopus_tropicalis PVYDRSQDPIFSLAV--------------------------------------------------------------PLHSSEEQSHTRKP-------------------------------IKTADSHNIIPEQEQPVVEQP--------------------------------------VSGGWFGKGYTKGRRMR---------R----------------------------------------------------------------------------------------------------------------------------------------**

**7_Bos_taurus PRYSRWQDPIFSLAT--------------------------------------------------------------PLRAGEEGSHSRKS-------------------------------LCRSREEPGPGDRGAPLSSAP--------------------------------------ILGGWFGRGCTKRTKRK---------KVT--------------------------------------------------------------------------------------------------------------------------------------**

**7_Canis_familiaris PPYSRWQDPIFTLAT--------------------------------------------------------------PLRAGEEGSHSRKS-------------------------------LCRSREDPPPGDRGAALSSAP--------------------------------------VLGGWFGRGCAKRTKRR---------KIT--------------------------------------------------------------------------------------------------------------------------------------**

**7_Equus_caballus PPYSRWQDPIFSLAT--------------------------------------------------------------PLRAGEEGSHSRKS-------------------------------LCRSREEPPPGDRGSPLSSAP--------------------------------------VLGGWFGRGCAKRTKRK---------KVL--------------------------------------------------------------------------------------------------------------------------------------**

**7_Macaca_mulatta PAYSRWQDPIFSLAT--------------------------------------------------------------PLRAGEEGSHSRKS-------------------------------LCRSREEAPPGDRGAPLSSAP--------------------------------------ILGGWFGRGCTKRTKKK---------KVT--------------------------------------------------------------------------------------------------------------------------------------**

**7_Mus_musculus PVYNRWQDPIFSLAT--------------------------------------------------------------PLRAGEEGSHSRKS-------------------------------LCRSREEAPPGDQSDPLASAPP-------------------------------------ILGGWFGRGCAKRAKRK---------KVA--------------------------------------------------------------------------------------------------------------------------------------**

**7_Pan_rRoglodytes PAYSRWQDPIFSLAT--------------------------------------------------------------PLRAGEEGSHSRKS-------------------------------LCRSREEAPPGDRGAPLSSAP--------------------------------------ILGGWFGRGCTKRTKRK---------KVT--------------------------------------------------------------------------------------------------------------------------------------**

**7_aRattus_norvegicus PVYNRWQDPIFSLAT--------------------------------------------------------------PLRAGEEGSHSRKS-------------------------------LCRSREEPPPGDQSAPLASATP-------------------------------------ILGGWFGRGCAKRAKRK---------KAA--------------------------------------------------------------------------------------------------------------------------------------**

**7_Sus_scrofa PPYSRWQDPIFSLAT--------------------------------------------------------------PLRAGEEGSHSRKS-------------------------------LCRSREEPGPEDRGAPRSSAP--------------------------------------ALGGWFGRGCTKRTKRK---------KVT--------------------------------------------------------------------------------------------------------------------------------------**

**7_HUMAN PAYSRWQDPIFSLAT--------------------------------------------------------------PLRAGEEGSHSRKS-------------------------------LCRSREEAPPGDRGAPLSSAP--------------------------------------ILGGWFGRGCTKRTKRK---------KVT--------------------------------------------------------------------------------------------------------------------------------------**

**D) (SECOND PART: continuing from the precedent to the end at right)**

**1.HyDRa_magnipapillata ------------------------------------------QSDSPQIDEG-----------------------------------------------------------------------------------------------------------------------------IYFLPIPPNKFVFHGAELYTEDCP----------------------------------------------------------------------------------------------------------------------------------------------**

**1.StRongylocEntRotus AQRNSAAEQYMLFQRTLDSIGNSSTGASSLDVMAGNDSVASGESESTQTHVASDC----------------------------------------KEPSTDVKAEAEDLGLCVNETDRTNSQDDLNSEVGNVSSSSGSVLEHREQASPAPDSEAKPSPSTSAAAKTPRATISTQLKESSFLFIPPMRYVFHGAEVFLSDDDNEIEHGLEGLNKDMDINDLHNELENGDLHNDLGSGDLHNELENGDLHNHLHNGDDLPDLSEEDHSPAKEKTVSLDDLTPTVPSTDRETSQYGQSASKAQCVPETNGRDGSPFEAPSGETVYTHIDLPVTP------------**

**1.BRugia_malayi ---------------------------------------------------------------------------------------------------------------------------------------------------------------MPSMWDARYISVGSKLPVDGYLFIVPNKNIFPGAEIYYDKDDDIFRQLPEHYHSSAASSSSDSGESVTNQEEIYCNVGVRTMSLEESSPARAGSESDERSSSCPPKMDLETKYSAESSCAGNRLSVRLEPFLQTITVQTREETTHGLVRTSTSNHLLL--------------------------**

**1.aScHistosoma_mansoni --------------------------------------------------------------------------------------------------------------------------------------------------------------------EKTYTSTVVSSSDNSSELMNKNSSNDAVIEVSEDEEDGECVWEVASSLPR---------------------------------------------------------------------------------------------------------------------------------**

**1.ScHistosoma_japonicum -------------------------------------------------------------------------------------------------------------------------------------------------------------------------------------------------------------------------------------------------------------------------------------------------------------------------------------------------------**

**1.AcyRtHosipHon_pisum NLSTD-----------------------------------STRDSGIDPDD------------------------------------------------------------------------------------------------------------------PQKSSLAAYLPSNKYYMLKKRRYMFSGAEVD------------LNDMNDD---------------SETESDSSEKSETPPLH------------------------------------------------------------------------------------------------**

**1.TRibolium_castanEum HMSVD-----------------------------------SARDSGIGDNS--------------------------------------------NFTDLETKYDDTSDENPDLGEYNTNTNTASDPQFTNISNDEANKTNTYLESTTNTSDLKG------FWQPKIKKSLAERLPPKSFYLVKPSRYIFPGAEIYYDPDE------KFGYYEGS---------------SSSNNSDSESENGEPVSN-----------------------------------------------------------------------------------------------**

**1.Apis_mEllifERa EIISE-----------------------------------SSIDYKFHTVSVESTSKDIGKIYSLEECQVFPRIIEISSESALLDSTLKPHHCVENRTSLKMNNDYSTMNSTEIEKTNFKPRQASIDSALDSGVGDSCNSVDSHEDKNSKEELKNGTLNQHCWHPKIRKSLAERLPENSYYQLAPGKYIFPGAEVYSDPEEYDHCSLSINSESSD---------------SDSDSSSIDEEEDDEVEEEEEEEEEEEQDEENERNENQVEDNDDKLKEKLGMKKEGHEEEINEKNIKEDVKGHRKIETKNNKIETENNQRKIMEREDERRNISKFHNENILEN**

**1.PEDiculus NVITQ-----------------------------------SLRDVRV--------------------------------------------------------------------------------------------------------------------------------------------------------------------------------------------------------------------------------------------------------------------------------------------------------**

**1.Danio_RERio DHANA------------------------------------EHTENTSAGH----------------------------------------------------------------------------------------------------------VNAEHIEHMSKD----HANPK----DDQSSLSVNEEELASPAAET--HALDSTEISAH---------------TERS-----KEADAVNTDDAACVK-------DEE-NTDRLR-----------VEMRRR----CWRSRICQSPISKRLG-----------------------------------**

**1.aNotHobRancHius_f VEMGD------------------------------------FKSQNSSFDY----------------------------------------------------------------------------------------------------------RKRYWVSRISRSPISKRLNPGQYLFQSPNRYLFHGAEVYSDSEEE--TSSSCGSDSDE---------------SECSPGGVEDDSDPEDVSEAAAAD-------GETRIKDMFR-----------NSANEE----ESSVQIDSSSEKAHSATNQA-------------------------------**

**1.bNotHobRancHius_K VEMGD------------------------------------FKSQNSSFDY----------------------------------------------------------------------------------------------------------RKRYWVSGISRSPISKRLNPGQYLFQSPNRYLFHGAEVYSDSEEE--TSSSCGSDSDE---------------SECSPGGVEDDSDPEDVSEAAAAD-------GETRIKDMFR-----------NSANEE----KSSVQIDSSSEKAHSATNQA-------------------------------**

**1.XEnopus_tRopicalis DQETA------------------------------------EKDTDIDSAK-------------------------------------------------------------------------------------------------------------DLESKYTKEQISKRLDSTQFLFLAPNRYIFHGAEVFSDSDED-LTSSSCGTNSDS---------------ESLLSPSLHEPIEEDSDTEECFHAK------YENETDTDNR-----------ADLERE--PERVVLYQSDDLLGIDGTTMNL-------------------------------**

**1.TaEniopygia_guttata MNSET------------------------------------MKENGSNDGE------------------------------------------------------------------------------------------------NKEKS---EILKKCWVNRSAKEQISKRLDGTQYLFLPPNRYIFHGAEVYSDSEDDIISSSSCGSSSES---------------GSCRSQSLD--VEDESEMEEFYNG-------IEDED-APER---EEEPGFGEDGAEQEELAAEESAETNEAAGTEHP-SNAL-------------------------------**

**1.Gallus_gallus MNSET------------------------------------MKENGTNNGE------------------------------------------------------------------------------------------------SKEKN---EIVKKCWVNRSAKEQISKRLDGTQYLFLPPNRYIFHGAEVYSDSEDDMISSSSCGSSSES---------------GSCHSQSLD--VEDESEIEEFYNG-------IEDED-APER---EVEAAFEEDGVEQD--AADESAYTNEAAGNDHPTSNKL-------------------------------**

**1.Equus_caballus GSPY-------------------------------------LKNVGSDTGE------------------------------------------------------------------------------------------------KNERTSVAEAVRKCWPARLAKEQISKRLDGNQYLFLPPNRYIFHGAEVYSDSEEDVLSSSSCGSNSDS---------------GTCQSPSLEEPMEDESEIEEFYNG-------LEDDADVNER---AGGTGFRADGSDQE--AVNETISTKQEATDINYSSNKS-------------------------------**

**1.Macaca_mulatta GNPD-------------------------------------LKNVGSSTGE------------------------------------------------------------------------------------------------KNERTSVAGTVRKCWPSRVAKEQISKRLDGNQYLFLPPNRYIFHGAEVYSDSEDDVLSSSSCGSNSDS---------------GTCQSPSLEEPMEDESEIEEFYNG-------LEDEPDIPER---AGGAGFGTDGDDQE--AINEAISMKQEVTDMNYPSNKS-------------------------------**

**1.MonoDElpHis_DomEstica E------------------------------------------DAGANGGE------------------------------------------------------------------------------------------------KNERTNVVETLRKCWPNRLAKEQISKRLDGNQYLFLPPNRYIFHGAEVYSDSEDDVLSSSSCGSNSDS---------------GTCRSPSLEEPMEDESEMEEFYNG-------LEEAD-GPERGGPACAC----DKEDQR--AVGEAASITDEAAAIDHPSSRL-------------------------------**

**1.ORnitHoRHyncHus EGLDR------------------------------------TKDAAASIGENKE---------------------------------------------------------------------------------------------RNERTSAAETLRKCWGSRLAKEQISKRLDGNQYLFLPPNRYIFHGAEVYSDSEDDILSSSSCGSNSDS---------------GTCHSPSLEEPLEDESEIEEFYNG-------LEDDAVGPERGGGGAAAGFGGDDDEQE--AVTEAVSVKVEAAEMDHSSNGL-------------------------------**

**1.Canis_familiaRis ESPD-------------------------------------LKNVGCNTGE------------------------------------------------------------------------------------------------KNERTSVADPVRKCWPARLAKEQISKRLDGNQYLFLPPNRYIFHGAEVYSDSEDDVLSSSSCGSNSDS---------------GTCQSPSLEEHLEDESEIEEFYNG-------LEDEADVNER---AGGTGFGIDGGDQE--AVNEAISMKQEATDTNYPSNKS-------------------------------**

**1.Bos_tauRus ESPD-------------------------------------LKNAVSNSGE------------------------------------------------------------------------------------------------KNERTSVAETVRKCWPARLAKEQISKRLDDNQYLFLPPNRYIFHGAEVYSDSEDDVLSSSSCGSNSDS---------------GTCQSPSLEEPMEDESENEEFYNG-------LEDDADVNER---AGGTVFEADGGDQE--AINEAISVKQEATCINYPSNKS-------------------------------**

**1.Sus_scRofa ESPG-------------------------------------LKNVACSNGE------------------------------------------------------------------------------------------------KNERTSVAETVRKCWPARLAKEQISKRLDGNQYLFLPPNRYIFHGAEVYSDSEDDVLSSSSCGSNSES---------------GTCQSPSLEEPMEDESEIEEFYNG-------LEDDADVNMR---AGGTGFGADGSDQE--AVNEAISMKQEATGVNYPSNKS-------------------------------**

**1.Rattus_noRvEgicus ENPD-------------------------------------FKAVGSSTGD------------------------------------------------------------------------------------------------KNERTSVAETVRKCWPNRLAKEQISKRLDGNQYLFVPPNRYIFHGAEVYSDSEDDALSSSSCGSNSDS---------------GTCQSPSLEEPLEDESEIEEFYNG-------LEDDADRPEC---AGGS--GADGGDQE--AVNEAIAMKQELTDVNCTPDKSEHY----------------------------**

**1.Mus_musculus ENPD-------------------------------------FKAVGSSTAD------------------------------------------------------------------------------------------------KNERTSVAETVRKCWPNRLAKEQISKRLEGNQYLFVPPNRYIFHGAEVYSDSEDDVLSSSSCGSNSDS---------------GTCQSPSLEEPLEDESEIEEFYNG-------LEDDTERPEC---AGGSGFGADGGDQE--VVNEAIATRQELTDVNYPSDKS-------------------------------**

**1.SIRT1_HUMAN ENPD-------------------------------------LKNVGSSTGE------------------------------------------------------------------------------------------------KNERTSVAGTVRKCWPNRVAKEQISRRLDGNQYLFLPPNRYIFHGAEVYSDSEDDVLSSSSCGSNSDS---------------GTCQSPSLEEPMEDESEIEEFYNG-------LEDEPDVPER---AGGAGFGTDGDDQE--AINEAISVKQEVTDMNYPSNKS-------------------------------**

719 747

**2_aAjEllomycEs_ER -----------------------------------------------------------------------------------------------------------------------------------------------------------------WREELEELWAQTNPEKAGLMEKEKEKPARSKNELLRDEIERLTEEVEHTLHISRAHEDRVRSELQKGKESEVGNGEDATDKREPTPTSQDDEDRVMKKGNEPSESEQLDPVPQQESKSETKSSDDCATKQENSSTAGREPNKESDQETER--------------------------------**

**2_aMicRomonas_CCMP1545 -----------------------------------------------------------------------------------------------------------------------------------------------------------------WGDELDELVRAG-----------VEKVKSPTIDR----------------------------------------------------------------------------------------------------------------------------------------------------**

**2_OstREococcus_tauRi -----------------------------------------------------------------------------------------------------------------------------------------------------------------WKEDLDALIKSC-----------EIRNKLAQEAPAECLNG----------------------------------------------------------------------------------------------------------------------------------------------**

**2_TRicHoplax_aDHaEREns -----------------------------------------------------------------------------------------------------------------------------------------------------------------WKEELLQLKERGHKILIEKYPQMVTKEAKVSLISSAPEQHSQAVESKSNADGNKESTEDEGSQNFDAIAVTQEHQIN---------------------------------------------------------------------------------------------------------**

**2_NEmatostElla_vEctEnsis -----------------------------------------------------------------------------------------------------------------------------------------------------------------WKAS-------------EPFAIV---------------------------------------------------------------------------------------------------------------------------------------------------------------**

**2_BRancHiostoma -----------------------------------------------------------------------------------------------------------------------------------------------------------------WKEQLEELVHREHTRIEAQ-------------------------------------------------------------------------------------------------------------------------------------------------------------------**

**2_StRongylocEntRotus -----------------------------------------------------------------------------------------------------------------------------------------------------------------WKKEMTVLVNKGE---AKKKKKGETESPTKDETAKTPTSATKDQKPKTSASPGKKS---SASPTKDKKPSSKKD------------------------------------------------------------------------------------------------------------**

**2_Ciona_intEstinalis -----------------------------------------------------------------------------------------------------------------------------------------------------------------WKNELKTMVCNQHKEIDQNSQATASKE-----KCPSKKSA----------------------------------------------------------------------------------------------------------------------------------------------**

**2_BRugia_malayi -----------------------------------------------------------------------------------------------------------------------------------------------------------------WKTELNKLIETELKKINEKXEKDEKNIKSTVTTIDNISINEEKNGKSIERXKSVDXKSNKL-------------------------------------------------------------------------------------------------------------------------**

**2_aScHistosoma_japonicum -----------------------------------------------------------------------------------------------------------------------------------------------------------------WKDDLLKLKEETDSR---------LIAQFVEKKSQQ--------------------------------------------------------------------------------------------------------------------------------------------------**

**2_ScHistosoma_mansoni -----------------------------------------------------------------------------------------------------------------------------------------------------------------WKDDLLRLKKETDSR---------LNEEFLAKKSQDKTNGQ---------------------------------------------------------------------------------------------------------------------------------------------**

**2_aCaligus_clEmEnsi -----------------------------------------------------------------------------------------------------------------------------------------------------------------WKEELISLMGGS--------------------------------------------------------------------------------------------------------------------------------------------------------------------------**

**2_Caligus_RogERcREssEyi -----------------------------------------------------------------------------------------------------------------------------------------------------------------WKEELISLMGGS--------------------------------------------------------------------------------------------------------------------------------------------------------------------------**

**2_LEpEopHtHEiRus -----------------------------------------------------------------------------------------------------------------------------------------------------------------WKEELESLMNK---------------------------------------------------------------------------------------------------------------------------------------------------------------------------**

**2_AEDEs_aEgypti -----------------------------------------------------------------------------------------------------------------------------------------------------------------MGDELREMIKREHAKLDAAQHKHFPVAEPNPTETASATASQNHDSDDVMIHDVQHAVDEPATNIAPVPDLVPVDINHHGEMKDLGEGKSTTVADGTKND-----------------------------------------------------------------------------------**

**2_Apis_mEllifERa -----------------------------------------------------------------------------------------------------------------------------------------------------------------WGDELKNLIKREYERLNR------G-------------------------------------------------------------------------------------------------------------------------------------------------------------**

**2_Bombyx_moRi -----------------------------------------------------------------------------------------------------------------------------------------------------------------WGDELRALVAREHERLDQ------ELLTASPHAPVLIPSEANAGPSASE-------------------------------------------------------------------------------------------------------------------------------------**

**2_DRosopHila -----------------------------------------------------------------------------------------------------------------------------------------------------------------WDQELQQLITSERKKLSGSQ----NSEELQQGKEKPQSDPDKMTSGDRDKKDASL-------------------------------------------------------------------------------------------------------------------------------**

**2_PEDiculus -----------------------------------------------------------------------------------------------------------------------------------------------------------------WKQEFDEIIKKSDEQENY----------MKSKGELFKFDMSNMRNIV---------------------------------------------------------------------------------------------------------------------------------------**

**2_TRibolium_castanEum -----------------------------------------------------------------------------------------------------------------------------------------------------------------WGDELKKLRQTEIEKIEK--------AEATPVKSSM--------------------------------------------------------------------------------------------------------------------------------------------------**

**2_Danio_RERio -----------------------------------------------------------------------------------------------------------------------------------------------------------------WKKELEEMVKREHALIDS--KDAKKTD-----KEASQSSKSAVAEAEKTDKTE---------------------------------------------------------------------------------------------------------------------------------**

**2_Salmo_salaR -------------------------------------------------------------------------------------------------------------------------------------------------------------------------------------------------------------------------------------------------------------------------------------------------------------------------------------------------------**

**2_XEnopus_laEvis -----------------------------------------------------------------------------------------------------------------------------------------------------------------WKAELEELVKKEHAEIDAAAEAAKKKE-----SKPDNSSKEESSAANTSDDKANN-------------------------------------------------------------------------------------------------------------------------------**

**2_Gallus_gallus -----------------------------------------------------------------------------------------------------------------------------------------------------------------WKKELQELVRKEHAAIDAVAAPEDTSSASGGDPTSRRGRTDGSGGRAESSGASSEQRGDGKEP-----------------------------------------------------------------------------------------------------------------------**

**2_BostauRus -----------------------------------------------------------------------------------------------------------------------------------------------------------------WKKELEDLVRKEHASIDAQSGSGASNP--ATSASPRNSPPPPTKEEPRTTEGEKPQ------------------------------------------------------------------------------------------------------------------------------**

**2_Canis_familiaRis -----------------------------------------------------------------------------------------------------------------------------------------------------------------WKKELEDLVRKEHAHIDAQAGSGAPNPSTSTSASPSKSPPP-AKEEARTKEGEKPQ------------------------------------------------------------------------------------------------------------------------------**

**2_Equuscaballus -----------------------------------------------------------------------------------------------------------------------------------------------------------------WKTTHS-------------GATKVPPP--SLSVSPSKSPPP-AKEEARTTEREKPQ------------------------------------------------------------------------------------------------------------------------------**

**2_RattusnoRvEgicus -----------------------------------------------------------------------------------------------------------------------------------------------------------------WKKELEDLVRREHANIDAQSGSQASNP--SATVSPRKSPPP-AKEAARTKEKEEH-------------------------------------------------------------------------------------------------------------------------------**

**2_Musmusculus -----------------------------------------------------------------------------------------------------------------------------------------------------------------WKKELEDLVRREHANIDAQSGSQAPNP--STTISPGKSPPP-AKEAARTKEKEEQQ------------------------------------------------------------------------------------------------------------------------------**

**2_SusscRofa -----------------------------------------------------------------------------------------------------------------------------------------------------------------WKKELEDLVRKEHASIDAQSGSGTPNP--TTSASPRKSPPP-AKAEARTSEGEKPQ------------------------------------------------------------------------------------------------------------------------------**

**2_HUMAN -----------------------------------------------------------------------------------------------------------------------------------------------------------------WKKELEDLVRREHASIDAQSGAGVPNP--STSASPKKSPPP-AKDEARTTEREKPQ------------------------------------------------------------------------------------------------------------------------------**

**3_Ciona_intEstinalis -----------------------------------------------------------------------------------------------------------------------------------------------------------------WDEFIKEVVDSNEKVLHVNDPPHEDSGISSASSTSSERKVIPPVDKNVKRYFHSLRSTPLPNNNKETGSGEPPHSYYEFMKHSIQNPPPKSTSALSKQRPPNKQHPPVIQYTRTILSKSKSFPRMRSSISVPSLYRGNKVETTSESETSDASSSSGVDS-----------------------**

**3_XEnopus_tRopicalis -----------------------------------------------------------------------------------------------------------------------------------------------------------------WQAELDQLMNS------------PTSGVCVMSSNGHMAWEKTNWV-----------------------------------------------------------------------------------------------------------------------------------------**

**3_Danio_RERioB -----------------------------------------------------------------------------------------------------------------------------------------------------------------WHTEIQTLMNS------------HENGLYSYISSS---------------------------------------------------------------------------------------------------------------------------------------------------**

**3_Gallus_gallus -----------------------------------------------------------------------------------------------------------------------------------------------------------------WNEEMQTLIQK-------------EKEKLDAKDK----------------------------------------------------------------------------------------------------------------------------------------------------**

**3_TaEniopygia_guttata -----------------------------------------------------------------------------------------------------------------------------------------------------------------WNKEMQTLIQK-------------EKEKLDAKDK----------------------------------------------------------------------------------------------------------------------------------------------------**

**3_Equus_caballus -----------------------------------------------------------------------------------------------------------------------------------------------------------------WTEEMQDLIQR-------------ETGKLDGQDR----------------------------------------------------------------------------------------------------------------------------------------------------**

**3_iso9Canis_familiaRis -----------------------------------------------------------------------------------------------------------------------------------------------------------------WREELQDLIQQ-------------ETEKLDGRDG----------------------------------------------------------------------------------------------------------------------------------------------------**

**3_Mus_musculus -----------------------------------------------------------------------------------------------------------------------------------------------------------------WTQELLDLMQR-------------ERGKLDGQDR----------------------------------------------------------------------------------------------------------------------------------------------------**

**3_aRattus_noRvEgicus -----------------------------------------------------------------------------------------------------------------------------------------------------------------WTQELQDLIQR-------------ENGKLDGQDG----------------------------------------------------------------------------------------------------------------------------------------------------**

**3_ORyctolagus_cuniculus -----------------------------------------------------------------------------------------------------------------------------------------------------------------WTEEMHDLIQR-------------ETRKLDGRDT----------------------------------------------------------------------------------------------------------------------------------------------------**

**3_MonoDElpHis_DomEstica -----------------------------------------------------------------------------------------------------------------------------------------------------------------WTQEIEDLVQR-------------ETSKVGQKGKEGRSSRARQQAKGLTASWGPRNNPGKKELAKVSRTGRA--------------------------------------------------------------------------------------------------------------**

**3_Macaca_mulatta -------------------------------------------------------------------------------------------------------------------------------------------------------------------------------------------------------------------------------------------------------------------------------------------------------------------------------------------------------**

**3_Sus_scRofa -----------------------------------------------------------------------------------------------------------------------------------------------------------------WTEEMQDLIQQ-------------ETGKFDGWDK----------------------------------------------------------------------------------------------------------------------------------------------------**

**3_HUMAN -----------------------------------------------------------------------------------------------------------------------------------------------------------------WTEEMRDLVQR-------------ETGKLDGPDK----------------------------------------------------------------------------------------------------------------------------------------------------**

**4_StigmatElla_DW4/3 -------------------------------------------------------------------------------------------------------------------------------------------------------------------------------------------------------------------------------------------------------------------------------------------------------------------------------------------------------**

**4_StRongylocEntRotus -------------------------------------------------------------------------------------------------------------------------------------------------------------------------------------------------------------------------------------------------------------------------------------------------------------------------------------------------------**

**4_AcyRtHosipHon_pisum -------------------------------------------------------------------------------------------------------------------------------------------------------------------------------------------------------------------------------------------------------------------------------------------------------------------------------------------------------**

**4_Apis_mEllifERa -------------------------------------------------------------------------------------------------------------------------------------------------------------------------------------------------------------------------------------------------------------------------------------------------------------------------------------------------------**

**4_DRosopHila -------------------------------------------------------------------------------------------------------------------------------------------------------------------------------------------------------------------------------------------------------------------------------------------------------------------------------------------------------**

**4_TRibolium_castanEum -------------------------------------------------------------------------------------------------------------------------------------------------------------------------------------------------------------------------------------------------------------------------------------------------------------------------------------------------------**

**4_Danio_RERio -------------------------------------------------------------------------------------------------------------------------------------------------------------------------------------------------------------------------------------------------------------------------------------------------------------------------------------------------------**

**4_XEnopus_tRopicalis -------------------------------------------------------------------------------------------------------------------------------------------------------------------------------------------------------------------------------------------------------------------------------------------------------------------------------------------------------**

**4_Gallus_gallus -------------------------------------------------------------------------------------------------------------------------------------------------------------------------------------------------------------------------------------------------------------------------------------------------------------------------------------------------------**

**4_TaEniopygia_guttata -------------------------------------------------------------------------------------------------------------------------------------------------------------------------------------------------------------------------------------------------------------------------------------------------------------------------------------------------------**

**4_MOUSE -------------------------------------------------------------------------------------------------------------------------------------------------------------------------------------------------------------------------------------------------------------------------------------------------------------------------------------------------------**

**4_Bos_tauRus -------------------------------------------------------------------------------------------------------------------------------------------------------------------------------------------------------------------------------------------------------------------------------------------------------------------------------------------------------**

**4_Equus_caballus -------------------------------------------------------------------------------------------------------------------------------------------------------------------------------------------------------------------------------------------------------------------------------------------------------------------------------------------------------**

**4_Pan_tRogloDytEs -------------------------------------------------------------------------------------------------------------------------------------------------------------------------------------------------------------------------------------------------------------------------------------------------------------------------------------------------------**

**4_Canis_familiaRis -------------------------------------------------------------------------------------------------------------------------------------------------------------------------------------------------------------------------------------------------------------------------------------------------------------------------------------------------------**

**4_Ovis_aRiEs -------------------------------------------------------------------------------------------------------------------------------------------------------------------------------------------------------------------------------------------------------------------------------------------------------------------------------------------------------**

**4_Macaca_mulatta -------------------------------------------------------------------------------------------------------------------------------------------------------------------------------------------------------------------------------------------------------------------------------------------------------------------------------------------------------**

**4_Mus_musculus -------------------------------------------------------------------------------------------------------------------------------------------------------------------------------------------------------------------------------------------------------------------------------------------------------------------------------------------------------**

**4_Rattus_noRvEgicus -------------------------------------------------------------------------------------------------------------------------------------------------------------------------------------------------------------------------------------------------------------------------------------------------------------------------------------------------------**

**4_Sus_scRofa -------------------------------------------------------------------------------------------------------------------------------------------------------------------------------------------------------------------------------------------------------------------------------------------------------------------------------------------------------**

**4_HUMAN -------------------------------------------------------------------------------------------------------------------------------------------------------------------------------------------------------------------------------------------------------------------------------------------------------------------------------------------------------**

**5_PERKinsus_ATCC -------------------------------------------------------------------------------------------------------------------------------------------------------------------------------------------------------------------------------------------------------------------------------------------------------------------------------------------------------**

**5_Ciona_intEstinalis -------------------------------------------------------------------------------------------------------------------------------------------------------------------------------------------------------------------------------------------------------------------------------------------------------------------------------------------------------**

**5_Apis_mEllifERa -------------------------------------------------------------------------------------------------------------------------------------------------------------------------------------------------------------------------------------------------------------------------------------------------------------------------------------------------------**

**5_Nasonia_vitRipEnnis -------------------------------------------------------------------------------------------------------------------------------------------------------------------------------------------------------------------------------------------------------------------------------------------------------------------------------------------------------**

**5_Anoplopoma_fimbRia -------------------------------------------------------------------------------------------------------------------------------------------------------------------------------------------------------------------------------------------------------------------------------------------------------------------------------------------------------**

**5_Danio_RERio -------------------------------------------------------------------------------------------------------------------------------------------------------------------------------------------------------------------------------------------------------------------------------------------------------------------------------------------------------**

**5_Salmo_salaR -------------------------------------------------------------------------------------------------------------------------------------------------------------------------------------------------------------------------------------------------------------------------------------------------------------------------------------------------------**

**5_TaEniopygia_guttata -------------------------------------------------------------------------------------------------------------------------------------------------------------------------------------------------------------------------------------------------------------------------------------------------------------------------------------------------------**

**5_Bos_tauRus -------------------------------------------------------------------------------------------------------------------------------------------------------------------------------------------------------------------------------------------------------------------------------------------------------------------------------------------------------**

**5_Canis_familiaRis -------------------------------------------------------------------------------------------------------------------------------------------------------------------------------------------------------------------------------------------------------------------------------------------------------------------------------------------------------**

**5_Pan_tRogloDytEs -------------------------------------------------------------------------------------------------------------------------------------------------------------------------------------------------------------------------------------------------------------------------------------------------------------------------------------------------------**

**5_Macaca_mulatta -------------------------------------------------------------------------------------------------------------------------------------------------------------------------------------------------------------------------------------------------------------------------------------------------------------------------------------------------------**

**5_Mus_musculus -------------------------------------------------------------------------------------------------------------------------------------------------------------------------------------------------------------------------------------------------------------------------------------------------------------------------------------------------------**

**5_ORnitHoRHyncHus -------------------------------------------------------------------------------------------------------------------------------------------------------------------------------------------------------------------------------------------------------------------------------------------------------------------------------------------------------**

**5_Rattus_noRvEgicus -------------------------------------------------------------------------------------------------------------------------------------------------------------------------------------------------------------------------------------------------------------------------------------------------------------------------------------------------------**

**5_Sus_scRofa -------------------------------------------------------------------------------------------------------------------------------------------------------------------------------------------------------------------------------------------------------------------------------------------------------------------------------------------------------**

**5_HUMAN -------------------------------------------------------------------------------------------------------------------------------------------------------------------------------------------------------------------------------------------------------------------------------------------------------------------------------------------------------**

**6_StRongylocEntRotus SGAVKRDAEGNIKVC----------------------------------------------------------------------------------------------------------------------------------------------------------------------------------------------------------------------------------------------------------------------------------------------------------------------------------------**

**6_LEpEopHtHEiRus -------------------------------------------------------------------------------------------------------------------------------------------------------------------------------------------------------------------------------------------------------------------------------------------------------------------------------------------------------**

**6_AcyRtHosipHon_pisum -------------------------------------------------------------------------------------------------------------------------------------------------------------------------------------------------------------------------------------------------------------------------------------------------------------------------------------------------------**

**6_Apis_mEllifERa -------------------------------------------------------------------------------------------------------------------------------------------------------------------------------------------------------------------------------------------------------------------------------------------------------------------------------------------------------**

**6_DRosopHila -------------------------------------------------------------------------------------------------------------------------------------------------------------------------------------------------------------------------------------------------------------------------------------------------------------------------------------------------------**

**6_Danio_RERio -------------------------------------------------------------------------------------------------------------------------------------------------------------------------------------------------------------------------------------------------------------------------------------------------------------------------------------------------------**

**6_XEnopus_tRopicalis -------------------------------------------------------------------------------------------------------------------------------------------------------------------------------------------------------------------------------------------------------------------------------------------------------------------------------------------------------**

**6_Gallus_gallus -------------------------------------------------------------------------------------------------------------------------------------------------------------------------------------------------------------------------------------------------------------------------------------------------------------------------------------------------------**

**6_Bos_tauRus -------------------------------------------------------------------------------------------------------------------------------------------------------------------------------------------------------------------------------------------------------------------------------------------------------------------------------------------------------**

**6_Canis_familiaRis -------------------------------------------------------------------------------------------------------------------------------------------------------------------------------------------------------------------------------------------------------------------------------------------------------------------------------------------------------**

**6_Mus_musculus -------------------------------------------------------------------------------------------------------------------------------------------------------------------------------------------------------------------------------------------------------------------------------------------------------------------------------------------------------**

**6_Pan_tRogloDytEs -------------------------------------------------------------------------------------------------------------------------------------------------------------------------------------------------------------------------------------------------------------------------------------------------------------------------------------------------------**

**6_Sus_scRofa -------------------------------------------------------------------------------------------------------------------------------------------------------------------------------------------------------------------------------------------------------------------------------------------------------------------------------------------------------**

**6_Macaca_mulatta -------------------------------------------------------------------------------------------------------------------------------------------------------------------------------------------------------------------------------------------------------------------------------------------------------------------------------------------------------**

**6_Rattus_noRvEgicus -------------------------------------------------------------------------------------------------------------------------------------------------------------------------------------------------------------------------------------------------------------------------------------------------------------------------------------------------------**

**6_HUMAN -------------------------------------------------------------------------------------------------------------------------------------------------------------------------------------------------------------------------------------------------------------------------------------------------------------------------------------------------------**

**7_Ciona_intEstinalis -------------------------------------------------------------------------------------------------------------------------------------------------------------------------------------------------------------------------------------------------------------------------------------------------------------------------------------------------------**

**7_Apis_mEllifERa -------------------------------------------------------------------------------------------------------------------------------------------------------------------------------------------------------------------------------------------------------------------------------------------------------------------------------------------------------**

**7_Danio_RERio -------------------------------------------------------------------------------------------------------------------------------------------------------------------------------------------------------------------------------------------------------------------------------------------------------------------------------------------------------**

**7_XEnopus_tRopicalis -------------------------------------------------------------------------------------------------------------------------------------------------------------------------------------------------------------------------------------------------------------------------------------------------------------------------------------------------------**

**7_Bos_tauRus -------------------------------------------------------------------------------------------------------------------------------------------------------------------------------------------------------------------------------------------------------------------------------------------------------------------------------------------------------**

**7_Canis_familiaRis -------------------------------------------------------------------------------------------------------------------------------------------------------------------------------------------------------------------------------------------------------------------------------------------------------------------------------------------------------**

**7_Equus_caballus -------------------------------------------------------------------------------------------------------------------------------------------------------------------------------------------------------------------------------------------------------------------------------------------------------------------------------------------------------**

**7_Macaca_mulatta -------------------------------------------------------------------------------------------------------------------------------------------------------------------------------------------------------------------------------------------------------------------------------------------------------------------------------------------------------**

**7_Mus_musculus -------------------------------------------------------------------------------------------------------------------------------------------------------------------------------------------------------------------------------------------------------------------------------------------------------------------------------------------------------**

**7_Pan_tRogloDytEs -------------------------------------------------------------------------------------------------------------------------------------------------------------------------------------------------------------------------------------------------------------------------------------------------------------------------------------------------------**

**7_aRattus_noRvEgicus -------------------------------------------------------------------------------------------------------------------------------------------------------------------------------------------------------------------------------------------------------------------------------------------------------------------------------------------------------**

**7_Sus_scRofa -------------------------------------------------------------------------------------------------------------------------------------------------------------------------------------------------------------------------------------------------------------------------------------------------------------------------------------------------------**

**7_HUMAN -------------------------------------------------------------------------------------------------------------------------------------------------------------------------------------------------------------------------------------------------------------------------------------------------------------------------------------------------------**
