# Supplementary material for: Genealogy of an ancient protein family: the Sirtuins, a family of disordered members
Source: BMC Evol Biol. 2013 Mar 5;13:60. doi: 10.1186/1471-2148-13-60 (PMC3599600; doi:10.1186/1471-2148-13-60)
Supplement: Additional file 3: Table S2 — Frequency of amino acids in DisProt and PDB25 datasets collecting disordered and ordered protein domains, respectively. [file 1471-2148-13-60-S3.doc]

**Table S2.** Frequency of amino acids in DisProt and PDB25 datasets collecting disordered and ordered protein domains, respectively.

| **Amino acid** | **Disordered** | **Ordered** |
| --- | --- | --- |
| A-Ala | 8.16 | 7.7 |
| C-Cys | 0.75 | 1.47 |
| D-Asp | 5.95 | 5.9 |
| E-Glu | 9.76 | 6.76 |
| F-Phe | 2.46 | 4.02 |
| G-Gly | 7.16 | 6.99 |
| H-His | 1.93 | 2.34 |
| I-Ile | 3.18 | 5.74 |
| K-Lys | 7.78 | 6.25 |
| L-Leu | 6.29 | 8.87 |
| M-Met | 1.8 | 2.23 |
| N-Asn | 3.69 | 4.52 |
| P-Pro | 8.41 | 4.43 |
| Q-Gln | 4.93 | 4.01 |
| R-Arg | 5.06 | 4.7 |
| S-Ser | 8.87 | 6.13 |
| T-Thr | 5.44 | 5.54 |
| V-Val | 5.35 | 7.03 |
| W-Trp | 0.6 | 1.46 |
| Y-Tyr | 2.08 | 3.54 |
